# A closed-loop multi-level model of glucose homeostasis

**DOI:** 10.1371/journal.pone.0190627

**Published:** 2018-02-08

**Authors:** Cansu Uluseker, Giulia Simoni, Luca Marchetti, Marco Dauriz, Alice Matone, Corrado Priami

**Affiliations:** 1 The Microsoft Research – University of Trento Centre for Computational and Systems Biology (COSBI), Rovereto (TN), Italy; 2 Division of Endocrinology, Diabetes and Metabolism, Department of Medicine, University of Verona, Verona, Italy; 3 Department of Computer Science, University of Pisa, Pisa, Italy; International University of Health and Welfare School of Medicine, JAPAN

## Abstract

**Background:**

The pathophysiologic processes underlying the regulation of glucose homeostasis are considerably complex at both cellular and systemic level. A comprehensive and structured specification for the several layers of abstraction of glucose metabolism is often elusive, an issue currently solvable with the hierarchical description provided by multi-level models. In this study we propose a multi-level closed-loop model of whole-body glucose homeostasis, coupled with the molecular specifications of the insulin signaling cascade in adipocytes, under the experimental conditions of normal glucose regulation and type 2 diabetes.

**Methodology/Principal findings:**

The ordinary differential equations of the model, describing the dynamics of glucose and key regulatory hormones and their reciprocal interactions among gut, liver, muscle and adipose tissue, were designed for being embedded in a modular, hierarchical structure. The closed-loop model structure allowed self-sustained simulations to represent an ideal *in silico* subject that adjusts its own metabolism to the fasting and feeding states, depending on the hormonal context and invariant to circadian fluctuations. The cellular level of the model provided a seamless dynamic description of the molecular mechanisms downstream the insulin receptor in the adipocytes by accounting for variations in the surrounding metabolic context.

**Conclusions/Significance:**

The combination of a multi-level and closed-loop modeling approach provided a fair dynamic description of the core determinants of glucose homeostasis at both cellular and systemic scales. This model architecture is intrinsically open to incorporate supplementary layers of specifications describing further individual components influencing glucose metabolism.

## Introduction

The maintenance of glucose homeostasis within a narrow physiological range is an essential component of human metabolism and it is finely regulated by complex mechanisms controlling insulin secretion and action. A disruption in the governance of the glucose-insulin system can lead to variable degrees of altered glucose regulation, which may ultimately result in overt diabetes mellitus. According to the most recent estimates, diabetes mellitus affects over 415 millions individuals worldwide [1], is characterized by severe cardiovascular complications leading to early death [2], and its prospective incidence trends highlight it as a global burden of pandemic proportion. Most diabetes cases are classified as type 2 diabetes mellitus (T2DM), which shows progressive loss of insulin secretion on the background of insulin resistance [3]. After decades of investigations, it is becoming clear that diabetes is a complex and highly heterogeneous disease [4], which still hampers a comprehensive understanding of the etiologic processes at the level of individual organs or tissues, and involving subcellular derangements ultimately affecting the whole body metabolism.

In this context, a number of mathematical models of glucose and insulin dynamics have been developed to allow the description and interpretation of such processes, which are often not accessible to direct measurement *in vivo* [5]. These models usually apply ordinary differential equations (ODEs) or delay differential equations (DDEs) to describe the physiology of the glucose-insulin system in different experimental conditions and with varying degrees of detail [6–14]. Originally built upon the parsimony principle in order to soften the complexity of experimental protocols and computational efforts, the first modeling milestone is represented by the so called “minimal model” [8], which was originally applied to estimate insulin sensitivity by inspecting the time courses of insulin and glucose after an intravenous glucose tolerance test (IVGTT).

Minimal models of glucose homeostasis have then been extended and applied to more physiological experimental conditions, and integrating a broader range of variables, including several hormones and regulatory elements such as ghrelin [9], glucagon [10] and incretins [9,11,15]. However, at present, there is not a comprehensive and structured model, which summarizes the dynamics of glucose absorption according to the regulation performed by insulin and other hormones at the subcellular level. This is possibly due to the constraints inherent to the reductionist modeling approach applied so far. The hierarchical descriptions provided by multi-level models may be a valid option to overcome these limits. This approach, also referred to as hierarchical modeling, is a recent advancement and a promising trend in the mathematical modeling of biological mechanisms, because it allows a given biological phenomenon to be described by simultaneously accounting for several (hierarchical) levels of abstraction [16].

Recent applications of multi-level models have been proposed to describe the pathophysiology of beta-cells in the endocrine pancreas [17], and the whole body effects of the altered insulin signaling cascade in adipocytes [18], while other applications described the effects of inflammation on the onset of T2DM and its complications [19]. The holistic approach of hierarchical modeling identifies subcellular processes, specific cell subtypes and tissues, organs and the whole body as strictly interconnected layers, where physiological variations occurring at any level would affect the dynamics elsewhere in the stacked constitutive elements.

A comprehensive, hierarchical description may be reasonably applied to T2DM, because the phenotypic hallmark of hyperglycaemia is the consequence of alterations involving complex hormonal and signaling networks, individual tissues and cell subtypes. Recently, Chew *et al*. [20] proposed a model of the glucose regulatory system combined with the insulin signaling model of Sedaghat *et al*. [21], while Nyman *et al*. [18] combined the organ level model of Dalla Man *et al*. [22] with three different detailed versions of insulin signaling in the adipocytes. The most detailed version includes the model from Kiselyov *et al*. for the description of insulin binding to its receptor [23].

In this study, we moved one step forward, as we applied a multi-level modeling approach to build a closed-loop whole-body model of glucose homeostasis. The closed-loop structure was designed to allow self-sustained simulations, fostering the investigation of the biological system in its components, while providing a way to test regulative phenomena that work at different time scales and possibly have a delayed effect on the overall system dynamics. The model was tested *in silico* in both the conditions of normal glucose regulation (NGR) and T2DM.

Since insulin resistance constitutes one of the key pathophysiologic determinants of T2DM, and given the increasing relevance of the adipose tissue as an endocrine organ influencing systemic energy balance and glucose homeostasis [24,25], we chose the adipose tissue (abstracted as adipocyte) as the compartment linking the cellular layer and the whole-body level of the model. The proposed whole-body model was therefore integrated with the most recent insulin signaling model proposed by Nyman *et al*. [26], that provides a detailed specification of the intracellular signaling cascade in the adipocytes.

Although exploratory in nature, the presented hierarchical architecture of glucose homeostasis is amenable to further extensions, such as the molecular descriptions of other organs and tissues that are here considered only at the whole-body level.

### Modeled physiology

To allow a better understanding of the model we report below a brief description of the represented physiology.

**Fig 1:**
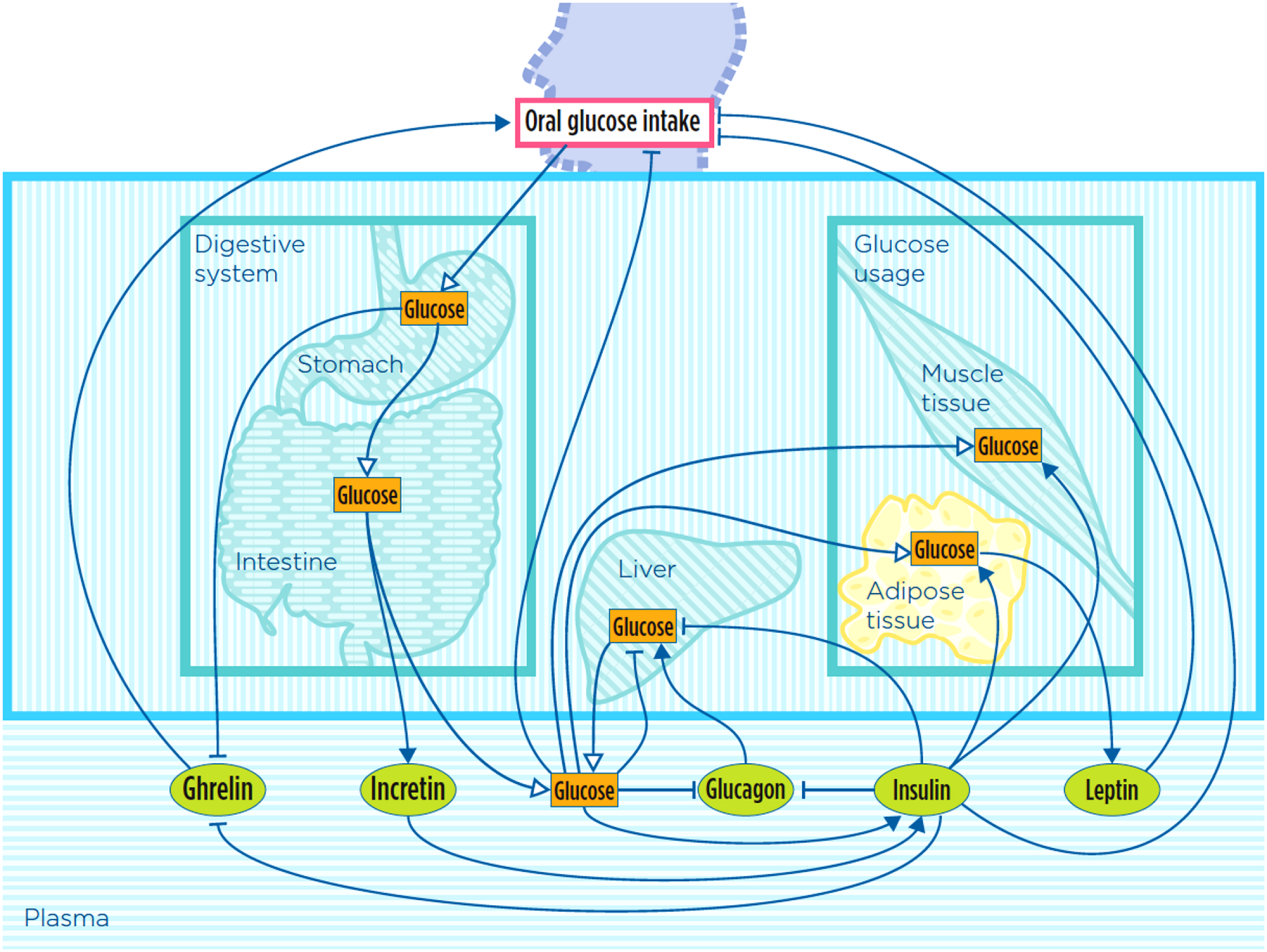
Graphical representation of the whole-body glucose metabolism as considered in our model, according to the notation introduced in [62]. Only the organs/tissues for which a variable has been explicitly included in the model are depicted in the figure (other key organs/tissues of glucose metabolism, like pancreas and brain, are not displayed in the figure even if their effect has been indirectly taken into account in model equations, see Results and S1 File for details). Adipose tissue is colored in yellow to highlight that it is the part for which a model at the cellular level is also provided (see Fig 2). Green ovals (hormones) and orange rectangles represent model variables; arrows represent mass transfer (white head), stimulation (black head) and inhibition (T head).

After oral glucose intake at time 0, glucose transits to the stomach and then to the intestine, where it is absorbed to plasma (Fig 1). These events induce endogenous insulin secretion, which is amplified by a concomitant increase in the circulating levels of the incretin hormones [15]. The incretins potentiate the release and the *de novo* synthesis of insulin from pancreatic beta cells, thus contributing to the proper glucose disposal in peripheral tissues and to maintain plasma glucose levels within the physiological range [9]. Therefore, insulin release is modeled here as a consequence of direct effects exerted by the glucose and indirect effects mediated by the incretins.

Glucose can be produced endogenously, from glycogen breakdown in the liver during fasting conditions. This mechanism is stimulated by glucagon, a hormone secreted from the alpha cells of the endocrine pancreas at low plasma glucose levels, and it is suppressed by hyperglycemia and hyperinsulinemia [27–29]. Endogenous glucose production is suppressed by high levels of both glucose and insulin [22,29,30]. Glucose tissue uptake happens when insulin binds to its cell receptors, mainly in adipocytes and muscle cells, where glucose is stored as glycogen [31]. Glucose uptake in adipocytes drives leptin release, which inhibits hunger and thus feeding (*i*.*e*. oral glucose intake). Hunger is here defined as the amount of food needed by organism [32]. The latter is also inhibited by high levels of plasma glucose and insulin. Ghrelin, which counteracts leptin, is secreted from the empty stomach, stimulating oral glucose intake [33], and it is inhibited by insulin [9,34].

In order to build a closed-loop model, oral glucose intake is represented as the amount of glucose needed from the organism, and it is computed considering the current levels of leptin, plasma glucose, insulin and ghrelin.

**Fig 2:**
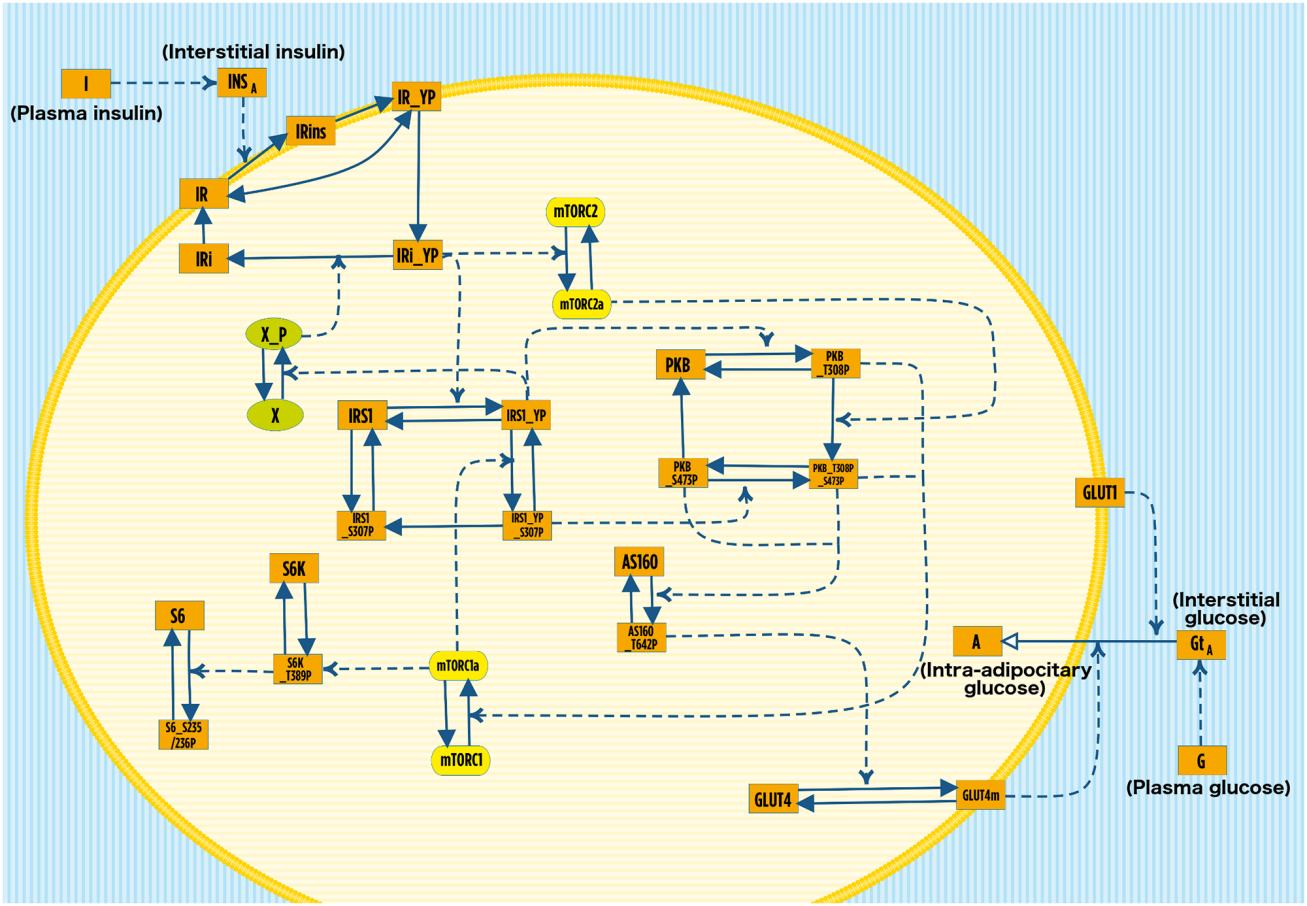
Graphical representation of the model describing the insulin signaling in adipocytes at the cellular level, according to the notation introduced in [62]. Solid arrows represent state modification, while dashed arrows indicate reaction stimulation. Protein complexes are colored in yellow, green ovals represent the active and inactive feedback protein, while the orange rectangles represent all the other components of the cellular model. The plasma membrane of the adipose cell is represented in yellow and it separates the cytosol (light yellow horizontal lines) from the interstitial fluid (blue and white vertical lines). The variables I and G indicate insulin and glucose concentration in plasma (compartment not represented), which regulate the amount of interstitial insulin (INS_A_) and glucose (Gt_A_), respectively. For the sake of simplicity, we highlighted the five variables linking the cellular level to the whole body description (namely plasma insulin, interstitial insulin, plasma glucose, interstitial glucose and intra-adipocitary glucose) by adding the corresponding names in parenthesis.

Plasma insulin and glucose are the variables linking the whole-body level to the cellular one (Fig 2). The connection between the levels is designed to work through the interstitial fluid, which has not been modeled as a separate compartment, but rather assuming that the interstitial concentrations of insulin and glucose (*i*.*e*. the amount surrounding the cell) would proportionally correspond to those of insulin and glucose in the plasma. The input of the cellular model is interstitial insulin, which binds its receptor on the cell membrane and prompts the auto-phosphorylation of the receptor and its endocytosis. The internalized phosphorylated receptor starts a cascade of phosphorylation and activation events, according to the model introduced by Nyman *et al*. [26], here simplified in some parts according to [35]. The model takes into account key actors, such as insulin receptor substrate 1 (IRS1), feedback protein X_P, PKB, mTORC1 and mTORC2 complexes, P70 ribosomal S6 kinase, ribosomal protein S6 and Akt substrate (AS160), which regulate the translocation of GLUT4 from the cytosol to the plasma membrane. The output of the cellular model is connected with the whole-body model: the amount of glucose uptake by the adipocyte (intra-adipocitary glucose) is regulated by the amount of GLUT1 and GLUT4 on the plasma membrane [18,26].

Further details on the background physiology of whole-body glucose metabolism and the insulin signaling cascade within the adipocytes could be retrieved in S1 File.

## Results

In the present work, we introduce a closed-loop multi-level mathematical model describing glucose homeostasis in NGR and T2DM conditions. The proposed model is composed of a set of ODEs defining the dynamics of state variables in minutes.

### Whole-body model equations

The whole-body model is described by Eqs (1) to (14), as follows. Eq (1) describes stomach glucose dynamics (S):

$$\frac{\text{dS}\left(\text{t}\right)}{\text{dt}}\;=\;{\text{b}}_{9}\text{H}\left(\text{t}\right)-{\text{b}}_{8}\text{S}\left(\text{t}\right)$$ (1)

The first term represents ingested glucose, which depends on glucose intake (H, Eq (12)) and on rate b_9_. The second term models stomach emptying and depends on the amount of stomach glucose and transfer rate b_8_, according to [9,15].

Eq (2) models intestine glucose transit, as described by Toghaw *et al*. [9]:

$$\frac{\text{dL}\left(\text{t}\right)}{\text{dt}}\;=\;{\text{b}}_{8}\text{S}\left(\text{t}\right)-{\text{b}}_{10}\text{L}\left(\text{t}\right)$$ (2)

The first term represents the glucose entry from the stomach, which coincides with the amount of glucose exiting the stomach in Eq (1). The second term accounts for glucose absorption into the plasmatic compartment, which depends on the amount of glucose in the intestine (L) and on rate b_10_.

The dynamics of plasma glucose concentration (G) is described in Eq (3):

$$\frac{\text{dG}\left(\text{t}\right)}{\text{dt}}\;=\;\text{f}\frac{{\text{b}}_{10}\text{L}\left(\text{t}\right)}{\text{v}}+\text{f}\frac{{\text{b}}_{5}\text{C}\left(\text{t}\right)}{\text{v}}-{\text{b}}_{1}\text{G}\left(\text{t}\right)-{\text{b}}_{3}\text{I}\left(\text{t}\right)\text{G}\left(\text{t}\right)$$ (3)

The first term represents plasma glucose appearance from the intestine, where b_10_ is the intestine to plasma transfer rate, v is the glucose distribution volume and f is a fraction of absorption accounting for the part of glucose lost in the transfer [9]. The second term describes liver glucose production, where glucose coming from the liver (C, Eq (7)) is multiplied by the transfer rate b_5_ and by f/v, similarly to the first term. Terms 3 and 4 represent blood glucose elimination through insulin-independent and insulin-dependent mechanisms, respectively. The third term models glucose uptake from the brain and other tissues such as blood cells, renal medulla, splanchnic tissues, which is insulin independent and proportional to G [36]. The last term models glucose uptake from adipose and muscle tissues, which depends on both G and plasma insulin concentration (I, Eq (4)) [9,15].

Eq (4) represents the dynamics of plasma insulin concentration (I):

$$\frac{\text{dI}\left(\text{t}\right)}{\text{dt}}\;=\;{\text{b}}_{4}\text{G}\left(\text{t}\right)+\text{cW}\left(\text{t}\right)\text{G}\left(\text{t}\right)-{\text{b}}_{2}\text{I}\left(\text{t}\right)$$ (4)

The first and second terms are simplifications of insulin dynamics described by Toghaw *et al*. [9]. The first term describes glucose-dependent insulin secretion, where b_4_ is glucose dependent insulin secretion rate. The second term represents incretin dependent insulin secretion, which is proportional to plasma glucose and incretin concentration (W, see Eq (5)), c being the incretin dependent insulin secretion rate. The last term represents insulin elimination, which depends on insulin and on its disappearance rate constant (b_2_).

Eq (5) describes the variation of plasma incretin concentration (W) [9,34]:

$$\frac{\text{dW}\left(\text{t}\right)}{\text{dt}}\;=\;{\text{b}}_{6}\text{L}\left(\text{t}\right)-{\text{b}}_{7}\text{W}\left(\text{t}\right)+\text{s}$$ (5)

The first term accounts for incretin appearance due to glucose transit through the intestine (L): it depends on L and on incretin secretion rate b_6_. The second term represents incretin elimination, depending on W and their disappearance rate constant b_7_. The last term is the constant incretin appearance rate (s).

In Eq (6) the dynamics of plasma glucagon (E) is described, according to Sulston *et al*. [29]:

$$\frac{\text{dE}\left(\text{t}\right)}{\text{dt}}\;=\;{\text{c}}_{0}+\frac{{\text{c}}_{1}}{{\text{c}}_{2}+\text{I}\left(\text{t}\right)e}\left({\text{G}}_{\text{e}}-\text{G}\left(\text{t}\right)\right)\text{u}\left({\text{G}}_{\text{e}}-\text{G}\left(\text{t}\right)\right)-{\text{c}}_{3}\text{E}\left(\text{t}\right)$$ (6)

The first term models the basal level of glucagon secretion, c_0_, which happens at normal fasting glucose levels. The second term represents the dependency of glucagon secretion from plasma glucose and insulin concentrations, where u(G_e_−G(t)) indicates the Heaviside step function:

$$\text{u}\left({\text{G}}_{\text{e}}-\text{G}\left(\text{t}\right)\right)\;=\;\{\begin{array}{c} 1,\;{\text{G}}_{\text{e}}-\text{G}\left(t\right)\geq 0\\ 0,\;{\text{G}}_{\text{e}}-\text{G}\left(\text{t}\right)<0. \end{array}$$

When G is above the threshold G_e_, this part of glucagon secretion is suppressed, resulting in an equilibrium value of glucagon achieved when $\frac{\text{dE}(\text{t})}{\text{dt}}\;=\;0$, that is, when $E\;=\;\frac{c_{0}}{c_{3}}$. Otherwise, the term represents glucagon secretion in the α cells of the pancreas, with the secretion increasing at low glucose levels but being suppressed by high insulin levels, according to parameters c_1_ and c_2_. The parameter e models insulin effectiveness to represent the cell sensitivity to insulin action, which is compromised in insulin resistance and in T2DM: this rate will be at its maximum in the NGR condition while it is lower in T2DM according to [29]. We refer to Table C in S1 File for the values of e and G_e_, employed in the model in the NGR and T2DM condition. The last term describes plasma glucagon elimination, which depends on E and its degradation rate c_3_ [29].

The variable C, described in Eq (7), represents glucose mass in the liver ready to be secreted, which has been produced from glycogen breakdown:

$$\frac{\text{dC}\left(\text{t}\right)}{\text{dt}}\;=\;{\text{b}}_{23}-{\text{b}}_{25}\text{I}\left(\text{t}\right)\text{e}-{\text{b}}_{22}\text{G}\left(\text{t}\right)+{\text{b}}_{21}\text{E}\left(\text{t}\right)-{\text{b}}_{5}\text{C}\left(\text{t}\right)$$ (7)

The equation is obtained by combining the works of Dalla Man *et al*. [22] and Sulston *et al*. [29], where variable/parameter units have been converted in accordance to the model. The first term describes the basal rate of liver glucose production, b_23_. The second and third term represent the inhibiting effect of I and G on liver glucose production, according to the rate constants b_25_ and b_22_ and insulin effectiveness (e). The fourth term accounts for plasma glucagon (E) effect in stimulating glycogen breakdown, where b_21_ is the rate of liver glucose production, which is glucagon dependent [29]. The last term represents glucose transfer from liver to plasma according to the transfer rate b_5_ [9].

Eq (8) describes the dynamics of glucose mass in muscle tissue (M):

$$\frac{\text{dM}\left(\text{t}\right)}{\text{dt}}\;=\;0.1\frac{\text{v}}{\text{f}}{\text{b}}_{3}\text{G}\left(\text{t}\right)\text{I}\left(\text{t}\right)\text{e}-{\text{b}}_{27}\text{M}\left(\text{t}\right)$$ (8)

The first term represents glucose entry, which depends on plasma glucose and insulin concentrations, on insulin effectiveness (e) [29], and on the utilization rate b_3_. The scaling factors $0.1\frac{\text{v}}{\text{f}}$ have been introduced to convert plasma glucose concentration to a mass and to set muscle glucose uptake to 10% of whole body glucose uptake [36]. The last term in the equation represents muscle glucose elimination, which depends on M and on the elimination rate b_27_ [37].

Eq (9) represents the adipose tissue glucose mass (A):

$$\frac{\text{dA}\left(\text{t}\right)}{\text{dt}}\;=\;{\text{k}}_{8}\text{GLUT}4\text{m}(\text{t})\frac{{\text{Gt}}_{\text{A}}\left(\text{t}\right)}{{\text{KmG}4+\text{Gt}}_{\text{A}}\left(\text{t}\right)}+\text{GLUT}1\frac{{\text{Gt}}_{\text{A}}\left(\text{t}\right)}{{\text{KmG}1+\text{Gt}}_{\text{A}}\left(\text{t}\right)}-{\text{k}}_{\text{gluc}}\text{A}\left(\text{t}\right)$$ (9)

Eq (9) is one of the links between the whole body model and the cellular one and includes variables from both. The first two terms come from the model of Nyman *et al*. [18]. These terms represent glucose entry in adipocytes mediated by glucose transporter 1 (GLUT1) and by glucose transporter 4 at the adipocyte membrane (GLUT4m). GLUT1 and GLUT4m are variables of the cellular model. GLUT1 does not depend on time in Eq (9) because its amount is assumed to be constant according to [18,26]. Both terms depend on interstitial glucose concentration (Gt_A_, see Eq (14)), where KmG4 and KmG1 are two parameters modeling the saturation of glucose internalization. The last term of the equation represents glucose elimination from the adipose tissue, which depends on the amount of internalized glucose and on the elimination rate k_gluc_.

Eq (10) describes the dynamics of plasma leptin (Y):

$$\frac{\text{dY}\left(\text{t}\right)}{\text{dt}}\;=\;{\text{b}}_{13}\text{A}\left(\text{t}\right)\text{Fat}-{\text{b}}_{14}\text{Y}\left(\text{t}\right)$$ (10)

The first term represents leptin secretion, which depends on the amount of glucose in the adipose tissue (A)[38,39], on leptin secretion rate b_13_ and on the Fat parameter (the average total fat mass in humans [40]). The second term models leptin degradation, which depends on Y and on the elimination rate b_14_ according to [40,41].

The dynamics of ghrelin concentration in plasma (Q) is described in Eq (11):

$$\frac{\text{dQ}\left(\text{t}\right)}{\text{dt}}\;=\;{\text{b}}_{12}{\text{exp}}^{-\text{lS}\left(\text{t}\right)}{\text{exp}}^{-\text{mI}\left(\text{t}\right)}-{\text{b}}_{11}\text{Q}\left(\text{t}\right)$$ (11)

The first term represents ghrelin secretion, which is modeled as being exponentially inhibited by both S and I. The term also depends on the parameters l (the S dependent decay rate), b_12_ (ghrelin secretion rate) and m (the I dependent decay rate) [9,34]. The last term of the equation accounts for ghrelin linear elimination, which depends on the rate b_11_ [9,34].

Eq (12) describes glucose intake (H):

$$\frac{\text{dH}\left(\text{t}\right)}{\text{dt}}\;=\;\frac{{\text{b}}_{17}\text{Q}\left(\text{t}\right)}{{\text{b}}_{18}\text{Y}\left(\text{t}\right)+1}{\text{exp}}^{-\text{rI}\left(\text{t}\right)}-{\text{b}}_{19}\text{G}\left(\text{t}\right)\text{H}\left(\text{t}\right)-{\text{b}}_{9}\text{H}\left(\text{t}\right)$$ (12)

H represents the amount of glucose needed from the body. In order to build a closed-loop model, glucose intake has been modeled equal to this amount, which can be thought of as the hunger signal. The latter has been initially introduced in the rat model of Jacquier *et al*. [32] and the corresponding equation has been here adapted to model human physiology. The first term represents the effect of plasma insulin (I), leptin (Y) and ghrelin (Q) on hunger. I and Y inhibit food intake while Q increases it. The effect of leptin and ghrelin is mediated by parameters b_18_ and b_17_, respectively, according to [32,42]. Insulin exponentially inhibits H through the r parameter, modeling the negative effect on appetite that arises when I is high [43,44]. The second term describes glucose intake reduction that depends on plasma glucose and on H itself [45]. The last term accounts for glucose absorption to the stomach, which depends on the amount of ingested glucose and on glucose transfer rate b_9_.

Eqs (13) and (14) link the whole body model with the cellular one, by describing interstitial insulin (INS_A_) and interstitial glucose (Gt_A_) surrounding the adipocytes:

$$\frac{{\text{dINS}}_{\text{A}}\left(\text{t}\right)}{\text{dt}}\;=\;-{\text{p}}_{2\text{U}}{\text{INS}}_{\text{A}}\left(\text{t}\right)+{\text{p}}_{2\text{U}}\left(\text{I}\left(\text{t}\right)-{\text{I}}_{\text{b}}\right)$$ (13)

$$\frac{{\text{dGt}}_{\text{A}}\left(\text{t}\right)}{\text{dt}}\;=\;-{\text{q}}_{1}{\text{Gt}}_{\text{A}}\left(\text{t}\right)\;+{\;\text{q}}_{2}(\text{G}\left(\text{t}\right)-{\text{G}}_{\text{b}})$$ (14)

Eq (13) is derived from Dalla Man *et al*. [22] and Eq (14) has been written following the same modeling approach. The first term in both equations models degradation, according to parameters p_2U_ and q_1_ for insulin and glucose, respectively. The second term describes the amount of plasma insulin (I) and plasma glucose (G) that moves to the interstitial compartment. In both cases, we consider only the amount exceeding the basal level, where I_b_ and G_b_ are the basal levels of plasma insulin and glucose, respectively, and p_2U_ and q_2_ are the transfer rates.

### Cellular (adipocyte) level model equations

The cellular level of the model, represented by the molecular specifications of the insulin signaling cascade in adipocytes, is described by Eqs (15) to (41), which were derived from Nyman *et al*. [26] and Brannmark *et al*. [35]. This part of the model represents the insulin signaling cascade in adipocytes, starting from the binding of interstitial insulin INS_A_ with the free insulin receptor on the adipocyte membrane (IR) and ending with the translocation of GLUT4 from the cytosol to the plasma membrane. Eqs from (15) to (39) are derived from Nyman *et al*. [26], while Eqs (40) and (41) are from Brannmark *et al*. [35]. The cellular model reconnects with the whole body model through Eq (9), where the increase of glucose mass in adipose tissue is modeled according to the amount of GLUT4 and GLUT1 [18,26].

The last five equations of the Nyman’s model introduced in [26] were not included here, as they describe regulative phenomena related to S6 and S6K which are not relevant for the scope of the present work. Therefore, Eqs (40) and (41), which model the dynamics of S6 and S6k, were taken from Brannmark *et al*. 2013 [35].

All the cellular equations are modeled through mass action kinetics as from [26] and [35]. The description and the value of all the parameters are provided in Table C in S1 File. Here we reported the list of model equations with a short description of the variables. We refer to the next section Model simulations for the description of the cellular dynamics and to [26] and [35] for any additional insight.

Eq (15) describes the dynamics of the free insulin receptor (IR) on the adipocyte membrane:

$$\frac{\text{dIR}\left(\text{t}\right)}{\text{dt}}\;=\;-{\text{k}}_{1\text{a}}\text{IR}\left(\text{t}\right){\text{INS}}_{\text{A}}\left(\text{t}\right)-{\;\text{k}}_{1\text{basal}}\text{IR}\left(\text{t}\right)+{\;\text{k}}_{1\text{g}}{\text{IR}}_{\text{YP}\left(\text{t}\right)}+{\;\text{k}}_{1\text{r}}\text{IRi}\left(\text{t}\right)$$ (15)

Eq (16) describes the dynamics of the phosphorylated insulin receptor (IR_YP). Phosphorylation can be insulin independent (parameter k_1basal_) and dependent (parameter k_1c_):

$$\frac{\text{dIR}\_\text{YP}(\text{t})}{\text{dt}}{\;=\;\text{k}}_{1\text{basal}}\text{IR}\left(\text{t}\right){\;+\;\text{k}}_{1\text{c}}\text{IRins}\left(\text{t}\right)-{\text{k}}_{1\text{d}}\text{IR}\_\text{YP}\left(\text{t}\;\right)-{\text{k}}_{1\text{g}}\text{IR}\_\text{YP}(\text{t})$$ (16)

Eq (17) represents the dynamics of the insulin receptor that is bound to insulin but not already phosphorylated (IRins):

$$\frac{\text{dIRins}(\text{t})}{\text{dt}}{\;=\;\text{k}}_{1\text{a}}\text{IR}\left(\text{t}\right){\text{INS}}_{\text{A}}(\text{t})\;-{\;\text{k}}_{1\text{c}}\text{IRins}\left(\text{t}\right)$$ (17)

Eq (18) represents the phosphorylated insulin receptor that has been endocytosed from the adipocyte (IRi_YP):

$$\frac{\text{dIRi}\_\text{YP}(\text{t})}{\text{dt}}{\;=\;\text{k}}_{1\text{d}}\text{IR}\_\text{YP}\left(\text{t}\right)-{\text{k}}_{1\text{f}}\text{IRi}\_\text{YP}\left(\text{t}\right)\text{X}\_\text{P}(\text{t})$$ (18)

Eq (19) represents the dynamics of the free internalized insulin receptor (IRi):

$$\frac{\text{dIRi}\left(\text{t}\right)}{\text{dt}}{\;=\;\text{k}}_{1\text{f}}\text{IRi}\_\text{YP}\left(\text{t}\right)\text{X}\_\text{P}(\text{t})-{\text{k}}_{1\text{r}}\text{IRi}\left(\text{t}\right)$$ (19)

Eqs from (20) to (23) describe the insulin receptor substrate 1 (IRS1) in its four phosphorylation forms. IRS1 is not phosphorylated, IRS1_YP is phosphorylated at the tyrosine site, IRS1_YP_S307P is phosphorylated at both the tyrosine and serine sites, IRS1_S307P is phosphorylated only at the serine site:

$$\begin{array}{ll} \frac{\text{dIRS}1\left(\text{t}\right)}{\text{dt}}= & {\text{k}}_{2\text{b}}\text{IRS}1\_\text{YP}\left(\text{t}\right){\;+\;\text{k}}_{2\text{g}}\text{IRS}1\_\text{S}307\text{P}\left(\text{t}\right)+\\ & -{\text{k}}_{2\text{a}}\text{IRS}1\left(\text{t}\right)\text{IR}\_\text{YP}(\text{t})-{\text{k}}_{2\text{basal}}\text{IRS}1(\text{t}) \end{array}$$ (20)

$$\begin{array}{ll} \frac{\text{dIRS}1\_\text{YP}\left(\text{t}\right)}{\text{dt}} & ={\text{k}}_{2\text{a}}\text{IRS}1\left(\text{t}\right)\text{IRi}\_\text{YP}\left(\text{t}\right)+{\text{k}}_{2\text{d}}\text{IRS}1\_\text{YP}\_\text{S}307\text{P}\left(\text{t}\right)+\\ & -{\text{k}}_{2\text{b}}\text{IRS}1\_\text{YP}\left(\text{t}\right)-{\text{k}}_{2\text{c}}\text{IRS}1\_\text{YP}\left(\text{t}\right)\text{mTORC}1\text{a}\left(\text{t}\right){\text{k}}_{\text{fb}} \end{array}$$ (21)

$$\begin{array}{ll} \frac{\text{dIRS}1\_\text{YP}\_\text{S}307\text{P}(\text{t})}{\text{dt}} & ={\text{k}}_{2\text{c}}\text{IRS}1\_{\text{YP}(\text{t})\text{mTORC}1\text{a}(\text{t})\text{k}}_{\text{fb}}\;+\\ & -{\text{k}}_{2\text{d}}\text{IRS}1\_\text{YP}\_\text{S}307\text{P}\left(\text{t}\right)-{\text{k}}_{2\text{f}}\text{IRS}1\_\text{YP}\_\text{S}307\text{P}\left(\text{t}\right) \end{array}$$ (22)

$$\frac{\text{dIRS}1\_\text{S}307\text{P}(\text{t})}{\text{dt}}{\;=\;\text{k}}_{2\text{basal}}{\text{IRS}1(\text{t})\;+\text{k}}_{2\text{f}}\text{IRS}1\_\text{YP}\_\text{S}307\text{P}\left(\text{t}\right)-{\text{k}}_{2\text{g}}\text{IRS}1\_\text{S}307\text{P}\left(\text{t}\right)$$ (23)

Eqs (24) and (25) represent the dynamics of the feedback protein X that, in its active form X_P, enhances the dephosphorilation of the internalized insulin receptor:

$$\frac{\text{dX}(\text{t})}{\text{dt}}{\;=\;\text{k}}_{3\text{b}}\text{X}\_\text{P}\left(\text{t}\right)-{\text{k}}_{3\text{a}}\text{X}\left(\text{t}\right)\text{IRS}1\_\text{YP}(\text{t})$$ (24)

$$\frac{\text{dX}\_\text{P}\left(\text{t}\right)}{\text{dt}}\;={\;\text{k}}_{3\text{a}}\text{X}\left(\text{t}\right)\text{IRS}1\_\text{YP}\left(\text{t}\right)-{\text{k}}_{3\text{b}}\text{X}\_\text{P}\left(\text{t}\right)$$ (25)

Eqs from (26) to (29) describe the four different forms of the protein kinase b: not phosphorylated (PKB), phosphorylated only at the threonine site (PKB_T308P), only at the serine site (PKB_S473P) and at both sites (PKB_T308P_S473P):

$$\frac{\text{dPKB}(\text{t})}{\text{dt}}{\;=\;\text{k}}_{4\text{b}}\text{PKB}\_\text{T}308\text{P}\left(\text{t}\right)+{\text{k}}_{4\text{h}}\text{PKB}\_\text{S}473\text{P}\left(\text{t}\;\right)-{\text{k}}_{4\text{a}}\text{PKB}\left(\text{t}\right)\text{IRS}1\_\text{YP}\left(\text{t}\right)$$ (26)

$$\begin{array}{ll} \frac{\text{dPKB}\_\text{T}308\text{P}(\text{t})\;}{\text{dt}} & ={\text{k}}_{4\text{a}}\text{PKB}\left(\text{t}\right)\text{IRS}1\_\text{YP}(\text{t})-{\text{k}}_{4\text{b}}\text{PKB}\_\text{T}308\text{P}\left(\text{t}\right)+\\ & -{\text{k}}_{4\text{c}}\text{PKB}\_\text{T}308\text{P}\left(\text{t}\right)\text{mTORC}2\text{a}\left(\text{t}\;\right) \end{array}$$ (27)

$$\begin{array}{ll} \frac{\text{dPKB}\_\text{S}473\text{P}(\text{t})}{\text{dt}} & ={\text{k}}_{4\text{f}}\text{PKB}\_\text{T}308\text{P}\_\text{S}473\text{P}\left(\text{t}\right)-{\text{k}}_{4\text{e}}\text{PKB}\_\text{S}473\text{P}\left(\text{t}\right)\text{IRS}1\_\text{YP}\_\text{S}307\text{P}(\text{t})+\\ & -{\text{k}}_{4\text{h}}\text{PKB}\_\text{S}473\text{P}\left(\text{t}\right) \end{array}$$ (28)

$$\begin{array}{ll} \frac{\text{dPKB}\_\text{T}308\text{P}\_\text{S}473\text{P}(\text{t})}{\text{dt}} & ={\text{k}}_{4\text{c}}\text{PKB}\_\text{T}308\text{P}\left(\text{t}\right)\text{mTORC}2\text{a}\left(\text{t}\;\right)+\\ & {\;+\;\text{k}}_{4\text{e}}\text{PKB}\_\text{S}473\text{P}\left(\text{t}\right)\text{IRS}1\_\text{YP}\_\text{S}307\text{P}(\text{t})-{\text{k}}_{4\text{f}}\text{PKB}\_\text{T}308\text{P}\_\text{S}473\text{P}\left(\text{t}\right) \end{array}$$ (29)

Eqs (30) and (31) describe the protein complex mTORC1 (mammalian target of rapamycin mTOR in complex with raptor) in its inactive (mTORC1) and active (mTORC1a) forms:

$$\begin{array}{ll} \frac{\text{dmTORC}1\text{a}(\text{t})\;\;}{\text{dt}} & {=\;\text{k}}_{5\text{b}}\text{mTORC}1\text{a}(\text{t})-{\text{mTORC}1(\text{t})(\text{k}}_{5\text{a}1}\text{PKB}\_\text{T}308\text{P}\_\text{S}473\text{P}\left(\text{t}\right)+\\ & +{\text{k}}_{5\text{a}2}\text{PKB}\_\text{T}308\text{P}\left(\text{t}\right)\text{)} \end{array}$$ (30)

$$\begin{array}{ll} \frac{\text{dmTORC}1\text{a}(\text{t})\;}{\text{dt}}= & {\text{mTORC}1(\text{t})(\text{k}}_{5\text{a}1}\text{PKB}\_\text{T}308\text{P}\_\text{S}473\text{P}\left(\text{t}\right)+{\text{k}}_{5\text{a}2}\text{PKB}\_\text{T}308\text{P}\left(\text{t}\right))+\\ & -{\text{k}}_{5\text{b}}\text{mTORC}1\text{a}(\text{t}) \end{array}$$ (31)

Eqs (32) and (33) represent the protein complex mTORC2 (mammalian target of rapamycin mTOR in complex with rictor) in its inactive (mTORC2) and active (mTORC2a) forms:

$$\frac{\text{dmTORC}2(\text{t})}{\text{dt}}\;=\;-{\text{k}}_{5\text{c}}\text{mTORC}2(\text{t})\text{IRi}\_\text{YP}(\text{t})+{\text{k}}_{5\text{d}}\text{mTORC}2\text{a}(\text{t})$$ (32)

$$\frac{\text{dmTORC}2\text{a}(\text{t})}{\text{dt}}{\;=\;\text{k}}_{5\text{c}}\text{mTORC}2(\text{t})\text{IRi}\_\text{YP}(\text{t})-{\text{k}}_{5\text{d}}\text{mTORC}2\text{a}(\text{t})$$ (33)

Eqs (34) and (35) describe AS160, the substrate of PKB, and its phosphorylated form AS160_T642P:

$$\begin{array}{l} \begin{array}{ll} \frac{\text{dAS}160(\text{t})}{\text{dt}}\; & ={\text{k}}_{6\text{b}}\text{AS}160\_\text{T}642\text{P}(\text{t})-{\text{AS}160(\text{t})(\text{k}}_{6\text{a}1}\text{PKB}\_\text{T}308\text{P}\_\text{S}473\text{P}\left(\text{t}\right)+\\ & +{\text{k}}_{6\text{a}2}\text{PKB}\_\text{S}473\text{P}(\text{t})) \end{array}\\ \; \end{array}$$ (34)

$$\begin{array}{ll} \frac{\text{dAS}160\_\text{T}642\text{P}(\text{t})}{\text{dt}}\; & ={\text{AS}160(\text{t})(\text{k}}_{6\text{a}1}\text{PKB}\_\text{T}308\text{P}\_\text{S}473\text{P}\left(\text{t}\right)+{\text{k}}_{6\text{a}2}\text{PKB}\_\text{S}473\text{P}(\text{t}))+\\ & -{\text{k}}_{6\text{b}}\text{AS}160\_\text{T}642\text{P}(\text{t}) \end{array}$$ (35)

Eqs (36) and (37) represent glucose transporter 4 inside the adipocyte cytosol (GLUT4) and on the cell membrane (GLUT4m):

$$\frac{\text{dmGLUT}4\text{m}(\text{t})}{\text{dt}}{\;=\;\text{k}}_{7\text{a}}\text{GLUT}4(\text{t})\;\text{AS}160\_\text{T}642\text{P}(\text{t})-{\text{k}}_{7\text{b}}\text{GLUT}4\text{m}(\text{t})$$ (36)

$$\frac{\text{dmGLUT}4(\text{t})}{\text{dt}}\;=\;-{\text{k}}_{7\text{a}}\text{GLUT}4(\text{t})\;\text{AS}160\_\text{T}642\text{P}(\text{t})+{\text{k}}_{7\text{b}}\text{GLUT}4\text{m}(\text{t})$$ (37)

Eqs (38) and (39) describe the dynamics of the S6 kinase (S6K) and its phosphorylated form S6K_T389P:

$$\frac{\text{dS}6\text{K}(\text{t})}{\text{dt}}{\;=\;\text{k}}_{9\text{b}}\text{S}6\text{K}\_\text{T}389\text{P}(\text{t})(\text{t})-{\text{k}}_{9\text{a}}\text{S}6\text{K}(\text{t})\text{mTORC}1\text{a}(\text{t})$$ (38)

$$\frac{\text{dS}6\text{K}\_\text{T}389\text{P}(\text{t})}{\text{dt}}{\;=\;\text{k}}_{9\text{a}}\text{S}6\text{K}(\text{t})\text{mTORC}1\text{a}(\text{t})-{\text{k}}_{9\text{b}}\text{S}6\text{K}\_\text{T}389\text{P}(\text{t})$$ (39)

Eqs (40) and (41) represent the ribosomal protein S6 and its phosphorylated form S6_S235_S236P.

$$\frac{\;\text{dS}6(\text{t})}{\text{dt}}{\;=\;\text{k}}_{9\text{b}2}\text{S}6\_\text{S}235\_\text{S}236\text{P}(\text{t})-{\text{k}}_{9\text{f}2}\text{S}6(\text{t})\text{S}6\text{K}\_\text{T}389\text{P}(\text{t})$$ (40)

$$\frac{\text{dS}6\_\text{S}235\_\text{S}236\text{P}(\text{t})}{\text{dt}}{\;=\;\text{k}}_{9\text{f}2}\text{S}6(\text{t})\text{S}6\text{K}\_\text{T}389\text{P}(\text{t})-{\text{k}}_{9\text{b}2}\text{S}6\_\text{S}235\_\text{S}236\text{P}(\text{t})$$ (41)

#### Model simulations

**Fig 3:**
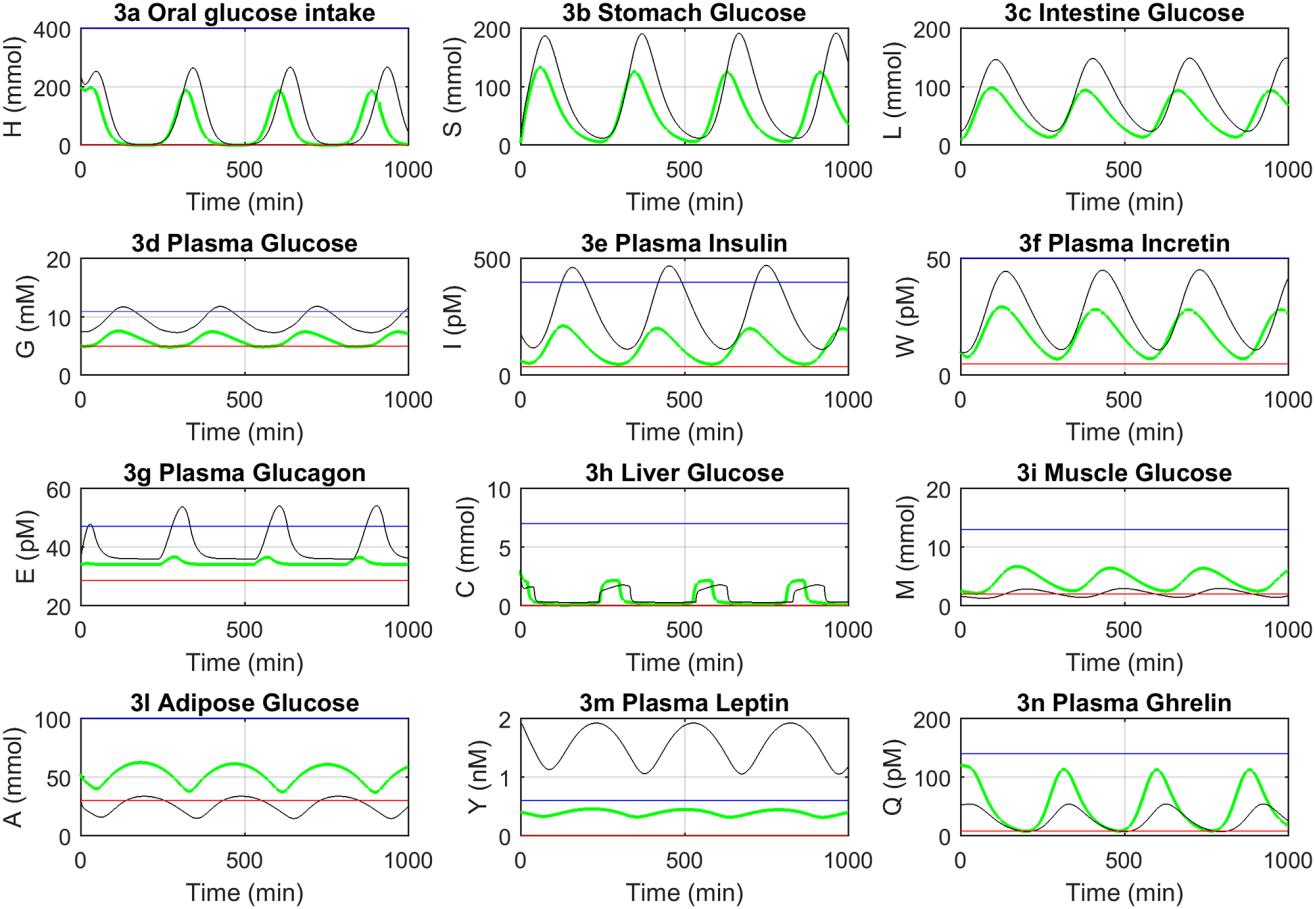
Model dynamics at the whole body level. Each plot represents one variable dynamics. The normal glucose regulation (NGR) and T2DM conditions are shown in green and black, respectively. The red and blue lines delimit the physiological lower and upper ranges of variables (see also Table B in S1 File).

The model has been simulated for 1000 minutes (three consecutive meals) starting from an initial condition representing the fasting state (t = 0). The model initial values and parameter estimates have been computed as introduced in Materials and Methods and they are reported in Tables A and C in S1 File, respectively. Fig 3 shows the dynamics of each model variable at the whole body level. The green and black lines represent the NGR and T2DM conditions, respectively. The physiological upper and lower ranges for each variable are shown in blue (higher line, HL) and red (lower line, LL) straight lines, according to the available estimates from the literature (see also Table B in S1 File). The model exhibited an oscillatory behavior in both the NGR and T2DM conditions through alternate parameter sets that accounted for the reciprocal interaction among the constituting variables in the two conditions.

In the NGR condition, the system started at fasting by simulating an oral glucose intake (Fig 3a) and, after a time lag accounting for the transit time among compartments, was followed by subsequent transitions through the stomach (Fig 3b) to the intestine (Fig 3c). The glucose absorption from the intestine to the bloodstream was characterized by a further time lag (Fig 3d) and it triggered the increase in circulating insulin levels (Fig 3e). We modeled the glucose transit through the intestine as a stimulus for the secretion of incretins (Fig 3f), which ultimately resulted in an amplification of the endogenous insulin secretion. The secretion of glucagon (Fig 3g) was modeled as being inhibited by high glucose and insulin concentrations, and increased in case of markedly low plasma glucose levels, thus stimulating the endogenous glucose output from the liver (Fig 3h). The glucose uptake by the muscle and adipose tissues was favored in case of high insulin concentrations, thus resulting in a net increase of the glucose mass in these tissues (Fig 3i and 3l).

**Fig 4:**
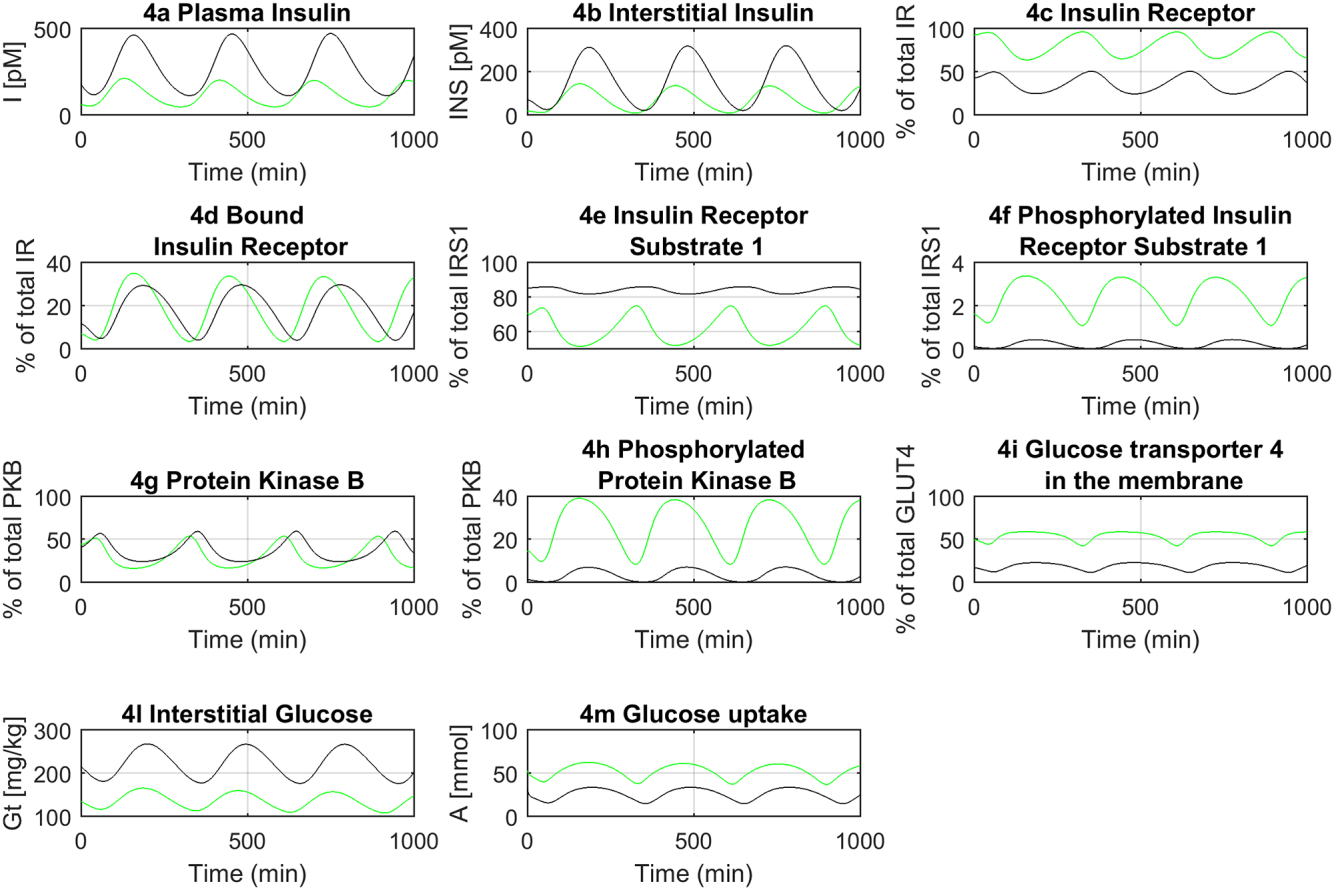
Model dynamics of the insulin signaling in adipocytes at the cellular level (only a subset of key variables is represented). The normal glucose regulation (NGR) and T2DM conditions are shown in green and black, respectively.

The whole-body model has been linked to the adipocyte cellular level through the interstitial fluid surrounding the individual cells. Here we assumed the interstitial fluid being in direct communication with the plasma. Therefore, the two layers of abstraction (*i*.*e*. the whole-body and the adipocyte levels) were bound through the interstitial insulin and glucose (INS_A_ and Gt_A_, Fig 2) in a conceptual framework closely mirroring the physiology of insulin signaling. The interstitial fluid, rather than the plasma, is surrounding the cells targeted by insulin, it flows in our model from the plasma to the interstitial space with a time shift (Fig 4), and then binds its membrane receptors, triggering the downstream cascade of signaling events. The inactive and un-phosphorylated components of the cascade, such as the unbound insulin receptor (IR) or the un-phosphorylated protein kinase B (PKB), proportionally decreased at incremental concentrations of interstitial insulin (Fig 4c and 4g). The opposite occurred to the active and phosphorylated components, such as the bound insulin receptor (IRins) or the phosphorylated insulin receptor substrate 1 (IRS1_YP), which increased at higher levels of interstitial insulin (Fig 4d and 4f), thus allowing the activation of the insulin signaling cascade and eventually leading to the translocation of glucose transporter type 4 (GLUT4) to the cell membrane. The output of the cellular layer was linked to the whole-body output through the amount of GLUT4 on the adipocyte membrane, which directly affected the glucose uptake by the adipose tissue (Fig 4m). This latter variable was in fact shared between the two layers together with interstitial insulin and glucose.

In the T2DM condition, the initial value of several model variables, such as plasma glucose and insulin, and some of the parameters, were modified, as described in Materials and Methods and reported in Tables A and C in S1 File, in order to simulate a T2DM condition of a drug-naïve individual patient. It can be observed that, as compared to NGR, glucose dynamics (Fig 3a to 3d) showed a broader range in the T2DM condition, as well as that of insulin, incretin and glucagon (Fig 3e to 3g). The total mass and the output rate of hepatic glucose production were reduced (Fig 3h), as well as glucose uptake by muscle and adipose tissues (Fig 3i and 3l). According to T2DM pathophysiology, insulin effectiveness was reduced in the T2DM condition. Despite higher absolute insulin levels, the glucose uptake mechanism was impaired in the muscles, adipocytes and liver. The impaired insulin signaling cascade affects glucose uptake, resulting in decreased glucose uptake by the adipose tissue and leptin secretion. An increased leptin concentration was observed in the T2DM experimental condition (Fig 3m), as the individual fat mass was set to an increased level, according to Grasman *et al*. [40] (see Table C in S1 File, Fat parameter).

The estimates of some model parameters, including the number of IRs and GLUT4, and the positive feedback from mTORC1, have been modified, according to Nyman *et al*. [26], to simulate the T2DM condition at the cellular level (see Materials and methods and S1). The diminished total number of IRs led to reduced IR binding and phosphorylation (Fig 4c, 4d and 4f). Similarly, the reduced total concentration of GLUT4 affected the amount of GLUT4 eventually docking to the adipocyte membrane (Fig 4i). The reduced positive feedback from mTORC1 had a more general regulatory effect on all the components of the insulin signaling cascade starting from a lower level of IRS1_YP, where the protein complex mTORC1 acts directly (Fig 4f). All changes applied in the previously described model parameters harmonically worked together to reduce the amount of GLUT4, eventually leading to reduced glucose uptake by the adipocytes (Fig 4m).

**Fig 5:**
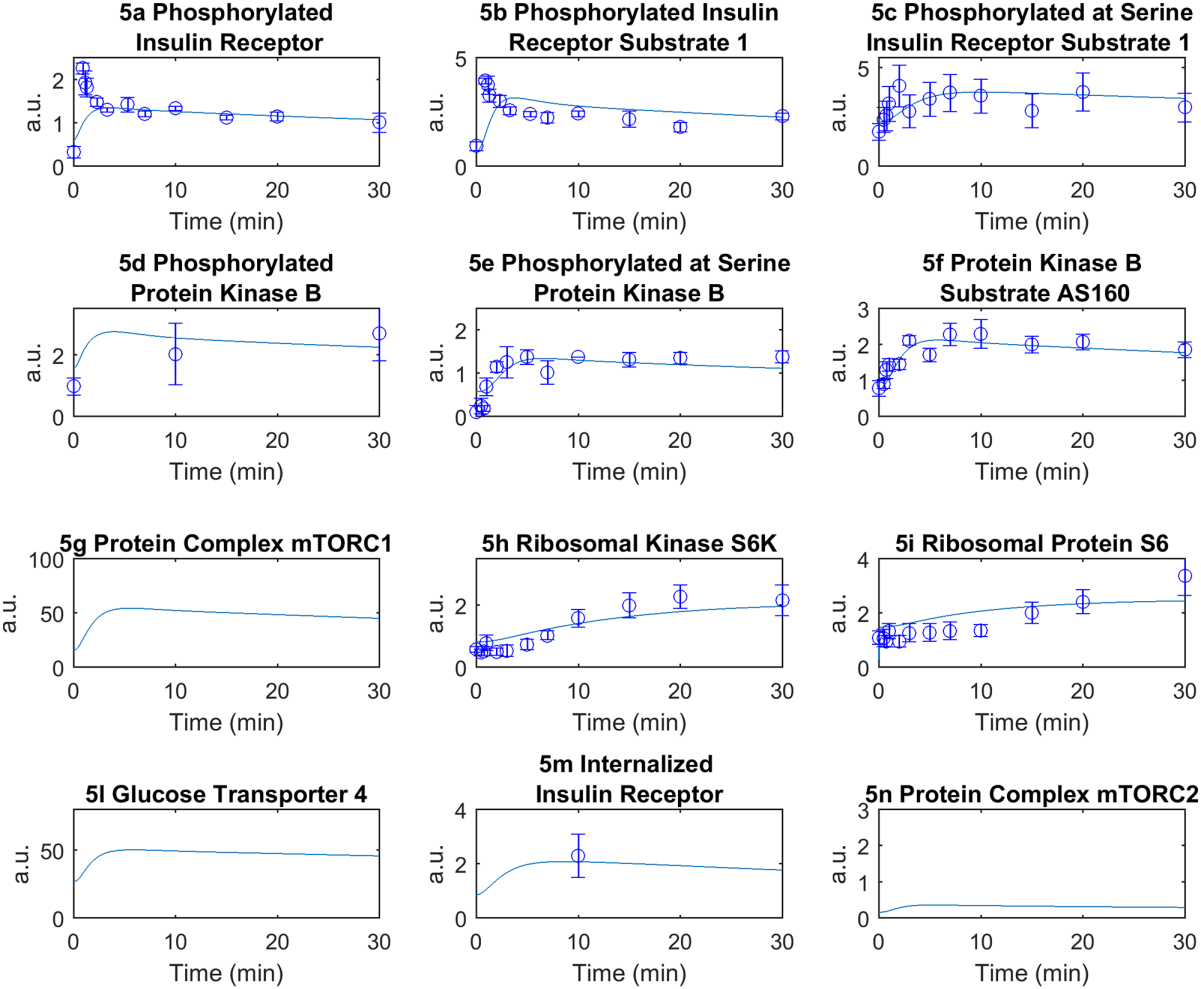
Model simulation and data fitting, normal glucose regulation (NGR) condition. Each plot represents the corresponding time courses for the indicated insulin signaling intermediaries. The experimental data are taken from Nyman *et al*. [26] and are represented with circles and error bars (a.u. indicates arbitrary units). The time course represents the model simulation.

**Fig 6:**
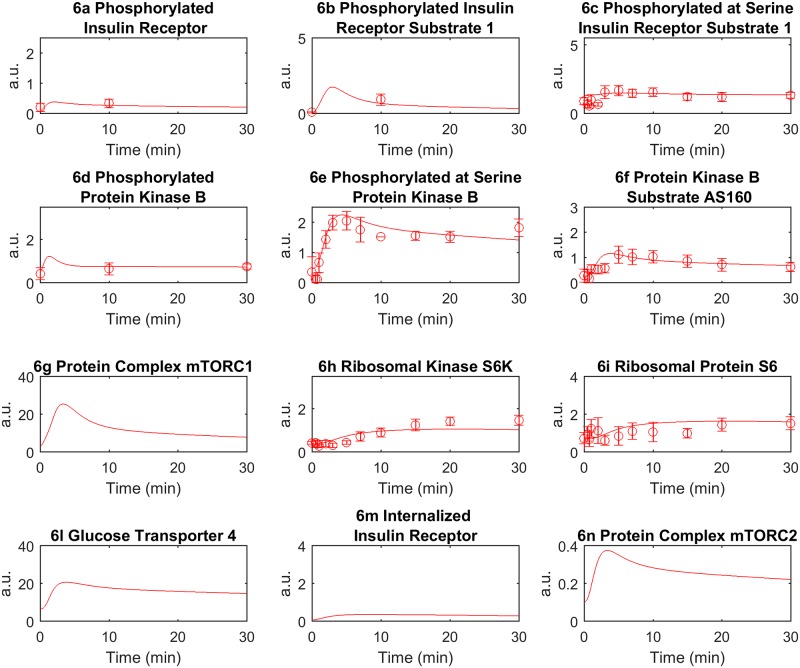
Model simulation and data fitting, T2DM condition. Each plot represents the corresponding time courses for the indicated insulin signaling intermediaries. The experimental data are taken from Nyman *et al*. [26] and are represented with circles and error bars (a.u. indicates arbitrary units). The time course represents the model simulation.

In order to test the consistency of the model with physiology, we fitted the same experimental values employed by Nyman *et al*. [26] at the cellular level in both the NGR and T2DM conditions, as shown in Figs 5 and 6, respectively (see also Materials and methods). According to the physiology governing the reciprocal interactions among leptin, insulin and ghrelin, we were also able to roughly reproduce the fluctuations of circulating ghrelin levels, which are typically characterized in humans by a marked reduction after meal ingestion and by a rebound to baseline before the next meal [46,47]. Of note, although we were unable to capture, by design, the circadian fluctuations of ghrelin (which usually increases after an overnight fast) and other hormones, our model successfully reproduced the physiologic dynamics of ghrelin by inversely paralleling those of insulin (Fig 3e and 3n). Conversely, in accordance with the role of leptin as prototypical regulator of energy homeostasis and its dependence from adipose tissue mass, the dynamics of leptin returned by the model in both T2DM and NGR conditions showed much dampened fluctuations, as compared to those of ghrelin and insulin (Fig 3m).

## Discussion

The aim of this work was to introduce a closed-loop multi-level model of human glucose homeostasis, describing, in a hierarchical multi-scale system architecture, the contribution of its main determinants to the NGR and T2DM conditions, at both the whole-body and cellular (adipocyte) levels. The modeling strategy merged two different physiological levels, the organ and the cellular one, to ground the basis for the inclusion of the other main players in glucose homeostasis (such as muscle, pancreatic and liver cells) as additional compartments of the cellular level.

The model was successfully tested *in silico* for NGR and T2DM conditions, which have been described through alternate initial conditions and parameter estimates (see Materials and methods and Tables A and C in S1 File). The output of the model (whole-body glucose needs) coincided with the input (oral glucose intake) in a closed-loop fashion, which allowed to perpetually simulate whole body dynamics, according to a self-feeding system. Whenever possible, model equations have been directly derived from the literature and then adapted in order to work together through the identification of suitable initial states and parameter estimates (see Materials and methods and S1 for details). The shape of model equations has been left unchanged in most instances for consistency with those introduced in the original works. This approach provides the following advantages: (i) it allows to rely on very established model equations, that have been extensively analyzed in the literature to describe the physiology of interest; (ii) it does not require to estimate *de novo* several parameters (as it would be required in case of equation reshaping), which can rather be derived directly from the literature; (iii) it allows a fairer comparison of the results herein presented with those already discussed in the literature. However, this modeling strategy has the disadvantage that some equations could look different, even if they model similar processes. For example, saturation has been modeled either by considering Michaelis-Menten terms as in Eq (9) or by relying on more abstracted exponential terms as in Eq (11). We remark that this discrepancy does not affect the reliability of model simulations because the set of ODEs has been parameterized to have all model variables within their physiological ranges during the simulations. Therefore, model equations are computed in the same conditions considered in the papers where they have been originally introduced.

The closed-loop system, which has the unique advantage to simulate experimental conditions for long time windows without external intervention, was achieved through the inclusion of the hunger signal, here intended as the amount of glucose needed from the body. Hunger description was possible through leptin and ghrelin, which work as complementary molecules to regulate food intake and energy balance in close concert to insulin [46,48]. Ghrelin is a fast-acting hormone secreted when the stomach is empty [49] and it stimulates food intake. Leptin concentration depends on fat mass [50] and acts on the long term, without major changes within hours or days (Fig 3m), as a “satiety” signal to the brain [51,52]. Of note, although T2DM individuals are often characterized by increased leptin concentrations due to increased fat mass, a mechanism of leptin resistance occurs, thus making them relatively insensitive to leptin [53].

The dual role of the liver as both glucose storage and production site is a novel feature of our model, since the inclusion of both the regulative effects of insulin and glucagon has never been considered in previous models [22,54]. Glucagon is a hormone secreted from pancreatic alpha cells at low glycemic conditions, which signals the liver to release glucose from glycogen storages, thus maintaining the euglycaemic state at fasting [55]. Sub-diabetic hyperglycemic states and overt T2DM are often characterized by high fasting plasma glucose levels, due to an excessive glucose output from the liver, as a consequence of liver insensitivity to insulin or abnormally high glucagon [56].

Although a number of models have been previously developed by including the dynamics of glucagon [10,29,54], incretins [9,11,34], leptin [57] and ghrelin [9], we have considered all these factors together for the first time. The inclusion of these components, as well as the distinction of adipose and muscle glucose uptake, previously reported together [29], provided a better description of the reciprocal connections existing between the whole-body and the cellular levels and allowed us to physically “close the loop” among different layers of abstraction.

Of note, we have observed that, as compared to the NGR state, the T2DM condition in the model displayed slower dynamics. Although the reciprocal changes in ghrelin (decrease) and insulin (increase) would drive the need for increased glucose intake, the resulting emptying rate of the stomach is slower, as well as the glucose uptake from adipose tissue and muscles, thus leading to a delayed dynamics in the whole system. These dynamics may be interpreted in light of the constituting principle regulating the model, which takes into account the supposed individual energy requirements. Therefore, our model correctly reflects the physiological response of the organism to maintain the glucose homeostasis within a physiological range in case of an imbalance between energy requirements and energy intake (*i*.*e*. overfeeding) often observed in patients with T2DM.

Our study has however a number of limitations that should be addressed. We do acknowledge the peculiar use of the term “closed-loop” herein employed, which is usually linked, in the dictionary of the diabetes community, to the so-called “artificial pancreas”, *i*.*e*. a system that is able to automatically predict *in silico* the adjustments of external insulin delivery needed to keep the circulating plasma glucose within a narrow range of physiologic fluctuations. In the context of the present study, we were not limiting the term “closed-loop” to a sort of “artificial beta cell”. We rather sought to describe the governance of glucose homeostasis by applying a broader, holistic approach. It should however be pointed out that our modeling effort lacks of a comprehensive description of the hormonal networks and molecular cascades occurring at each organ and tissue involved in the regulation of glucose homeostasis. The rationale surrounding our choice of focusing on the intracellular molecular cascade occurring within the adipocytes was not solely dictated by the relevance of the adipose tissue as an “endocrine organ” influencing systemic energy balance and glucose homeostasis [24,25], but also by the availability of detailed mathematical specifications of the insulin signaling cascade recently provided by the group of Nyman *et al*. [26].

Furthermore, it could be argued that a whole-body description similar to the one herein proposed can be defined without the addition of a cellular level. However, the integration of the two levels of abstraction within a single model allows the detailed observation of the reciprocal effects of changes occurring between the constituents of the cellular and whole-body levels. Thus, the hierarchical modeling strategy allows to simply zoom in on specific areas of interest (in our case, the adipocytes) in order to investigate regulatory effects that may occur between the two levels of abstraction. For instance, the action of a molecule on a receptor could be easily included in the specifications of the cellular level, and its effects at the whole-body level could be observed, thus allowing the identification of the changes in the organ variables caused by variations of the cellular ones. Therefore, the addition of a level in a hierarchical modeling structure does not imply that the rest of the model would not stand by itself, but it is rather there to allow the consideration of other (*e*.*g*. molecular) effects within a wider framework.

Nevertheless, despite its intrinsic limitations, our hierarchical modeling effort demonstrated sufficient robustness to provide a fair description of the core determinants of glucose homeostasis at both cellular and systemic scales. As such, given its unique modular architecture, the multi-level model herein tested constitutes a promising backbone to annex further layers of detail.

The model describes the normal glucose regulation and the diabetic states through alternate parameter sets (see Table C in S1 File), where the constraints for those parameters have been mostly derived from the available literature as detailed in Materials and Methods. This approach is blind to distinguish between primary changes that drive disease progression and secondary changes that are consequences or adaptations to the primary ones. Other independent research groups, such as Topp *et al*. [58] and De Gaetano *et al*. [59], have addressed, at variance with our approach, the argument of glucose homeostasis regulation from a pathogenesis modeling standpoint. Topp *et al*. [58] for instance, have specifically investigated the link between beta-cell mass and beta-cell function by a set of nonlinear ODEs, where the glucose and insulin dynamics are designed to be fast relative to beta-cell mass dynamics. On the contrary, when we compared with our model the estimates obtained in the T2DM condition to those from the NGR state, it was impracticable to distinguish whether the reduction in the secretory capacity (parameter b_4_, see Table C in S1 File) reflected reduced beta-cell mass or function, or both. Of note, even in the absence of a pre-specified and detailed mathematical description of the beta-cell function machinery, the value of this parameter was not imposed in advance, but it rather represents a consequence of the steady state analysis applied to the equation modeling insulin concentration in plasma (Eq (4)), according to what introduced by Toghaw *et al*. [9]. As previously anticipated, and similarly to the case of other key determinants of glucose homeostasis, this encouraging result may be considered as a rough indicator of the goodness of the model, which leaves the beta-cell component open to further hierarchical refinements.

In conclusion, coupling the cellular level model with a closed-loop whole body model allowed us to evaluate the behavior of adipocytes not only during one meal but in a perpetual fashion. The simulation of the system over such a long time frame highlighted the reciprocal reactions occurring between the two levels of abstraction, *i*.*e*. the organ and the cellular levels. The model provided a seamless dynamic description of the molecular mechanisms downstream the insulin receptor in adipocytes, thus demonstrating the usefulness of a multi-level approach to the modeling of glucose homeostasis at both cellular and systemic scales. As for the potential applications, the herein proposed model architecture is intrinsically open to integrate supplementary layers of specifications for individual components. As such, more detailed and advanced versions of the present model could potentially be applied to investigate *in silico* the effect of specific drugs pointing to one or more of the model constituents or to identify currently unmet molecular targets amenable to pharmacological intervention.

## Materials and methods

### Mathematical model and computational framework

The multi-level model has been defined as a set of ordinary differential equations (ODEs) [60] implemented in Matlab 2015b. The model has been numerically simulated by means of the state of the art ODE solver ode15s. A state of the art sensitivity analysis of estimated parameters has been also computed to assess parameter identifiability (see S1 for details).

### Initial values and parameter estimates

Initial values of all the model variables are listed in Table A in S1 File, for both NGR and T2DM conditions. Simulations start by assuming a morning fasting state, the initial values of model variables have been derived from the literature or obtained by nonlinear optimization constrained to the variability of physiological ranges. Initial values have been selected to find the best balance between human physiology and reliability of the model dynamics in order to avoid discontinuities or states with unrealistic variable values, such as negative values or values outside the physiological ranges listed in Table B in S1 File. For what concerns the cellular model, the variability range of model variables used during the optimization has been inferred *in silico*: Nyman’s model has been simulated according to [26] and the minimum and maximum values reached by each variable have been considered.

Parameter estimates have been determined following different methodologies. When a reference from the literature was available, parameter estimates were directly taken from the literature or derived by following the same procedure indicated in the reference paper. Parameters b_4_, b_5_, b_12_, b_13_, b_17_, b_27_, c and c_3_ have been estimated through steady state analysis, that is, by imposing a steady state condition at time 0 on the corresponding equations as indicated in the literature. In the other cases, parameters have been estimated by nonlinear optimization constrained to obtain values within the physiological ranges discussed in the literature. The remaining parameters b_9_, r, k_gluc_, q_1_ and q_2_ were derived by unconstrained optimization to obtain a model dynamics consistent with physiology, that is, a dynamics without discontinuities or unrealistic variable values (*i*.*e*., negative values or values outside physiological ranges). For what concerns the cellular model, parameters are all taken from [26] and [35], except for k_1a_, which has been re-estimated within the same optimization range of [26] in order to have the minimum value of the IRins dynamics of Eq (17) consistent with that reported by Lodish *et al*. [61] and to preserve the model fits introduced by Nyman *et al*. [26] and provided in Figs 5 and 6. We refer to Table C in S1 File for any further insight on the employed estimation procedures and for a complete list of all parameter estimates computed for the NGR and T2DM conditions.

### Experimental data

In order to be consistent with previous results, the cellular level of the model describing the insulin signaling in adipocytes has been fitted by considering the same experimental data used in the paper of Nyman *et al*. [26] (Figs 5 and 6).

## Supporting information

S1 File Supplementary description of the mathematical model. (DOCX)

S2 File Charts providing the sensitivity analysis of estimated parameters for each variable of the whole body model in the NGR condition (see also S1 File). (PDF)

S3 File Charts providing the sensitivity analysis of estimated parameters for each variable of the whole body model in the T2DM condition (see also S1 File). (PDF)
