# Supplementary material for: A closed-loop multi-level model of glucose homeostasis
Source: PLoS One. 2018 Feb 8;13(2):e0190627. doi: 10.1371/journal.pone.0190627 (PMC5805234; doi:10.1371/journal.pone.0190627)
Supplement: S1 File — (DOCX) [file pone.0190627.s001.docx]

**A closed-loop multi-level model of glucose homeostasis**

**Supplementary File S1**

Cansu Uluseker^[[1]](#footnote-1)^, Giulia Simoni^1^, Luca Marchetti^1^, Marco Dauriz^2^, Alice Matone^1^ and Corrado Priami^1,3,*^

**Contents**

1. **Sensitivity analysis**
2. **Supplementary tables:**
   1. **Table A.** **Initial values of model variables in agreement with a morning fasting state.**
   2. **Table B. Physiological ranges of the whole body variables in the normal glucose regulation (NGR) condition.**
   3. **Table C. Parameter estimates of the model in the normal glucose regulation (NGR) and T2DM conditions.**
3. **Supplementary figures:**
   1. **Figure A. Heatmap resuming the results of the sensitivity analysis by considering the parameter estimates computed for the normal glucose regulation (NGR) condition.**
   2. **Figure B. Heatmap resuming the results of the sensitivity analysis by considering the parameter estimates computed for the T2DM condition.**
4. **Background physiology:**
   1. **Glucose physiology at the whole-body level**
   2. **Insulin signaling at the cellular (adipocyte) level**
5. **References**

**1. Sensitivity analysis**

A state of the art sensitivity analysis has been computed for each variable of the whole body model to assess the effect on the model dynamics of each estimated parameter. During the analysis, we did not consider the cellular model because its equations and parameters have been all taken from [1], [2] and [3] and therefore we refer to such references for any additional insight related to this layer of the model. Following the same approach, we did not consider the parameters of the whole body model that have been taken from literature without the need of being estimated. Conversely, the effect of each estimated parameter has been analyzed by considering their estimate both in the NGR condition and in T2DM, according to the values reported in Table C in S1 File.

For computing the analysis, each parameter estimate has been perturbed by multiplying its value by a parameter $k$ varying in the range 0.5 - 2 (from halved to doubled parameter estimate). For each value of the $k$ parameter, we ran a model simulation and then we measured the impact on the system dynamics of each model variable in terms of AUC ratio:

${AUC}_{ratio}^{k}={{AUC}_{perturbed}^{k}}/{{AUC}_{original}}$,

where AUC indicates the area under the curve provided by the corresponding simulated behavior in time. This allowed us to compute the set of charts included in S2 and S3 Files, which show the variation of the AUC ratio of each model variable for each value of $k$ in the two modeled conditions (NGR and T2DM). To improve the readability of results, only the parameters with

$$\left( \max_{k} {AUC}_{ratio}^{k}-\min_{k} {AUC}_{ratio}^{k} \right)\geq15\%$$

have been included in the plots.

Interestingly, the computed AUC ratios exhibit close-to-linear differentiable patterns with respect to the value of $k$, when the parameter is close to 1. This could indicate that model equations have the right structure to allow parameter identifiability. Only few parameters exhibit not-differentiable points in the behavior of their AUC ratio, but this is due to the thresholds employed in the model, which are all justified by literature (see main text and previous sections). Moreover, all the estimated parameters affect the dynamics of at least one model variable, which is another key requirement for parameter identifiability. The only parameter that showed to have a negligible impact on the dynamics of all model variables in the NGR condition is $c_{2}$, but this does not constitute a problem because it has been estimated in the T2DM condition, where an impact on the system dynamics can be appreciated. This can be easily checked by looking at the two heatmaps provided in Figure A and B in S1 File, which further resume the results of the sensitivity analysis. The two figures, one for the NGR condition and one for T2DM, depict in one single image the differences between the two AUC ratios computed with the maximum and the minimum considered value of $k$

$${AUC}_{ratio}^{k=2}-{AUC}_{ratio}^{k=0.5}$$

for each model variable and for each estimated parameter (differences lower than 0.15 have been not displayed). This provides a general view of the impact of each estimated parameter in both the NGR and T2DM conditions.

**2. Supplementary tables**

| Table A. **Initial values of model variables in agreement with a morning fasting state. For each variable two initial values are provided, one for the normal glucose regulation (NGR) condition and one for T2DM. Values have been computed according to the estimation procedure described in the text** | | | | | |
| --- | --- | --- | --- | --- | --- |
|  | | | | | |
| Variable description | Symbol | NGR | **Estimation Procedure** | T2DM | **Estimation Procedure** |
| Fasting stomach glucose | S(0) | 4 mmol | optimized around 0 to avoid discontinuities in the dynamics | 14 mmol | optimized around 0 to avoid discontinuities in the dynamics |
| Fasting intestine glucose | L(0) | 14 mmol | optimized around 0 to avoid discontinuities in the dynamics | 25 mmol | optimized around 0 to avoid discontinuities in the dynamics |
| Fasting plasma glucose | G(0) | 5 mM | taken from [4] | 7.5 mM | optimized in the range 8.05 ± 1.82 mM from [5] |
| Fasting plasma insulin | I(0) | 60 pM | taken from [4] | 180 pM | optimized in the range 171 ± 74 pM from [5] |
| Fasting plasma incretin | W(0) | 10 pM | optimized in the range 10 - 19.4 pM from [6] | 10 pM | as in NGR |
| Fasting plasma glucagon | E(0) | 34 pM | optimized in the range 37.86 ± 9.18 pM from [7] | 36 pM | optimized in the range 42.19± 10.67 pM from [7] |
| Fasting liver glucose | C(0) | 3 mmol | optimized in the range 3 - 5 mmol from [8] | 3 mmol | as in NGR |
| Fasting muscle glucose | M(0) | 2.5 mmol | optimized around the value from [8] ± 5% | 2.5 mmol | as in NGR |
| Fasting intra-adipocitary glucose | A(0) | 53.19 mmol | optimized in the range 30 - 75 mmol from [8] | 32 mmol | unconstrained optimization |
| Fasting plasma leptin | Y(0) | 0.4 nM | optimized in the range 0.11 - 1.75 nM [9] | 1.93 nM | unconstrained optimization |
| Fasting plasma ghrelin | Q(0) | 120 pM | optimized in the range 72.8 - 146.1 pM from [10] | 53 pM | optimized in the range 50.6–78.9 pM from [10] |
| Glucose intake | H(0) | 200 mmol | minimum daily glucose need taken from [11] divided by the number of meals | 240 mmol | minimum daily glucose need taken from [11] divided by the number of meals |
| Interstitial Insulin | $\mathrm{IN}S_{A}$(0) | 20 pM | unconstrained optimization | 70 pM | derived to preserve the same ratio I(0)/$\mathrm{IN}S_{A}$ (0) of the NGR condition |
| Interstitial Glucose | $\mathrm{Gt}_{A}$ (0) | 135 mg/kg | taken from [12] | 216 mg/kg | derived to preserve the same ratio G(0)/$\mathrm{Gt}_{A}$ (0) of the NGR condition |
| Free insulin receptor | IR(0) | 92.9271 | optimized in the range 1.3298 - 99.8724 from [1] | 43.4601 | optimized in the range 0.7319 - 54.9280 from [1] |
| Phosphorylated insulin receptor | IR_YP(0) | 0.0047 | optimized in the range 0.0019 - 0.0438 from [1] | 0.0058 | optimized in the range 0.0010 - 0.0241 from [1] |
| Insulin receptor bound | IRins(0) | 6.7692 | optimized in the range 0 - 98.3174 from [1] | 11.1869 | optimized in the range 0 - 54.0728 from [1] |
| Phosphorylated and internalized insulin receptor | IRi_YP(0) | 0.0433 | optimized in the range 0.0200 - 0.3684 from [1] | 0.0340 | optimized in the range 0.0128 - 0.2007 from [1] |
| Free internalized insulin receptor | IRi(0) | 0.2557 | optimized in the range 0.1057 - 2.4239 from [1] | 0.3131 | optimized in the range 0.0581 - 1.3338from [1] |
| Insulin receptor substrate1 | IRS1(0) | 70.8957 | optimized in the range 32.8904-82.2711 from [1] | 85.3981 | optimized in the rang 74.9450 - 86.2120 from [1] |
| Insulin receptor substrate1 phosphorylated at tyrosine | IRS1_YP(0) | 0.0015 | optimized in the range 0.0012 - 0.0079 from [1] | 0.0027 | optimized in the range 0.0010 - 0.0128 from [1] |
| Insulin receptor substrate1 phosphorylated both at tyrosine and serine sites | IRS1_YP_S307P(0) | 1.6060 | optimized in the range 0.3945 - 12.9125 from [1] | 0.1011 | optimized in the range 0.0108 - 2.6372 from [1] |
| Insulin receptor substrate1 phosphorylated at serine site | IRS1_S307P(0) | 27.4967 | optimized in the range 17.3332 - 61.8749 from [1] | 14.4981 | optimized in the range 13.7761 - 23.9259 from [1] |
| Inactive feedback protein | X(0) | 90.8250 | optimized in the range 82.7446 - 92.1517 from [1] | 85.6979 | optimized in the range 74.2898 - 93.2612 from [1] |
| Active feedback protein | X_P(0) | 9.1750 | optimized in the range 7.8483 - 17.2554 from [1] | 14.3021 | optimized in the range 6.7388- 25.7102 from [1] |
| Protein kinase b | PKB(0) | 45.0895 | optimized in the range 2.3591 - 66.7261 from [1] | 41.7141 | optimized in the range 5.4955 - 75.9049 from [1] |
| PKB phosphorylated at threonine site | PKB_T308P(0) | 10.9438 | optimized in the range 1.4914 - 33.8840 from[1] | 17.8142 | optimized in the range 6.3922 - 50.9079 from [1] |
| PKB phosphorylated at serine site | PKB_S473P(0) | 29.6843 | optimized in the range 9.4834 - 37.0490 from [1] | 39.1462 | optimized in the range 11.1110 - 61.5802 from [1] |
| PKB phosphorylated both at threonine and at serine sites | PKB_T308P_S473P(0) | 14.2824 | optimized in the range 2.1594 - 71.6300 from [1] | 1.3253 | optimized in the range 0.0771 - 33.7498 from [1] |
| Protein complex mTORC1 | mTORC1(0) | 48.0015 | optimized in the range 15.8212 - 84.0562 from[1] | 87.8940 | optimized in the range 28.3447 - 96.6769 from [1] |
| Active protein complex mTORC1 | mTORC1a(0) | 51.9985 | optimized in the range 15.9438 - 84.1788 from [1] | 12.1060 | optimized in the range 3.3231 - 71.6553 from [1] |
| Protein complex mTORC2 | mTORC2(0) | 99.6572 | optimized in the range 97.7928 - 99.8383 from [1] | 99.7289 | optimized in the range 98.9110 - 99.8963 from [1] |
| Active protein complex mTORC2 | mTORC2a(0) | 0.3428 | optimized in the range 0.1617 - 2.2072 from [1] | 0.2711 | optimized in the range 0.1037 - 1.0890 from [1] |
| Substrate of PKB | AS160(0) | 56.5941 | optimized in the range 24.6694 - 83.3321 from [1] | 76.9252 | optimized in the range 37.2542 - 93.1897 from [1] |
| Substrate of PKB phosphorylated form | AS160_T642P(0) | 43.4059 | optimized in the range 16.6679 - 75.3306 from [1] | 23.0747 | optimized in the range 6.8103 - 62.7458 from [1] |
| Glucose transporter 4 in membrane | GLUT4m(0) | 49.1528 | optimized in the range 27.0715 - 62.6541 from [1] | 16.9725 | optimized in the range 6.5848 - 29.1440 from [1] |
| Glucose transporter 4 in cytosol | GLUT4(0) | 50.8472 | optimized in the range 37.3459 - 72.9285 from [1] | 33.0275 | optimized in the range 20.8560 - 43.4152 from[1] |
| S6 kinase | S6K(0) | 86.3744 | optimized in the range 83.8342 - 95.5400 from [1] | 95.5986 | optimized in the range 89.0410 - 99.0364 from [1] |
| S6 kinase phosphorylated form | S6K_T389P(0) | 13.6256 | optimized in the range 4.4600 - 16.1658 from [1] | 4.4014 | optimized in the range 0.9636 - 10.9590 from [1] |
| Ribosomal protein S6 | S6(0) | 40.4757 | optimized in the range 36.5506 - 67.6139 from [2] | 69.5055 | optimized in the range 45.9399 - 90.6217 from [2] |
| Phosphorylated ribosomal protein S6 | S6_S235_S236P(0) | 59.5243 | optimized in the range 32.3861 - 63.4494 from[2] | 30.4945 | optimized in the range 9.3783 - 54.0601 from[2] |

| Table B. **Physiological ranges of the whole body variables in the normal glucose regulation (NGR) condition (see also Figure 3). The ranges for H, S and L have not been specified, as there is no clear upper limit to the amount of food (or glucose) one could ingest.** | | | |
| --- | --- | --- | --- |
|  | | | |
| Variable description | Symbol | Range | Reference |
| Plasma glucose concentration | G | 4.5 - 11 mM | [5][13] |
| Plasma insulin concentration | I | 38 - 400 pM | [7] |
| Plasma incretin concentration | W | 5 - 50 pM | [14] |
| Plasma glucagon concentration | E | 28.68 - 47.04 pM | [7] |
| Liver glucose mass | C | 0 - 8 mmol | [8] |
| Muscle tissue glucose mass | M | 2 - 13 mmol | [8] |
| Adipose tissue glucose mass | A | 30 - 120 mmol | [8] |
| Plasma leptin concentration | Y | 0 - 0.6 nM | [15] |
| Plasma ghrelin concentration | Q | 8 - 146 pM | [10] |
| Amount of glucose intake | H | n.a. |  |
| Stomach glucose mass | S | n.a. |  |
| Intestine glucose mass | L | n.a. |  |
| Legend: n.a., not available | | | |

| Table C. **Parameter estimates of the model in the normal glucose regulation (NGR) and T2DM conditions. Values have been computed according to the estimation procedure described in the text.** | | | | | |
| --- | --- | --- | --- | --- | --- |
|  | | | | | |
| **Parameter description** | **Symbol** | **NGR** | **Estimation Procedure** | **T2DM** | **Estimation Procedure** |
| Insulin independent glucose utilization | $\text{b}_{\text{1}}$ | $\text{0.0059}\text{ }$  $\text{mi}\text{n}^{-\text{1}}$ | calculated as in$\text{ }$[16] [5] | $\text{0.0049}\text{ }\text{mi}\text{n}^{-\text{1}}$ | taken from[5] |
| Insulin disappearance rate | $\text{b}_{\text{2}}$ | $\text{0.1262}\text{ }$  $\text{mi}\text{n}^{-\text{1}}$ | taken from [17] | $\text{0.04}\text{ }\text{mi}\text{n}^{-\text{1}}$ | taken from[5] |
| Insulin dependent glucose utilization | $\text{b}_{\text{3}}$ | $\text{0.00005}$  $\text{ (}\text{pM}\text{∙}\text{mi}{\text{n}\text{)}}^{-\text{1}}$ | taken from [17] | $\text{0.00002}$  $\text{ (}\text{pM}\text{∙}\text{mi}{\text{n}\text{)}}^{-\text{1}}$ | taken from [5] |
| Glucose dependent insulin secretion | $\text{b}_{\text{4}}$ | $\text{0.4543}\text{ }$  $\text{pM}/\text{mM}\text{∙}\text{min}$ | calculated by steady state analysis of Eq. (4) at time 0 as in [5] | $\text{0.15}\text{ }$  $\text{pM}/\text{mM}\text{∙}\text{min}$ | calculated by steady state analysis of Eq. (4) at time 0 as in$\text{ }$[5] |
| Glucose transfer from liver to plasma | $\text{b}_{\text{5}}$ | $\text{0.185 }$  $\text{mi}\text{n}^{-\text{1}}$ | calculated by steady state analysis of Eq. (3) at time 0 as in [5] | $\text{0.3320}\text{ }\text{mi}\text{n}^{-\text{1}}$ | calculated by steady state analysis of Eq. (3) at time 0 as in [5] |
| Intestine glucose dependent incretin secretion | $\text{b}_{\text{6}}$ | $\text{0.0102}\text{ }$  $\text{pM}/\text{mmol}\text{∙}\text{min}$ | set equal to the estimate computed for T2DM according to [18] [19] | $\text{0.0102}\text{ }$  $\text{pM}/\text{mmol}\text{∙}\text{min}$ | optimized in $\text{the range}$  $\text{ }\text{0.0102}\text{-0.0104 }$  $\text{pM}/\text{mmol}\text{∙}\text{min}$ from [5] |
| Incretin disappearance rate | $\text{b}_{\text{7}}$ | $\text{0.03}$  $\text{ }\text{mi}\text{n}^{-\text{1}}$ | set equal to the estimate computed for T2DM according to [18] [19] | $\text{0.03}$  $\text{ }\text{mi}\text{n}^{-\text{1}}$ | optimized in the range $\text{0.03}$-0.06  $\text{ }\text{mi}\text{n}^{-\text{1}}$ from [5] |
| Stomach glucose emptying rate | $\text{b}_{\text{8}}$ | $\text{0.022}$  $\text{ }\text{mi}\text{n}^{-\text{1}}$ | optimized in the range 0.020±0.002$\text{ mi}\text{n}^{-\text{1}}$ from [20] | $\text{0.022}$  $\text{ }\text{mi}\text{n}^{-\text{1}}$ | optimized in the range 0.020±0.002$\text{ mi}\text{n}^{-\text{1}}$ from [20] |
| Glucose transfer rate to the stomach | $\text{b}_{\text{9}}$ | $\text{0.022}\text{ }$  $\text{mi}\text{n}^{-\text{1}}$ | unconstrained optimization | $\text{0.022}\text{ }$  $\text{mi}\text{n}^{-\text{1}}$ | unconstrained optimization |
| Intestine glucose emptying rate | $\text{b}_{\text{10}}$ | $\text{0.022}\text{ }$  $\text{mi}\text{n}^{-\text{1}}$ | optimized in the range 0.020±0.002$\text{ mi}\text{n}^{-\text{1}}$ from [20] | $\text{0.022}\text{ }$  $\text{mi}\text{n}^{-\text{1}}$ | optimizing in the range 0.020±0.002$\text{ mi}\text{n}^{-\text{1}}$ from [20] |
| Ghrelin disappearance | $\text{b}_{\text{11}}$ | $\text{0.02}\text{ }$  $\text{mi}\text{n}^{-\text{1}}$ | set equal to the estimate computed for T2DM | $\text{0.02}\text{ }$  $\text{mi}\text{n}^{-\text{1}}$ | taken from [5] |
| The appearance constant for ghrelin | $\text{b}_{\text{12}}$ | $\text{28.66}\text{ }$  $\text{pM}/\text{min}$ | calculated by steady state analysis of Eq. (11) at time 0 as in [5] | $\text{7 }\text{pM}/\text{min}$ | calculated by steady state analysis of Eq. (11) at time 0 as in$\text{ }$[5] |
| Leptin secretion rate | $\text{b}_{\text{13}}$ | $\text{0.0000095 }$  $\text{nM}\text{/}\text{mmol}\text{∙}\text{mi}\text{n}\text{∙}\text{kg}$ | calculated by steady state analysis of Eq. (13) at time 0 | $\text{0.000061 }$  $\text{nM}\text{/}\text{mmol}\text{∙}\text{mi}\text{n}\text{∙}\text{kg}$ | calculated by steady state analysis of Eq. (13) at time 0 |
| Leptin disappearance | $\text{b}_{\text{14}}$ | $\text{0.0278}\text{ }\text{mi}\text{n}^{-\text{1}}$ | taken from [21] | $\text{0.0278}\text{ }\text{mi}\text{n}^{-\text{1}}$ | as in NGR |
| Ghrelin dependent glucose intake appearance | $\text{b}_{\text{17}}$ | $\text{0.7}$  $\text{ }\text{mmol}/\text{pM}\text{∙}\text{min}$ | calculated by steady state analysis of Eq. (12) at time 0 | $\text{1.2 }$  $\text{mmol}/\text{pM}\text{∙}\text{min}$ | calculated by steady state analysis of Eq. (12) at time 0 |
| Leptin inhibition on glucose intake | $\text{b}_{\text{18}}$ | $\text{0.35}\text{ }\text{n}\text{M}^{-\text{1}}$ | taken from [22] | $\text{0.23 }\text{n}\text{M}^{-\text{1}}$ | calculated as in$\text{ }$[23] |
| Glucose effect rate on glucose intake | $\text{b}_{\text{19}}$ | $\text{0.004}\text{ (}\text{mM}\text{∙}\text{mi}{\text{n}\text{)}}^{-\text{1}}$ | taken from [24] | $\text{0.004}\text{ (}\text{mM}\text{∙}\text{mi}{\text{n}\text{)}}^{-\text{1}}$ | taken from [24] |
| Glucagon action on the liver | $\text{b}_{\text{21}}$ | $\text{0.00876}\text{ }$  $\text{mmol}/\text{min}\text{∙}\text{pM}\text{ }$ | taken from [25] | $\text{0.00876}\text{ }$  $\text{mmol}/\text{min}\text{∙}\text{pM}$ | as in NGR |
| Glucose action on the liver | $\text{b}_{\text{2}\text{2}}$ | $\text{0.0}\text{021}$  $\text{ }\text{mi}\text{n}^{-\text{1}}$ | taken from [12] | $\text{0.0007 }\text{mi}\text{n}^{-\text{1}}$ | taken from [12] |
| Liver glucose constant production | $\text{b}_{\text{23}}$ | $\text{0.08}$  $\text{ }\text{mmol}/\text{min}$ | taken from [12] | $\text{0.12 }\text{mmol}/\text{min}$ | taken from [12] |
| Insulin action on the liver | $\text{b}_{\text{25}}$ | $\text{0.00026}$  $\text{mmol}/\text{min}\text{∙}\text{pM}$ | taken from [12] | $\text{0.00018 }$  $\text{mmol}/\text{min}\text{∙}\text{pM}$ | taken from $\text{ }$[12] |
| Muscle glucose disappearance | $\text{b}_{\text{27}}$ | $\text{0.014 }\text{mi}\text{n}^{-\text{1}}$ | calculated by steady state analysis of Eq. (8) at time 0 | $\text{0.0099 }\text{mi}\text{n}^{-\text{1}}$ | calculated by steady state analysis of Eq. (8) at time 0 |
| Incretin dependent insulin secretion | $\text{c}$ | $\text{0.1060}\text{ }$  $\text{(}\text{mM}\text{∙}\text{mi}{\text{n}\text{)}}^{-\text{1}}$ | calculated by steady state analysis of Eq. (4) at time 0 as in [5] | $\text{0.035}\text{ (}\text{mM}\text{∙}\text{mi}{\text{n}\text{)}}^{-\text{1}}$ | calculated by steady state analysis of Eq. (4) at time 0 as in$\text{ }$[5] |
| Glucagon basal secretion | $\text{c}_{\text{0}}$ | $\text{1.8854}$  $\text{ }\text{pM}/\text{min}$ | taken from [25] | $\text{1.8854 }\text{pM}/\text{min}$ | taken from [25] |
| Glucose action on glucagon | $\text{c}_{\text{1}}$ | $\text{198 }$  $\text{pM}\text{∙}\text{ }\text{n}\text{M}/\text{min}$ | taken from $\text{ }$[25] | $\text{198pM}\text{∙}\text{ }\text{n}\text{M}/\text{min}$ | taken from [25] |
| Insulin action on glucagon | $\text{c}_{\text{2}}$ | 94 pM | set equal to the estimate computed for T2DM | 94 pM | optimized to have the plasma glucagon dynamics inside the range discussed in [7] |
| Glucagon disappearance | $\text{c}_{\text{3}}$ | $\text{0.05}\text{54 }\text{mi}\text{n}^{-\text{1}}$ | calculated by steady state analysis of Eq. (6) at time 0 as in [25] | $\text{0.05}\text{24 }\text{mi}\text{n}^{-\text{1}}$ | calculated by steady state analysis of Eq. (6) at time 0 as in [25] |
| Insulin effectiveness | $\text{e}$ | $\text{1}$ | taken from [25] | $\text{0.2}$ | taken from [25] |
| The fraction of absorbed glucose | $\text{f}$ | $\text{0.9}$ | taken from [5] | $\text{0.9}$ | taken from [5] |
| Averaged total fat mass in humans | $\text{Fat}$ | $\text{22}$ kg | calculated as in [21] assuming a standard body weight of 75 kg | $\text{27}$ kg | calculated as in [21] assuming a standard body weight of 90 kg |
| Glucose threshold value | $\text{G}_{\text{e}}$ | $\text{5}\text{ }\text{mM}$ | taken from $\text{ }$ [26] | $\text{8}\text{ }\text{mM}$ | taken from [26] |
| Glucose in stomach dependent decay rate of ghrelin | $\text{l}$ | $\text{0.006}\text{ }\text{mmo}\text{l}^{-\text{1}}$ | calculated as in [5] | $\text{0.006}\text{ }\text{mmo}\text{l}^{-\text{1}}$ | calculated as in [5] |
| Insulin dependent decay rate of ghrelin | $\text{m}$ | $\text{0.04}\text{ }\text{p}\text{M}^{-\text{1}}$ | calculated as in [5] | $\text{0.01}\text{ }\text{p}\text{M}^{-\text{1}}$ | calculated as in [5] |
| Insulin dependent decay rate of glucose intake | $\text{r}$ | $\text{0.04}\text{ }\text{p}\text{M}^{-\text{1}}$ | unconstrained optimization | $\text{0.01}\text{ }\text{p}\text{M}^{-\text{1}}$ | unconstrained optimization |
| Incretin constant secretion | $\text{s}$ | $\text{0.0}\text{3 }\frac{\text{pM}}{\text{min}}$ | set equal to the estimate computed for T2DM as in [18] [19] | $\text{0.0}\text{3 }\frac{\text{pM}}{\text{min}}$ | calculated as in [5] |
| Glucose distribution volume | $\text{v}$ | $\text{15}\text{ }\text{liters}$ | calculated as in [16] [5] assuming a standard body weight of 75 kg | $\text{18}\text{ }\text{liters}$ | calculated as in [16] [5] assuming a standard body weight of 90 kg |
| Insulin binding with IR | $\text{k}_{\text{1a}}$ | 0.00333$\text{ }\text{(}\text{mi}{\text{n}\text{∙}\text{pM}\text{)}}^{-\text{1}}$ | optimized in the range  (1e-6 - 1e6) from [1] for being consistent with [3] by also preserving the fits of Figures 5 and 6 | 0.00333$\text{ }\text{(}\text{mi}{\text{n}\text{∙}\text{pM}\text{)}}^{-\text{1}}$ | optimized in the range  (1e-6 - 1e6) from [1] for being consistent with [3] by also preserving the fits of Figures 5 and 6 |
| Basal phosphorylation of IR | $\text{k}_{\text{1basal}}$ | 0.0368$\text{ mi}\text{n}^{-\text{1}}$ | taken from [1] | 0.0368$\text{ mi}\text{n}^{-\text{1}}$ | taken from [1] |
| Phosphorylation of IRins | $\text{k}_{\text{1c}}$ | 0.877$\text{ mi}\text{n}^{-\text{1}}$ | taken from [1] | 0.877$\text{ mi}\text{n}^{-\text{1}}$ | taken from [1] |
| Endocytosis process of IR YP | $\text{k}_{\text{1d}}$ | 31.0$\text{ mi}\text{n}^{-\text{1}}$ | taken from [1] | 31.0$\text{ mi}\text{n}^{-\text{1}}$ | taken from [1] |
| Feedback from protein X_P | $\text{k}_{\text{1f}}$ | 0.368$\text{ mi}\text{n}^{-\text{1}}$ | taken from [1] | 0.368$\text{ mi}\text{n}^{-\text{1}}$ | taken from [1] |
| Basal dephosphorylation of IR YP | $\text{k}_{\text{1g}}$ | 1940$\text{ mi}\text{n}^{-\text{1}}$ | taken from [1] | 1940$\text{ mi}\text{n}^{-\text{1}}$ | taken from [1] |
| Recycling rate of the free insulin receptor | $\text{k}_{\text{1r}}$ | 0.547$\text{ mi}\text{n}^{-\text{1}}$ | taken from [1] | 0.547$\text{ mi}\text{n}^{-\text{1}}$ | taken from [1] |
| Phosphorylation of IRS1 from the receptor | $\text{k}_{\text{2a}}$ | 3.23$\text{ mi}\text{n}^{-\text{1}}$ | taken from [1] | 3.23$\text{ mi}\text{n}^{-\text{1}}$ | taken from [1] |
| Phosphorylation of IRS1 from the mTORC1a | $\text{k}_{\text{2c}}$ | 5760$\text{ mi}\text{n}^{-\text{1}}$ | taken from [1] | 5760$\text{ mi}\text{n}^{-\text{1}}$ | taken from [1] |
| Basal phosphorylation of IRS1 | $\text{k}_{\text{2basal}}$ | 0.0423$\text{ mi}\text{n}^{-\text{1}}$ | taken from [1] | 0.0423$\text{ mi}\text{n}^{-\text{1}}$ | taken from [1] |
| Dephosphorylation of IRS1 | $\text{k}_{\text{2b}}$ | 3420$\text{ mi}\text{n}^{-\text{1}}$ | taken from [1] | 3420$\text{ mi}\text{n}^{-\text{1}}$ | taken from [1] |
| Dephosphorylation of IRS1 in the serine site | $\text{k}_{\text{2d}}$ | 281$\text{ mi}\text{n}^{-\text{1}}$ | taken from [1] | 281$\text{ mi}\text{n}^{-\text{1}}$ | taken from [1] |
| Dephosphorylation of IRS1 in the tyrosine site | $\text{k}_{\text{2f}}$ | 2.91$\text{ mi}\text{n}^{-\text{1}}$ | taken from [1] | 2.91$\text{ mi}\text{n}^{-\text{1}}$ | taken from [1] |
| Dephosphorylation of IRS1 in the serine site | $\text{k}_{\text{2g}}$ | 0.267$\text{ mi}\text{n}^{-\text{1}}$ | taken from [1] | 0.267$\text{ mi}\text{n}^{-\text{1}}$ | taken from [1] |
| Activation of X protein | $\text{k}_{\text{3a}}$ | 6.90$\text{ mi}\text{n}^{-\text{1}}$ | taken from [1] | 6.90$\text{ mi}\text{n}^{-\text{1}}$ | taken from [1] |
| Deactivation of X protein | $\text{k}_{\text{3b}}$ | 0.0988$\text{ mi}\text{n}^{-\text{1}}$ | taken from [1] | 0.0988$\text{ mi}\text{n}^{-\text{1}}$ | taken from [1] |
| Phosphorylation of PKB | $\text{k}_{\text{4a}}$ | 5790$\text{ mi}\text{n}^{-\text{1}}$ | taken from [1] | 5790$\text{ mi}\text{n}^{-\text{1}}$ | taken from [1] |
| Dephosphorylation of PKB | $\text{k}_{\text{4b}}$ | 34.8$\text{ mi}\text{n}^{-\text{1}}$ | taken from [1] | 34.8$\text{ mi}\text{n}^{-\text{1}}$ | taken from [1] |
| Phosphorylation of PKB in the serine site | $\text{k}_{\text{4c}}$ | 4.46$\text{ mi}\text{n}^{-\text{1}}$ | taken from [1] | 4.46$\text{ mi}\text{n}^{-\text{1}}$ | taken from [1] |
| Phosphorylation of PKB | $\text{k}_{\text{4e}}$ | 42.8$\text{ mi}\text{n}^{-\text{1}}$ | taken from [1] | 42.8$\text{ mi}\text{n}^{-\text{1}}$ | taken from [1] |
| Dephosphorylation of PKB | $\text{k}_{\text{4f}}$ | 144$\text{ mi}\text{n}^{-\text{1}}$ | taken from [1] | 144$\text{ mi}\text{n}^{-\text{1}}$ | taken from [1] |
| Dephosphorylation of PKB | $\text{k}_{\text{4h}}$ | 0.536$\text{ mi}\text{n}^{-\text{1}}$ | taken from [1] | 0.536$\text{ mi}\text{n}^{-\text{1}}$ | taken from [1] |
| Activation of mTORC1 complex | $\text{k}_{\text{5a1}}$ | 1.84$\text{ mi}\text{n}^{-\text{1}}$ | taken from [1] | 1.84$\text{ mi}\text{n}^{-\text{1}}$ | taken from [1] |
| Activation of mTORC1 complex | $\text{k}_{\text{5a2}}$ | 0.0551$\text{ mi}\text{n}^{-\text{1}}$ | taken from [1] | 0.0551$\text{ mi}\text{n}^{-\text{1}}$ | taken from [1] |
| Deactivation of mTORC1 complex | $\text{k}_{\text{5b}}$ | 24.8$\text{ mi}\text{n}^{-\text{1}}$ | taken from [1] | 24.8$\text{ mi}\text{n}^{-\text{1}}$ | taken from [1] |
| Activation of mTORC2 complex | $\text{k}_{\text{5c}}$ | 0.0858$\text{ mi}\text{n}^{-\text{1}}$ | taken from [1] | 0.0858$\text{ mi}\text{n}^{-\text{1}}$ | taken from [1] |
| Deactivation of mTORC2 complex | $\text{k}_{\text{5d}}$ | 1.06$\text{ mi}\text{n}^{-\text{1}}$ | taken from [1] | 1.06$\text{ mi}\text{n}^{-\text{1}}$ | taken from [1] |
| Activation of AS160 | $\text{k}_{\text{6a1}}$ | 2.65$\text{ mi}\text{n}^{-\text{1}}$ | taken from [1] | 2.65$\text{ mi}\text{n}^{-\text{1}}$ | taken from [1] |
| Activation of AS160 | $\text{k}_{\text{6a2}}$ | 0.410$\text{ mi}\text{n}^{-\text{1}}$ | taken from [1] | 0.410$\text{ mi}\text{n}^{-\text{1}}$ | taken from [1] |
| Deactivation of AS160 | $\text{k}_{\text{6b}}$ | 65.2$\text{ mi}\text{n}^{-\text{1}}$ | taken from [1] | 65.2$\text{ mi}\text{n}^{-\text{1}}$ | taken from [1] |
| Translocation of GLUT4 to the membrane | $\text{k}_{\text{7a}}$ | 51.0$\text{ mi}\text{n}^{-\text{1}}$ | taken from [1] | 51.0$\text{ mi}\text{n}^{-\text{1}}$ | taken from [1] |
| Translocation of GLUT4 to the cytosol | $\text{k}_{\text{7b}}$ | 2290$\text{ mi}\text{n}^{-\text{1}}$ | taken from [1] | 2290$\text{ mi}\text{n}^{-\text{1}}$ | taken from [1] |
| GLUT4 glucose uptake | $\text{k}_{\text{8}}$ | 0.5275$\text{ }\text{mmol}\text{/}\text{mi}\text{n}$ | taken from [27] | 0.5644 mmol/min | taken from [27] |
| GLUT1 glucose uptake | GLUT1 | 0.0283 mmol/min | taken from [27] | 0.032 mmol/min | taken from [27] |
| Dependence on interstitial glucose saturated | kmG4 | 146.851 mg/kg | taken from [27] | 146.851 mg/kg | as in NGR |
| Dependence on interstitial glucose saturated | kmG1 | 1.082 mg/kg | taken from [27] | 1.082 mg/kg | as in NGR |
| Intra-adipocitary glucose elimination rate | $\text{k}_{\text{gluc}}$ | 0.25$\text{ mi}\text{n}^{-\text{1}}$ | unconstrained optimization | 0.25$\text{ mi}\text{n}^{-\text{1}}$ | unconstrained optimization |
| Phosphorylation of S6K | $\text{k}_{\text{9a}}$ | 0.0013$\text{ mi}\text{n}^{-\text{1}}$ | taken from [1] | 0.0013$\text{ mi}\text{n}^{-\text{1}}$ | taken from [1] |
| Dephosphorylation of S6K | $\text{k}_{\text{9b}}$ | 0.0444$\text{ mi}\text{n}^{-\text{1}}$ | taken from [1] | 0.0444$\text{ mi}\text{n}^{-\text{1}}$ | taken from [1] |
| Dephosphorylation of S6 | $\text{k}_{\text{9b2}}$ | 30.9966$\text{ mi}\text{n}^{-\text{1}}$ | taken from[2] | 30.9966$\text{ mi}\text{n}^{-\text{1}}$ | taken from [2] |
| Phosphorylation of S6 | $\text{k}_{\text{9f2}}$ | 3.3289$\text{ mi}\text{n}^{-\text{1}}$ | taken from [2] | 3.3289$\text{ mi}\text{n}^{-\text{1}}$ | taken from [2] |
| Interstitial insulin rate of change | $\text{p}_{\text{2U}}$ | 0.033$\text{ mi}\text{n}^{-\text{1}}$ | taken from [27] | 0.033$\text{ mi}\text{n}^{-\text{1}}$ | as in NGR |
| Interstitial glucose elimination rate | $\text{q}_{\text{1}}$ | 0.0031 $\text{mi}\text{n}^{-\text{1}}$ | unconstrained optimization | 0.0025 $\text{mi}\text{n}^{-\text{1}}$ | unconstrained optimization |
| Glucose transfer rate from plasma to interstitium | $\text{q}_{\text{2}}$ | 0.4054 (mg/kg)/mM$\text{∙}$min | unconstrained optimization | 0.4365 (mg/kg)/mM$\text{∙}$min | unconstrained optimization |
| Positive feedback from mTORC1 reduced in T2DM condition | k_fb_ | 1 | taken from [1] | 0.15 | taken from [1] |

**3. Supplementary figures**


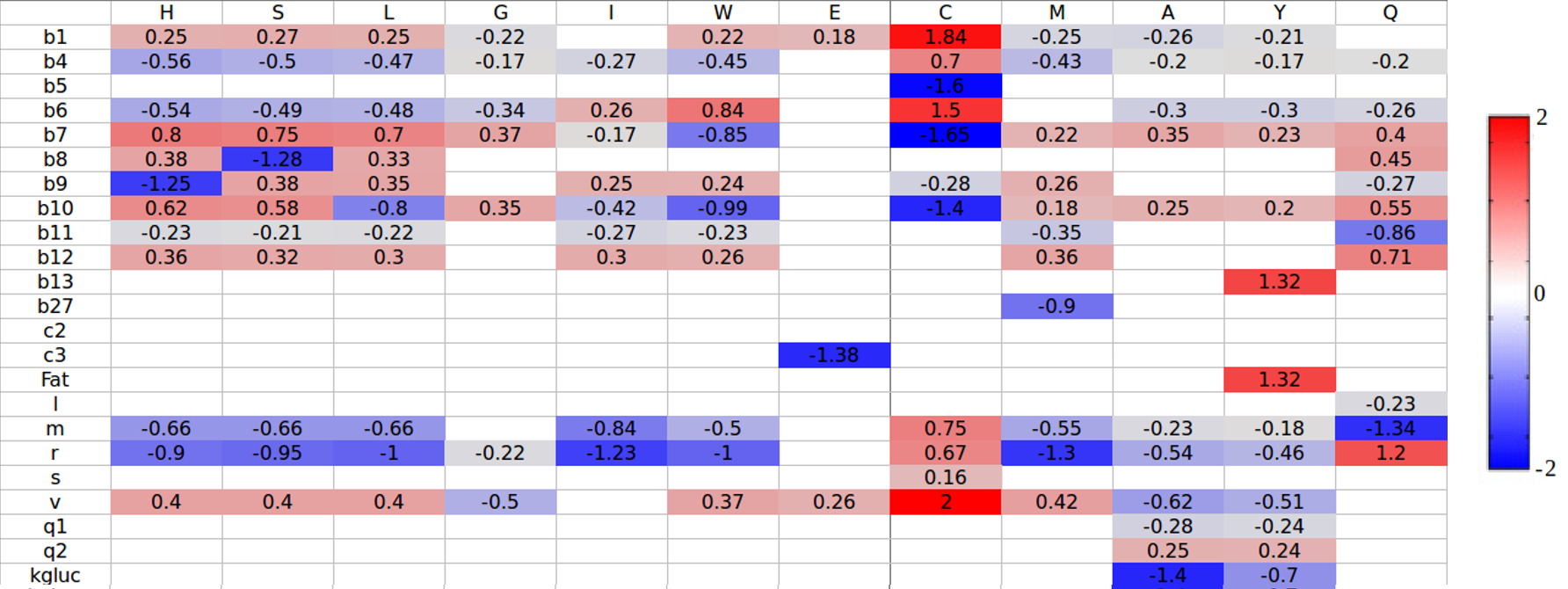


**Figure A.** Heatmap resuming the results of the sensitivity analysis by considering the parameter estimates computed for the normal glucose regulation (NGR) condition. For each parameter, the differences between the two AUC ratios computed with the maximum and the minimum considered value of $k$ are provided (${AUC}_{ratio}^{k=2}-{AUC}_{ratio}^{k=0.5}$), with respect to each model variable of the whole body model (see previous Section 3). Values lower than 0.15 have been considered negligible and are not displayed in the heatmap. The parameter $c_{2}$ does not affect model dynamics in the NGR condition, however, it has been estimated in the T2DM condition, where an impact on the system dynamics can be appreciated (see Figure B in S1 File).


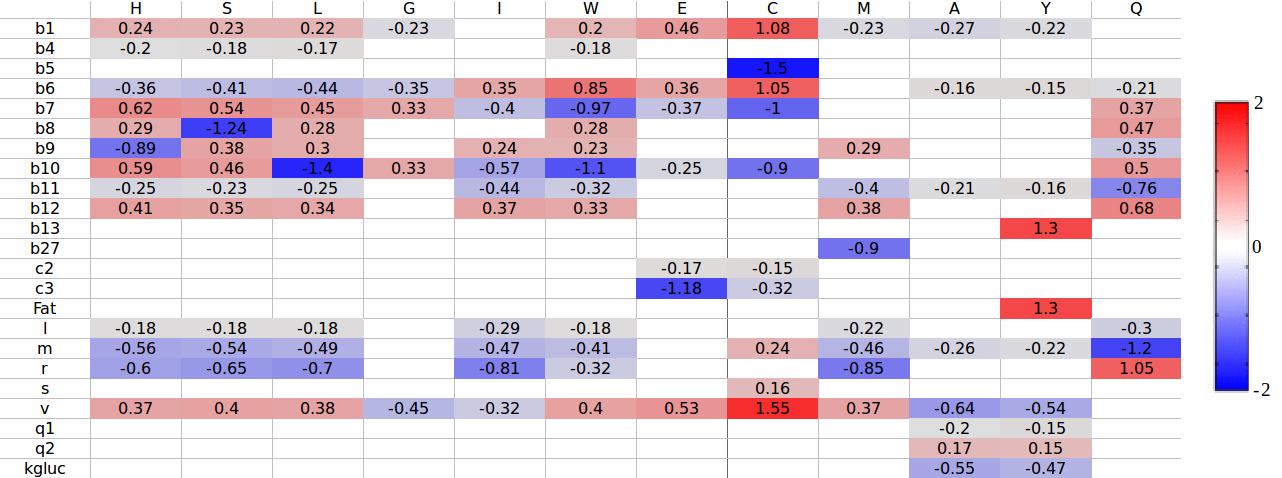


**Figure B.** Heatmap resuming the results of the sensitivity analysis by considering the parameter estimates computed for the T2DM condition. For each parameter, the differences between the two AUC ratios computed with the maximum and the minimum considered value of $k$ are provided (${AUC}_{ratio}^{k=2}-{AUC}_{ratio}^{k=0.5}$), with respect to each model variable of the whole body model (see previous Section 3). Values lower than 0.15 have been considered negligible and are not displayed in the heatmap.

**4. Background physiology**

- 1. **Glucose physiology at the whole-body level**

Many organs and hormones are involved in maintaining the physiological circulating plasma glucose concentration at equilibrium, with little deviations from 5.0 mM [4]. A graphical representation of the herein presented whole body model of glucose metabolism is shown in the main text, Figure 1. The breakdown of the meal constituents after oral feeding occurs after the meal transit through the stomach to the intestine, and it is ultimately resulting in an inward flux of micro- and macro-nutrients, including glucose, to the bloodstream. This event cascade induces endogenous insulin secretion, which is also amplified by a concomitant increase in the circulating levels of the so-called “incretin” hormones, including the gastric inhibitory polypeptide (GIP) and the glucagon-like peptide-1 (GLP1) [28]. Among the other neuroendocrine and gastro-intestinal tract effects, incretins potentiate the release and the *de novo* synthesis of insulin from pancreatic beta cells, thus paving the way for a proper glucose disposal. The incretin effect is responsible for about 50% to 70% of insulin secretion [5,29]. Insulin binds to its receptors on target organs, including, among others, the liver, muscle and adipose tissues stimulating glucose uptake. The skeletal muscle is responsible for a substantial proportion of glucose uptake (15-20%), while relatively smaller glucose proportions eventually reach the brain, kidney, blood cells, adipose tissue and the splanchnic organs [4].

The liver has a major role in the homeostasis of glucose, which is stored as glycogen in the postprandial state and released at fasting through endogenous hepatic gluconeogenesis. These mechanisms maintain plasma glucose levels within the physiological range in case of prolonged fasting, as it occurs overnight, for instance. Insulin acts on the liver by inhibiting gluconeogenesis and glycogen breakdown [13]. Another important player in liver glucose metabolism is glucagon, a hormone secreted from pancreatic alpha cells. Glucagon has the opposite effect of insulin, stimulating glycogen breakdown as plasma glucose levels decrease [4,30]. Glucagon secretion is inhibited by hyperglycemia and stimulated by hypoglycemia [4].

Glucose metabolism is also regulated by hunger and satiety, through a number of finely regulated biological mechanisms. Among the several hormones potentially involved in the regulation of glucose homeostasis, we focused on the reciprocal interactions occurring among insulin, ghrelin and leptin [31], as their mathematical specifications were available from existing literature (see the model description provided in the main text). Leptin is generally considered as the prototypical regulator of energy homeostasis, in that it signals from peripheral tissues (mainly the white adipose tissue) to the brain that energy stores are sufficient. As such, the circulating leptin levels appear to be directly related to the adipose tissue mass [32]. Fall in serum leptin levels -as it happens during prolonged starvation- in fact leads to downstream neuroendocrine alterations that prevent reproduction, reduce thyroid function, activate the hypothalamic-pituitary-adrenal axis, and inhibit the growth hormone. Ghrelin antagonizes the anorexigenic effect of leptin, and inversely parallels those of insulin, which is stimulated by meals, inhibits food intake, circulates in the fasting state in proportion to the body weight, and whose secretion is decreased following weight loss [5]. Here we simplified hunger as the amount of food needed by the organism [33]. We further abstracted this concept by considering hunger as the amount of ingested glucose, which depends on the circulating levels of leptin, ghrelin, insulin and ultimately also on extant glycaemia, so that we could make our model closed.

- 1. **Insulin signaling at the cellular (adipocyte) level**

1. A schematic representation of the model describing the insulin signaling cascade in adipocytes at the cellular level is shown in the main text, Figure 2. The insulin molecules contained in the interstitial space surrounding the adipocyte, bind to the insulin receptor (IR) on the cell surface causing its auto-phosphorylation, the activation of the tyrosine kinase domain, and the endocytosis of the IR. Substrate molecules, such as insulin receptor substrate 1 (IRS1), are then recruited and phosphorylated to start the insulin signaling cascade.
2. Insulin signaling is involved in different intersected pathways, such as glycogen synthesis, protein synthesis and cell survival. We focused on protein kinase B (PKB), whose activation is a key downstream event of insulin signaling: activated PKB stimulates the translocation of glucose receptor 4 (GLUT4) vesicles from its intracellular pool to the plasma membrane, allowing glucose uptake into the cell. The amount of glucose uptake depends on the number of GLUT4 on the cell surface, on the glucose and insulin levels in the interstitial space and on the amount of glucose receptor 1 (GLUT1). GLUT1 is responsible for basal glucose uptake [34], its expression is insulin independent and we modeled it as a constant value, following the same modeling approach introduced by Nyman *et al.* [1].

The phosphorylation of PKB also causes the activation of the protein complex mTORC1, which has a feedback effect on insulin signaling through IRS1 phosphorylation. The reduction of this feedback effect is suggested as one of the main mechanisms of insulin resistance development. Similarly, the reduced amounts of IRs and GLUT4 are considered as indicators of diabetes [1].

**5. References**

1. Nyman E, Rajan MR, Fagerholm S, Brannmark C, Cedersund G, et al. (2014) A single mechanism can explain network-wide insulin resistance in adipocytes from obese patients with type 2 diabetes. J Biol Chem 289: 33215–33230. doi:10.1074/jbc.M114.608927.

2. Brännmark C, Nyman E, Fagerholm S, Bergenholm L, Ekstrand E-M, et al. (2013) Insulin signaling in Type 2 Diabetes: experimental and modeling analyses reveal mechanisms of insulin resistance in human adipocytes. J Biol Chem 288: 9867–9880. doi:10.1074/jbc.M112.432062.

3. Lodish H, Berk A, Kaiser CA, Krieger M (2007) Molecular cell biology 6th edition.

4. Poretsky L (2010) Normal glucose homeostasis. Principles of diabetes Mellitus 2nd edition. New York, USA: Springer. pp. 1–887. doi:10.1007/978-0-387-09841-8.

5. Toghaw P, Matone A, Lenbury Y, De Gaetano A (2012) Bariatric surgery and T2DM improvement mechanisms: a mathematical model. Theor Biol Med Model 9: 16. doi:10.1186/1742-4682-9-16.

6. Idorn T, Knop FK, Jorgensen M, Holst JJ, Hornum M, et al. (2014) Postprandial responses of incretin and pancreatic hormones in non-diabetic patients with end-stage renal disease. Nephrol Dial Transplant 29: 119–127. doi:10.1093/ndt/gft353.

7. Henkel E, Menschikowski M, Koehler C, Leonhardt W, Hanefeld M (2005) Impact of glucagon response on postprandial hyperglycemia in men with impaired glucose tolerance and type 2 diabetes mellitus. Metabolism 54: 1168–1173. doi:10.1016/j.metabol.2005.03.024.

8. Pratt AC, A.D. Wattis J, Salter AM (2015) Mathematical modelling of hepatic lipid metabolism. Math Biosci 262: 167–181. doi:10.1016/j.mbs.2014.12.012.

9. Klein S, Coppack SW, Mohammed-Ali V, Landt M (1996) Adipose tissue leptin production and plasma leptin kinectics in humans. Diabetes 45: 984–987. doi:10.2337/diabetes.45.7.984.

10. Engström BE, Ohrvall M, Sundbom M, Lind L, Karlsson FA (2007) Meal suppression of circulating ghrelin is normalized in obese individuals following gastric bypass surgery. Int J Obes 31: 476–480. doi:10.1038/sj.ijo.0803440.

11. EFSA Panel on Dietetic Products, Nutrition, (NDA) A (2010) Scientific Opinion on Dietary Reference Values for carbohydrates and dietary fibre. EFSA J 8: 1462–1477. doi:10.2903/j.efsa.2010.1462.Available.

12. Dalla Man C, Rizza RA, Cobelli C (2007) Meal simulation model of the glucose-insulin system. IEEE Trans Biomed Eng 54: 1740–1749. doi:10.1109/TBME.2007.893506.

13. Gerich JE (2000) Physiology of glucose homeostasis. Diabetes, Obes Metab 2: 345–350. doi:10.1046/j.1463-1326.2000.00085.x.

14. Seino Y, Fukushima M, Yabe D (2010) GIP and GLP-1, the two incretin hormones: similarities and differences. J Diabetes Investig 1: 8–23. doi:10.1111/j.2040-1124.2010.00022.x.

15. Askari H, Tykodi G, Liu J, Dagogo-Jack S (2010) Fasting plasma leptin level is a surrogate measure of insulin sensitivity. J Clin Endocrinol Metab 95: 3836–3843. doi:10.1210/jc.2010-0296.

16. Michaeli B, M. Berger M, Revelly JP, Tappy L, Chioléro R (2007) Effects of fish oil on the neuro-endocrine responses to an endotoxin challenge in healthy volunteers. Clin Nutr 26: 70–77. doi:10.1016/j.clnu.2006.06.001.

17. De Gaetano A, Arino O (2000) Mathematical modelling of the intravenous glucose tolerance test. J Math Biol 40: 136–168. doi:10.1007/s002850050007.

18. Vilsbøll T, Krarup T, Deacon CF, Madsbad S, Holst JJ (2001) Reduced postprandial concentrations of intact biologically active glucagon-like peptide 1 in type 2 diabetic patients. Diabetes 50: 609–613.

19. Meier JJ, Nauck MA (2010) Is the diminished incretin effect in type 2 diabetes just an epi-phenomenon of impaired β-cell function? Diabetes 59: 1117–1125. doi:10.2337/db09-1899.

20. Schirra J, Katschinski M, Weidmann C, Schäfer T, Wank U, et al. (1996) Gastric emptying and release of incretin hormones after glucose ingestion in humans. J Clin Invest 97: 92–103. doi:10.1172/JCI118411.

21. Grasman J (2013) Reconstruction of the drive underlying food intake and its control by leptin and dieting. PLoS One 8: e74997. doi:10.1371/journal.pone.0074997.

22. Golden PL, Maccagnan TJ, Pardridge WM (1997) Human blood-brain barrier leptin receptor. Binding and endocytosis in isolated human brain microvessels. J Clin Invest 99: 14–18. doi:10.1172/JCI119125.

23. Banks WA (2012) Role of the blood-brain barrier in the evolution of feeding and cognition. Ann N Y Acad Sci 1264: 13–19. doi:10.1111/j.1749-6632.2012.06568.x.

24. Gruetter R, Ugurbil K, Seaquist ER (1998) Steady-state cerebral glucose concentrations and transport in the human brain. J Neurochem 70: 397–408.

25. Sulston KW, Ireland WP, Praught JC (2006) Hormonal effects on glucose regulation. Atlantic 1: 31–46.

26. König M, Holzhütter H-G (2012) Kinetic modeling of human hepatic glucose metabolism in type 2 diabetes mellitus predicts higher risk of hypoglycemic events in rigorous insulin therapy. J Biol Chem 287: 36978–36989. doi:10.1074/jbc.M112.382069.

27. Nyman E, Brannmark C, Palmer R, Brugard J, Nystrom FH, et al. (2011) A hierarchical whole-body modeling approach elucidates the link between in vitro insulin signaling and in vivo glucose homeostasis. J Biol Chem 286: 26028–26041. doi:10.1074/jbc.M110.188987.

28. De Gaetano A, Panunzi S, Matone A, Samson A, Vrbikova J, et al. (2013) Routine OGTT: a robust model including incretin effect for precise identification of insulin sensitivity and secretion in a single individual. PLoS One 8: e70875. doi:10.1371/journal.pone.0070875.

29. Nauck MA, Homberger E, Siegel EG, Allen RC, Eaton RP, et al. (1986) Incretin effects of increasing glucose loads in man calculated from venous insulin and c-peptide responses. Clin Endoc Meta 63: 492–498.

30. Gromada J, Franklin I, Wollheim CB (2007) α-Cells of the endocrine pancreas: 35 years of research but the enigma remains. Endocr Rev 28: 84–116. doi:10.1210/er.2006-0007.

31. Cummings DE (2001) A preprandial rise in plasma ghrelin levels suggests a role in meal initiation in humans. Diabetes 50: 1714–1719.

32. Jéquier E, Tappy L (1999) Regulation of body weight in humans. Physiol Rev 79: 451–480.

33. Jacquier M, Crauste F, Soulage CO, Soula HA (2014) A predictive model of the dynamics of body weight and food intake in rats submitted to caloric restrictions. PLoS One 9: e100073. doi:10.1371/journal.pone.0100073.

34. Hall JR, Clow KA, Short CE, Driedzic WR (2014) Transcript levels of class I GLUTs within individual tissues and the direct relationship between GLUT1 expression and glucose metabolism in Atlantic cod (Gadus morhua). J Comp Physiol B 184: 483–496. doi:10.1007/s00360-014-0810-7.

1. The Microsoft Research – University of Trento Centre for Computational and Systems Biology (COSBI), Piazza Manifattura 1, 38068 Rovereto (TN), Italy

   2 Division of Endocrinology, Diabetes and Metabolism, Department of Medicine, University of Verona, Verona, Italy

   3 Department of Computer Science, University of Pisa, Italy

   * C.U. and G.S. share first authorship. Communications to be addressed to C.P. (priami@cosbi.eu). [↑](#footnote-ref-1)
