# Supplementary material for: A closed-loop multi-level model of glucose homeostasis
Source: PLoS One. 2018 Feb 8;13(2):e0190627. doi: 10.1371/journal.pone.0190627 (PMC5805234; doi:10.1371/journal.pone.0190627)
Supplement: S2 File — (PDF) [file pone.0190627.s002.pdf]

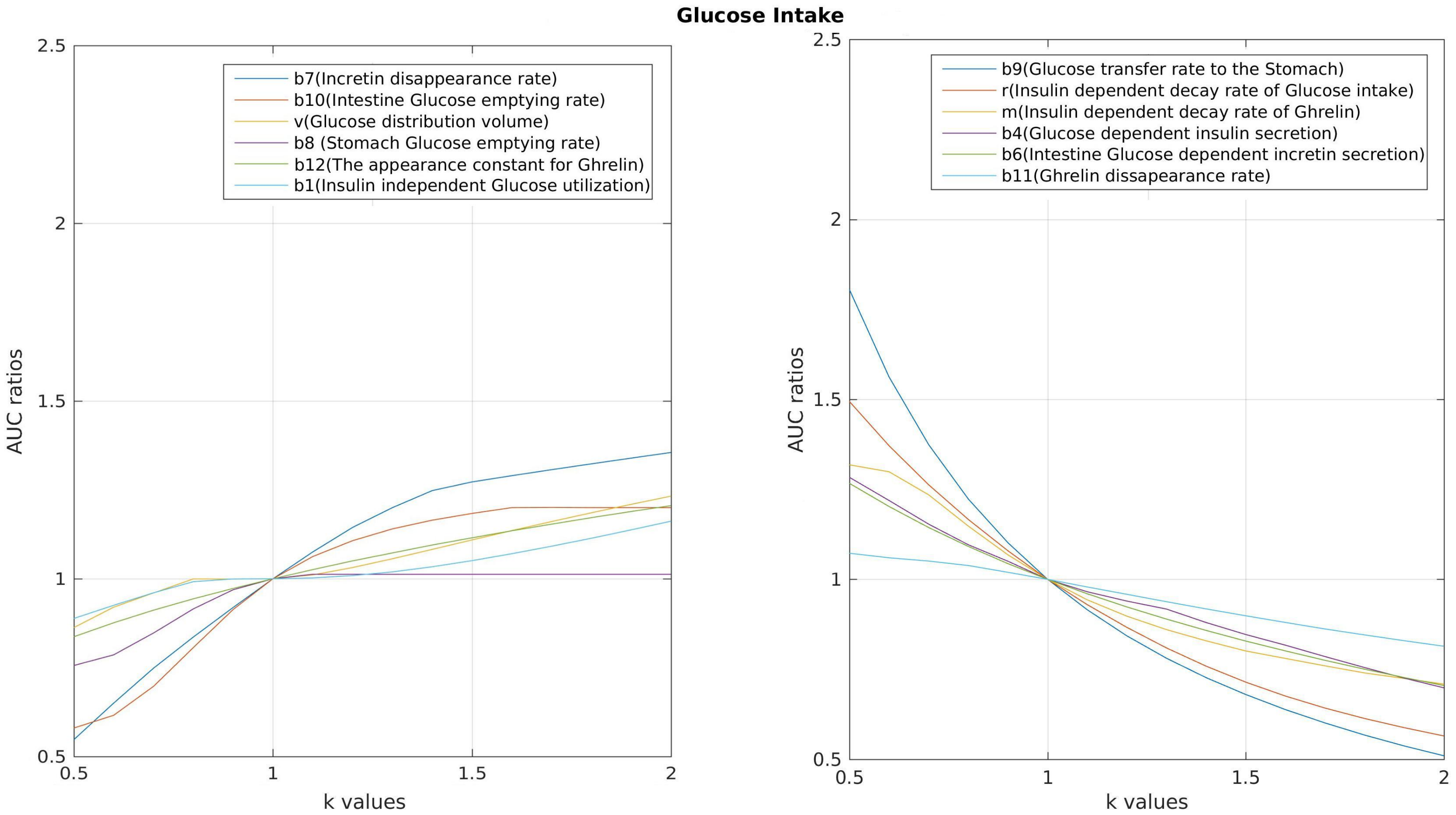
Glucose Intake
b7(Incretin disappearance rate)
b10(Intestine Glucose emptying rate)
v(Glucose distribution volume)
b8 (Stomach Glucose emptying rate)
b12(The appearance constant for Ghrelin)
b1(Insulin independent Glucose utilization)
AUC ratios
k values
b9(Glucose transfer rate to the Stomach)
r(Insulin dependent decay rate of Glucose intake)
m(Insulin dependent decay rate of Ghrelin)
b4(Glucose dependent insulin secretion)
b6(Intestine Glucose dependent incretin secretion)
b11(Ghrelin dissapearance rate)
AUC ratios
k values

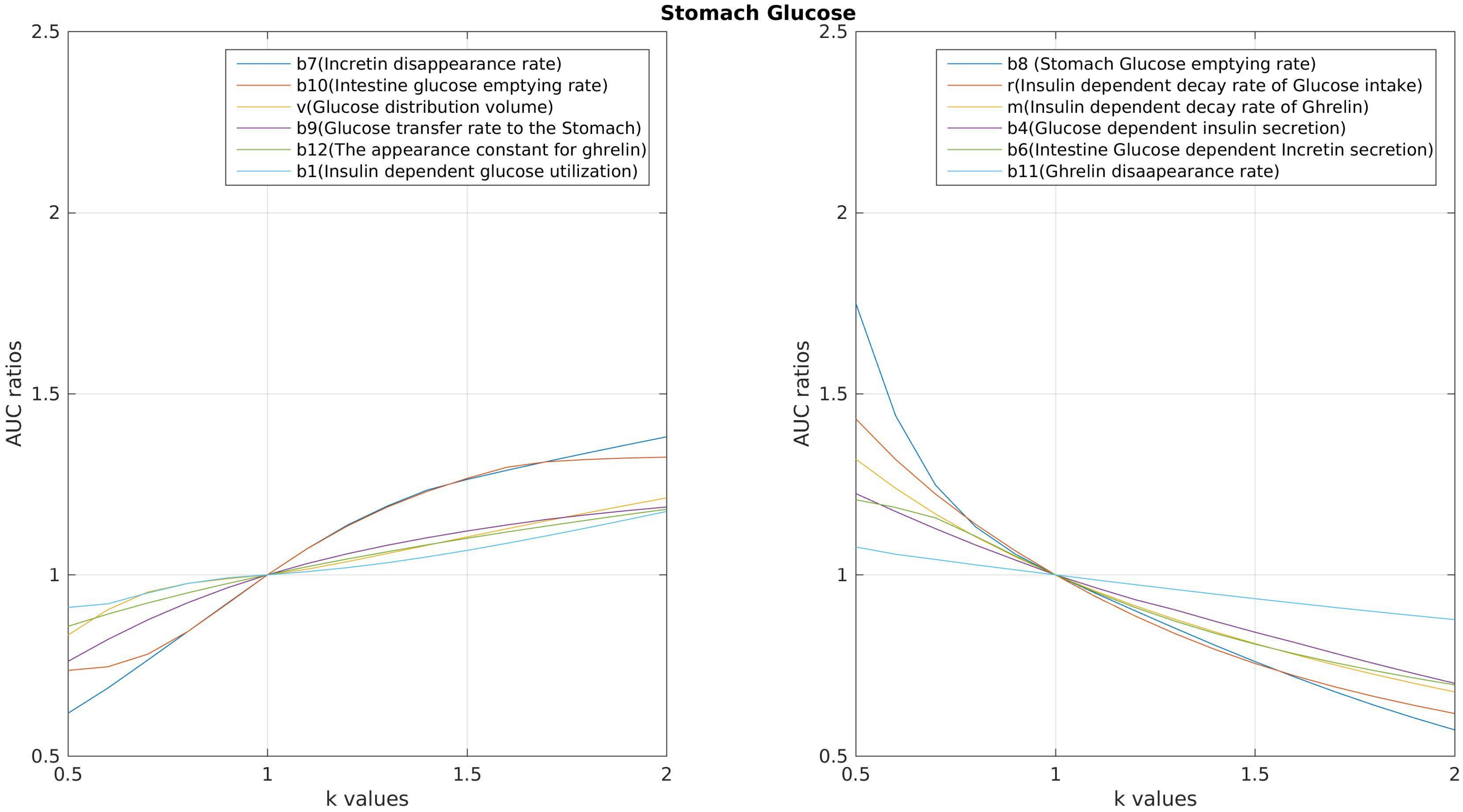
Stomach Glucose
b7(Incretin disappearance rate)
b10(Intestine glucose emptying rate)
v(Glucose distribution volume)
b9(Glucose transfer rate to the Stomach)
b12(The appearance constant for ghrelin)
b1(Insulin dependent glucose utilization)
b8 (Stomach Glucose emptying rate)
r(Insulin dependent decay rate of Glucose intake)
m(Insulin dependent decay rate of Ghrelin)
b4(Glucose dependent insulin secretion)
b6(Intestine Glucose dependent Incretin secretion)
b11(Ghrelin disaapearance rate)
AUC ratios
k values
0.5
1
1.5
2
2.5

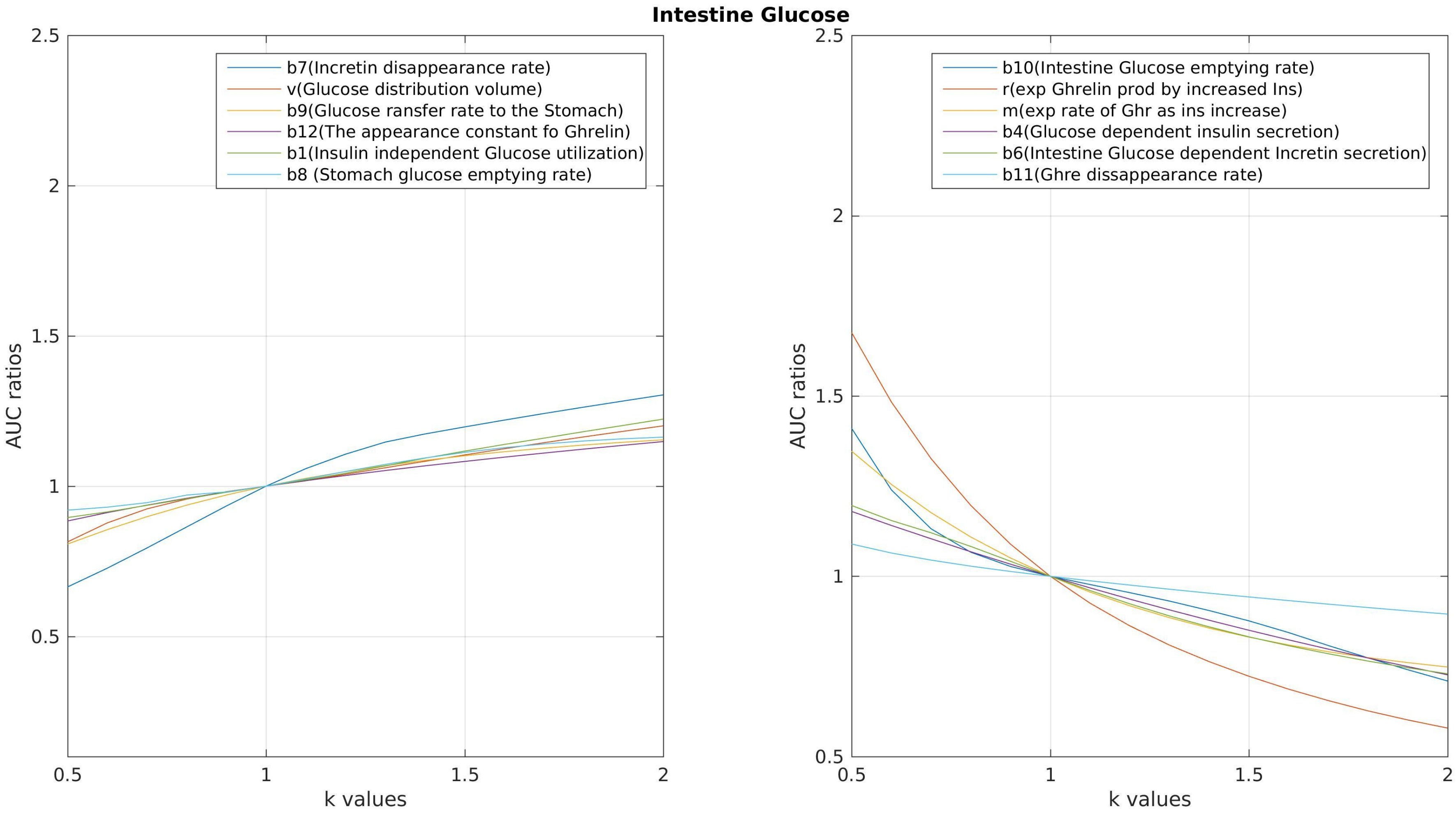
Intestine Glucose
AUC ratios
k values
b7(Incretin disappearance rate)
v(Glucose distribution volume)
b9(Glucose ransfer rate to the Stomach)
b12(The appearance constant fo Ghrelin)
b1(Insulin independent Glucose utilization)
b8 (Stomach glucose emptying rate)
b10(Intestine Glucose emptying rate)
r(exp Ghrelin prod by increased Ins)
m(exp rate of Ghr as ins increase)
b4(Glucose dependent insulin secretion)
b6(Intestine Glucose dependent Incretin secretion)
b11(Ghre dissappearance rate)

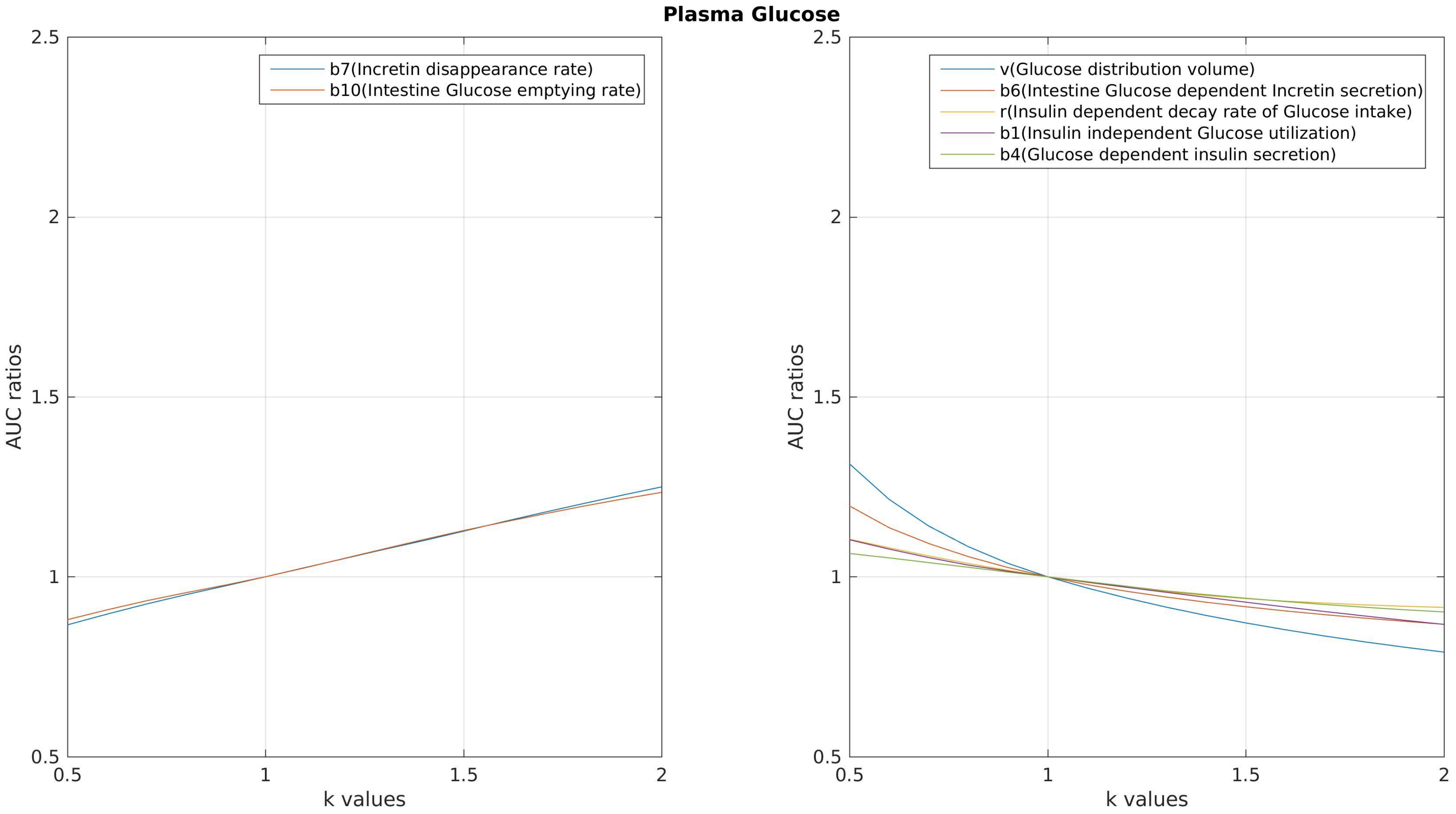
Plasma Glucose
AUC ratios
k values
b7(Incretin disappearance rate)
b10(Intestine Glucose emptying rate)
v(Glucose distribution volume)
b6(Intestine Glucose dependent Incretin secretion)
r(Insulin dependent decay rate of Glucose intake)
b1(Insulin independent Glucose utilization)
b4(Glucose dependent insulin secretion)
0.5
1
1.5
2
2.5

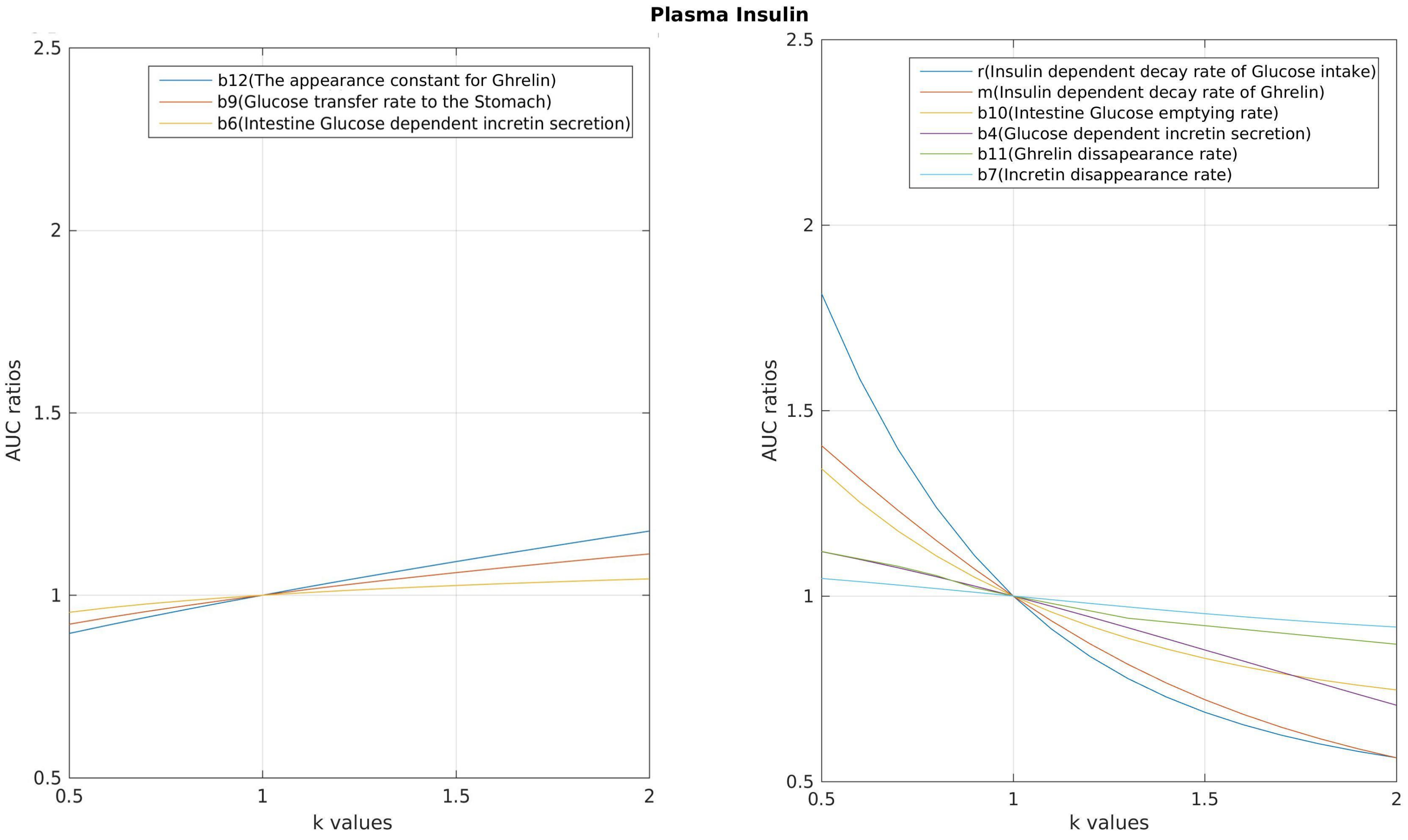

Plasma Insulin
AUC ratios
k values
b12(The appearance constant for Ghrelin)
b9(Glucose transfer rate to the Stomach)
b6(Intestine Glucose dependent incretin secretion)
r(Insulin dependent decay rate of Glucose intake)
m(Insulin dependent decay rate of Ghrelin)
b10(Intestine Glucose emptying rate)
b4(Glucose dependent incretin secretion)
b11(Ghrelin dissapearance rate)
b7(Incretin disappearance rate)
0.5
1
1.5
2
2.5

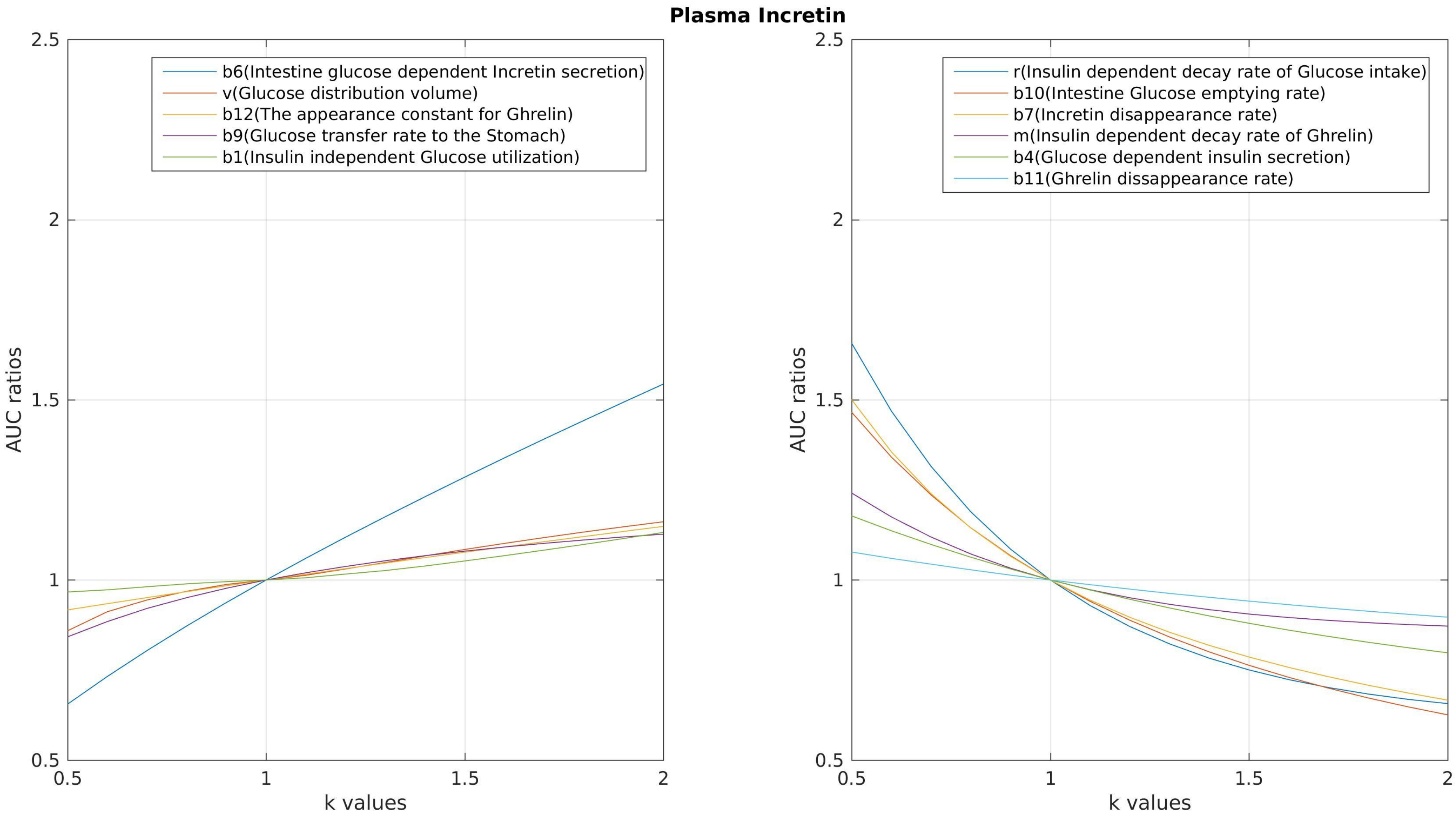
Plasma Incretin
b6(Intestine glucose dependent Incretin secretion)
v(Glucose distribution volume)
b12(The appearance constant for Ghrelin)
b9(Glucose transfer rate to the Stomach)
b1(Insulin independent Glucose utilization)
r(Insulin dependent decay rate of Glucose intake)
b10(Intestine Glucose emptying rate)
b7(Incretin disappearance rate)
m(Insulin dependent decay rate of Ghrelin)
b4(Glucose dependent insulin secretion)
b11(Ghrelin dissappearance rate)
AUC ratios
k values
2.5
2
1.5
1
0.5
0.5
1
1.5
2

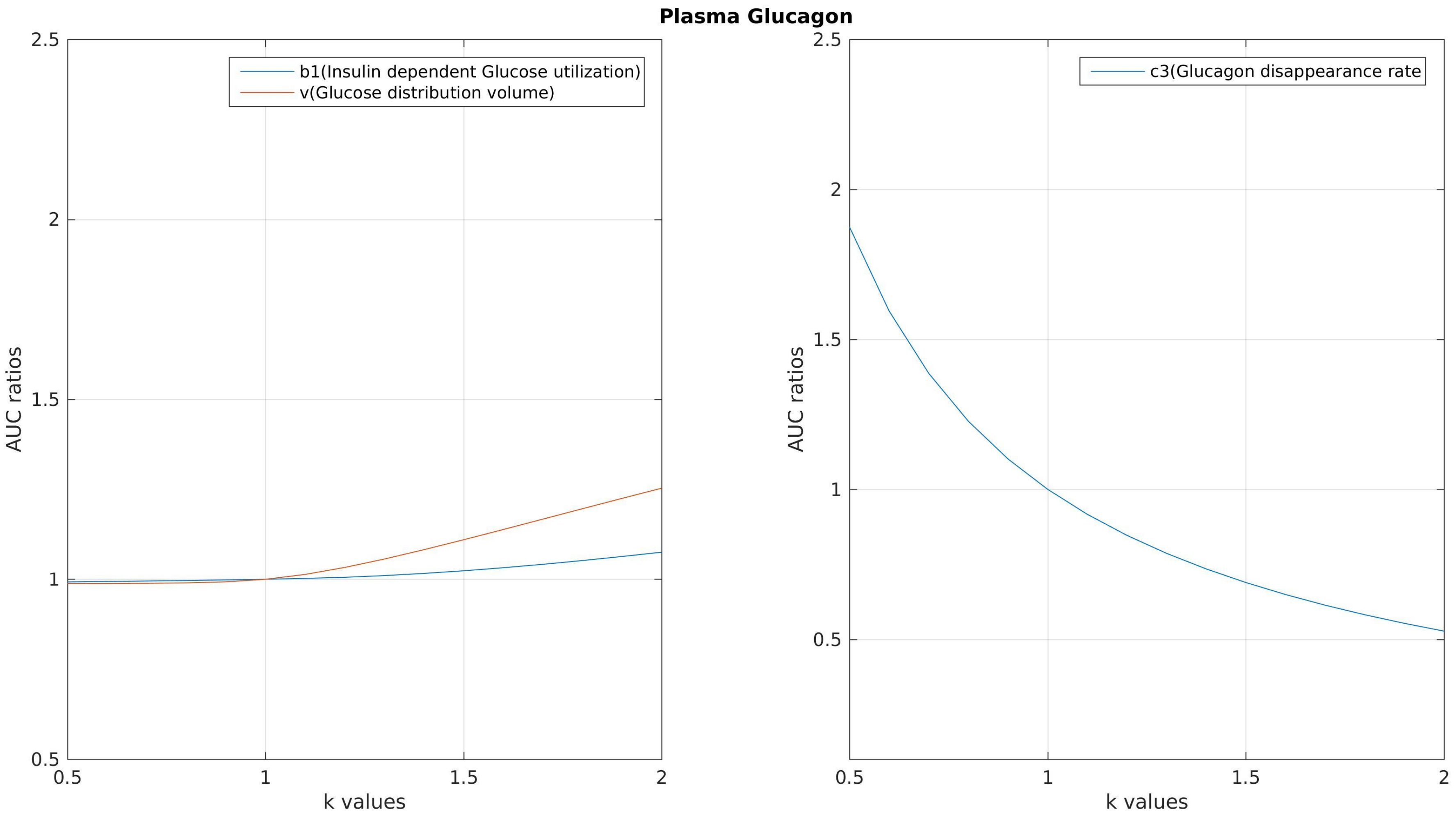

Plasma Glucagon
b1(Insulin dependent Glucose utilization)
v(Glucose distribution volume)
c3(Glucagon disappearance rate
AUC ratios
k values
AUC ratios
k values

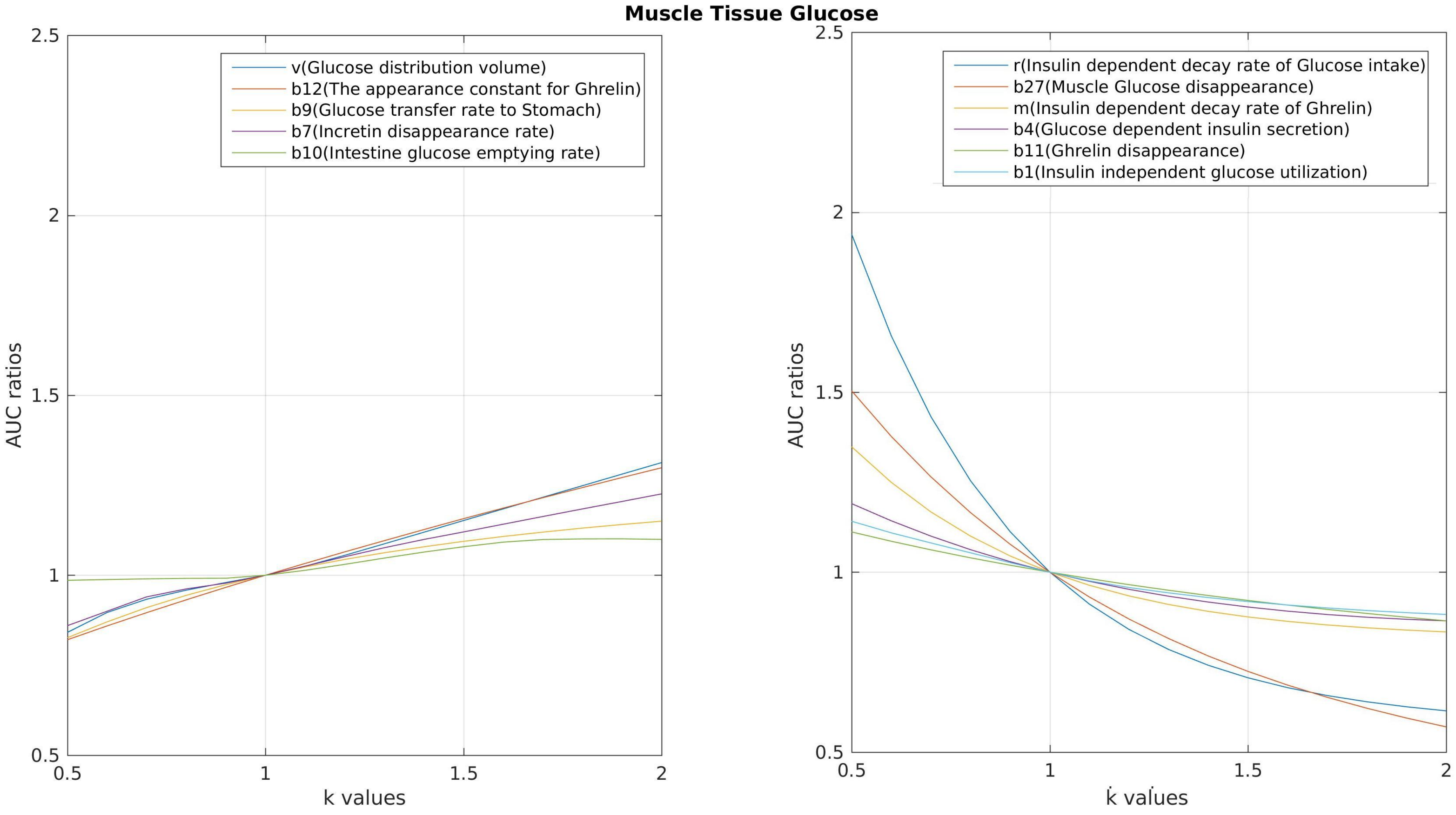
Muscle Tissue Glucose
AUC ratios
k values
v(Glucose distribution volume)
b12(The appearance constant for Ghrelin)
b9(Glucose transfer rate to Stomach)
b7(Incretin disappearance rate)
b10(Intestine glucose emptying rate)
r(Insulin dependent decay rate of Glucose intake)
b27(Muscle Glucose disappearance)
m(Insulin dependent decay rate of Ghrelin)
b4(Glucose dependent insulin secretion)
b11(Ghrelin disappearance)
b1(Insulin independent glucose utilization)
0.5
1
1.5
2
2.5

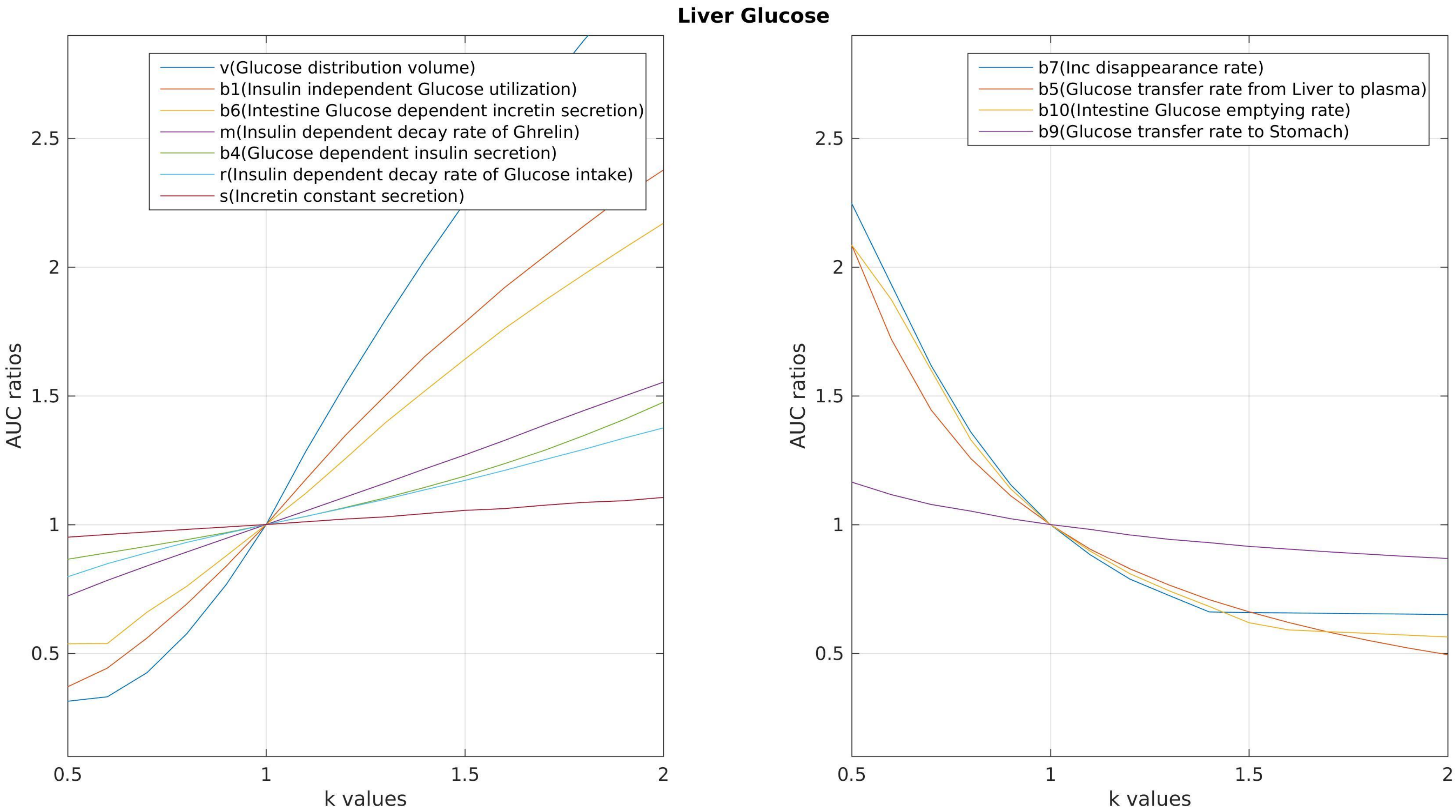

Liver Glucose
v(Glucose distribution volume)
b1(Insulin independent Glucose utilization)
b6(Intestine Glucose dependent incretin secretion)
m(Insulin dependent decay rate of Ghrelin)
b4(Glucose dependent insulin secretion)
r(Insulin dependent decay rate of Glucose intake)
s(Incretin constant secretion)
AUC ratios
k values
0.5
1
1.5
2
2.5
b7(Inc disappearance rate)
b5(Glucose transfer rate from Liver to plasma)
b10(Intestine Glucose emptying rate)
b9(Glucose transfer rate to Stomach)
AUC ratios
k values

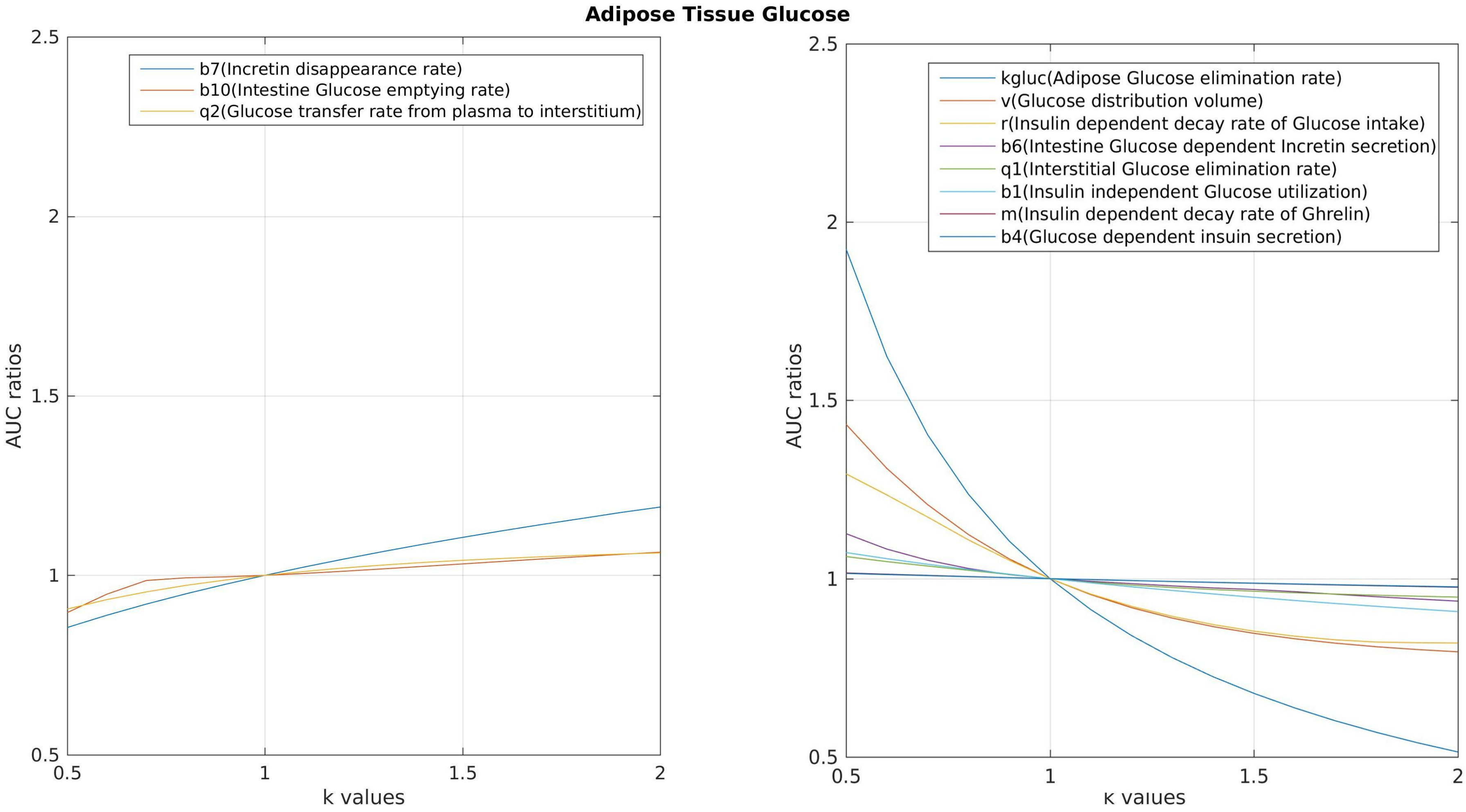
Adipose Tissue Glucose
b7(Incretin disappearance rate)
b10(Intestine Glucose emptying rate)
q2(Glucose transfer rate from plasma to interstitium)
AUC ratios
k values
kgluc(Adipose Glucose elimination rate)
v(Glucose distribution volume)
r(Insulin dependent decay rate of Glucose intake)
b6(Intestine Glucose dependent Incretin secretion)
q1(Interstitial Glucose elimination rate)
b1(Insulin independent Glucose utilization)
m(Insulin dependent decay rate of Ghrelin)
b4(Glucose dependent insuin secretion)
AUC ratios
k values

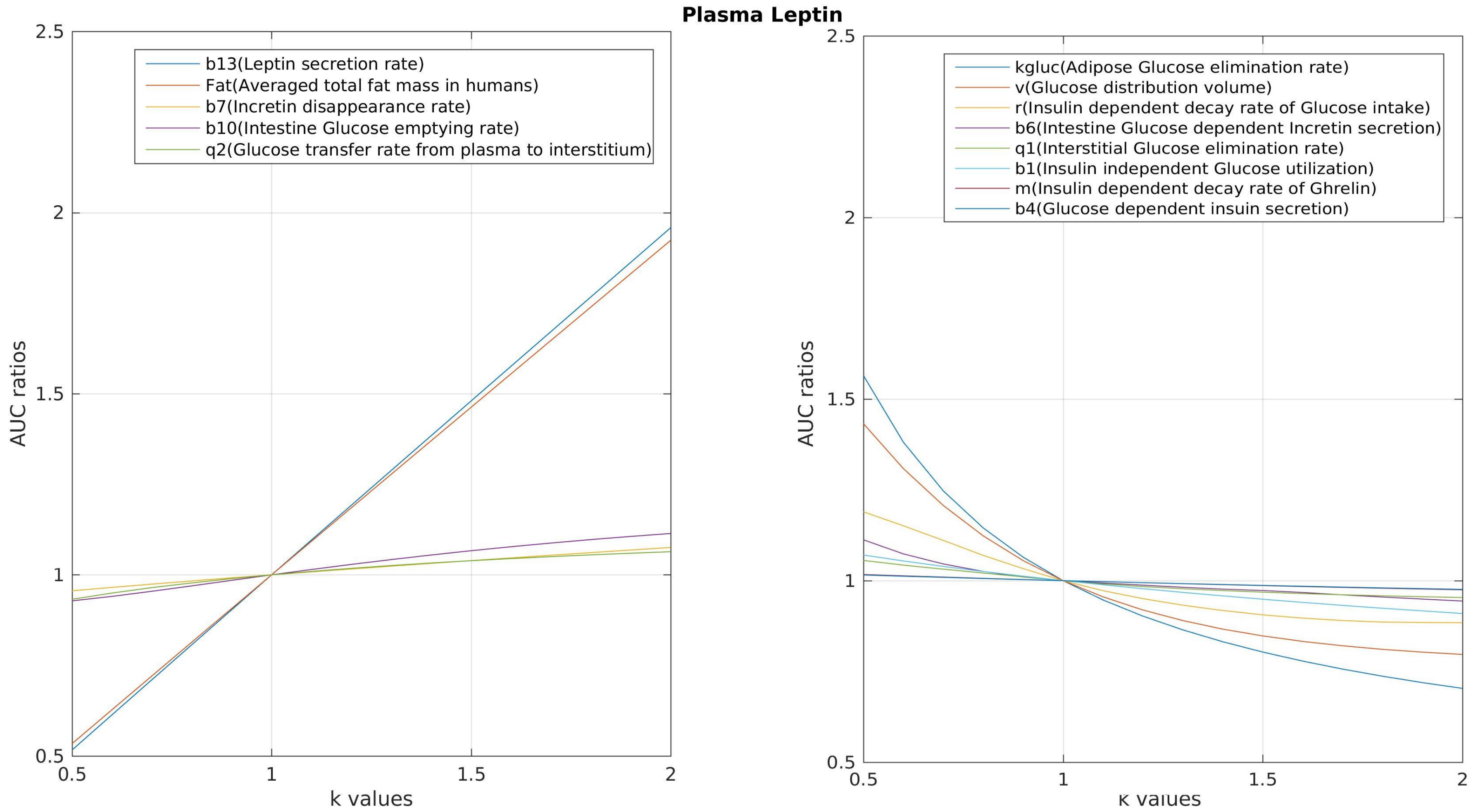
Plasma Leptin
b13(Leptin secretion rate)
Fat(Averaged total fat mass in humans)
b7(Incretin disappearance rate)
b10(Intestine Glucose emptying rate)
q2(Glucose transfer rate from plasma to interstitium)
AUC ratios
k values
kgluc(Adipose Glucose elimination rate)
v(Glucose distribution volume)
r(Insulin dependent decay rate of Glucose intake)
b6(Intestine Glucose dependent Incretin secretion)
q1(Interstitial Glucose elimination rate)
b1(Insulin independent Glucose utilization)
m(Insulin dependent decay rate of Ghrelin)
b4(Glucose dependent insuin secretion)
AUC ratios
k values

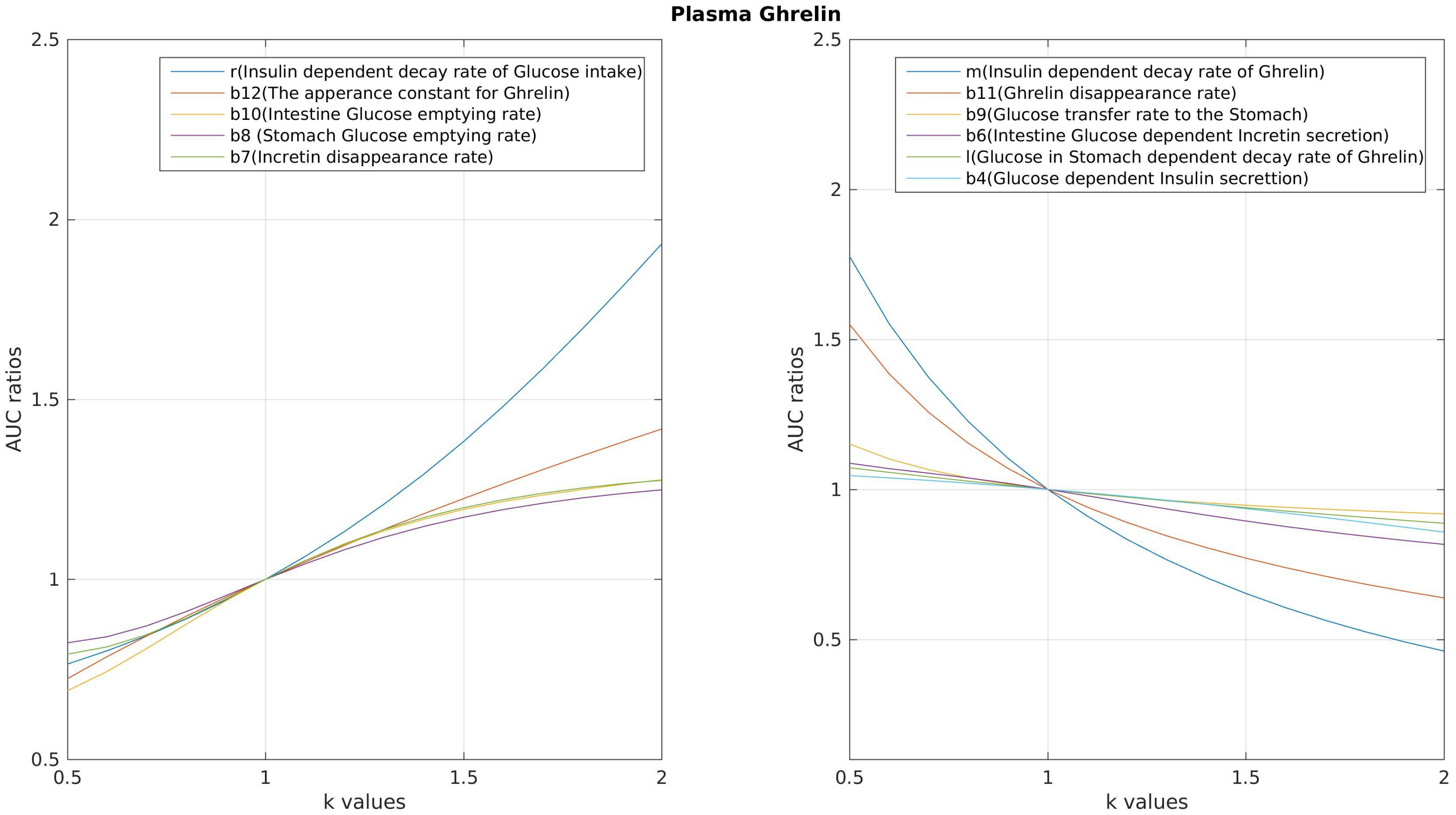
Plasma Ghrelin
r(Insulin dependent decay rate of Glucose intake)
b12(The apperance constant for Ghrelin)
b10(Intestine Glucose emptying rate)
b8 (Stomach Glucose emptying rate)
b7(Incretin disappearance rate)
AUC ratios
k values
m(Insulin dependent decay rate of Ghrelin)
b11(Ghrelin disappearance rate)
b9(Glucose transfer rate to the Stomach)
b6(Intestine Glucose dependent Incretin secretion)
l(Glucose in Stomach dependent decay rate of Ghrelin)
b4(Glucose dependent Insulin secrettion)
AUC ratios
k values
