# Supplementary material for: A closed-loop multi-level model of glucose homeostasis
Source: PLoS One. 2018 Feb 8;13(2):e0190627. doi: 10.1371/journal.pone.0190627 (PMC5805234; doi:10.1371/journal.pone.0190627)
Supplement: S3 File — (PDF) [file pone.0190627.s003.pdf]

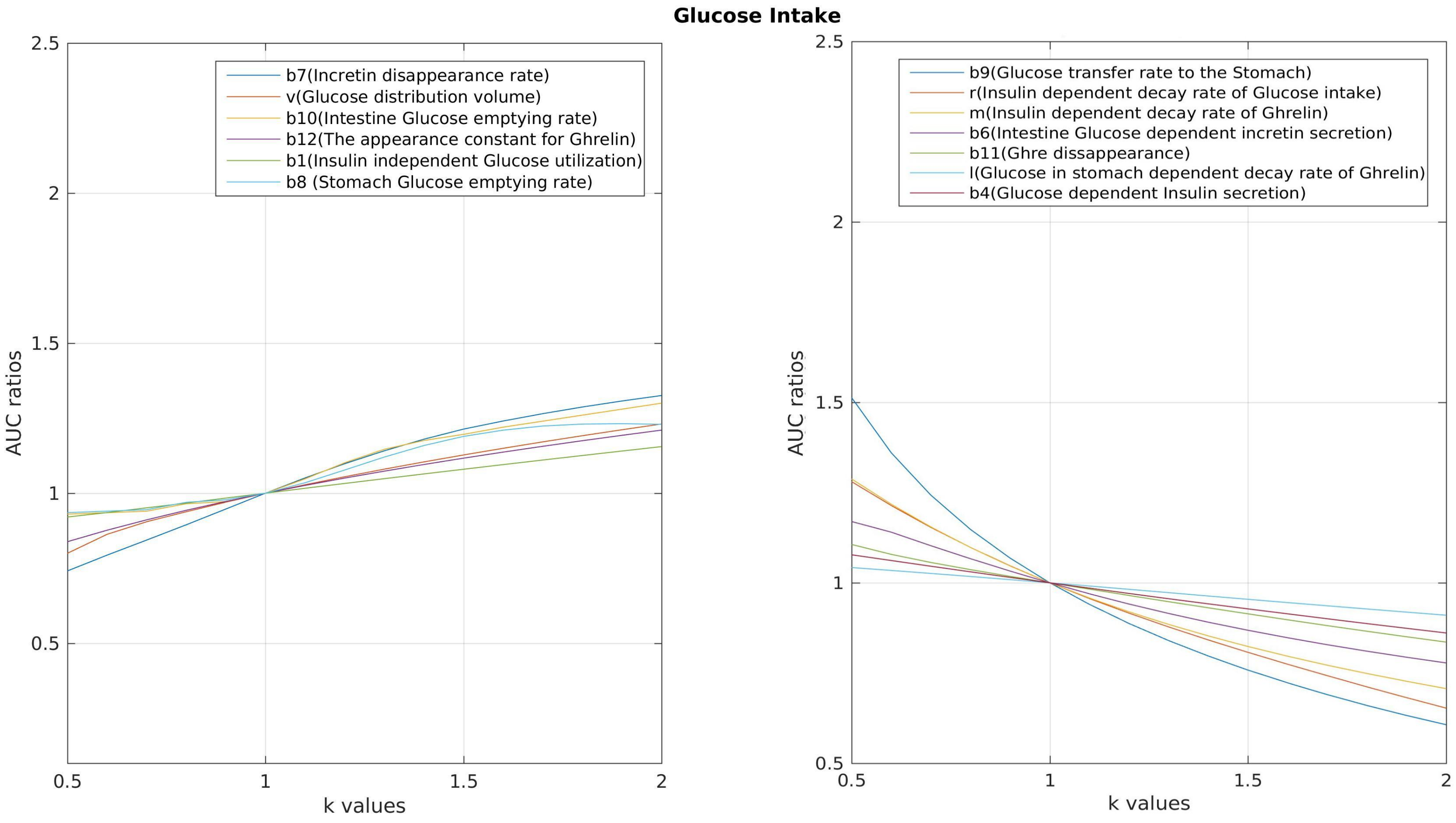
Glucose Intake
AUC ratios
k values
0.5
1
1.5
2
2.5
b7(Incretin disappearance rate)
v(Glucose distribution volume)
b10(Intestine Glucose emptying rate)
b12(The appearance constant for Ghrelin)
b1(Insulin independent Glucose utilization)
b8 (Stomach Glucose emptying rate)
b9(Glucose transfer rate to the Stomach)
r(Insulin dependent decay rate of Glucose intake)
m(Insulin dependent decay rate of Ghrelin)
b6(Intestine Glucose dependent incretin secretion)
b11(Ghre dissappearance)
l(Glucose in stomach dependent decay rate of Ghrelin)
b4(Glucose dependent Insulin secretion)

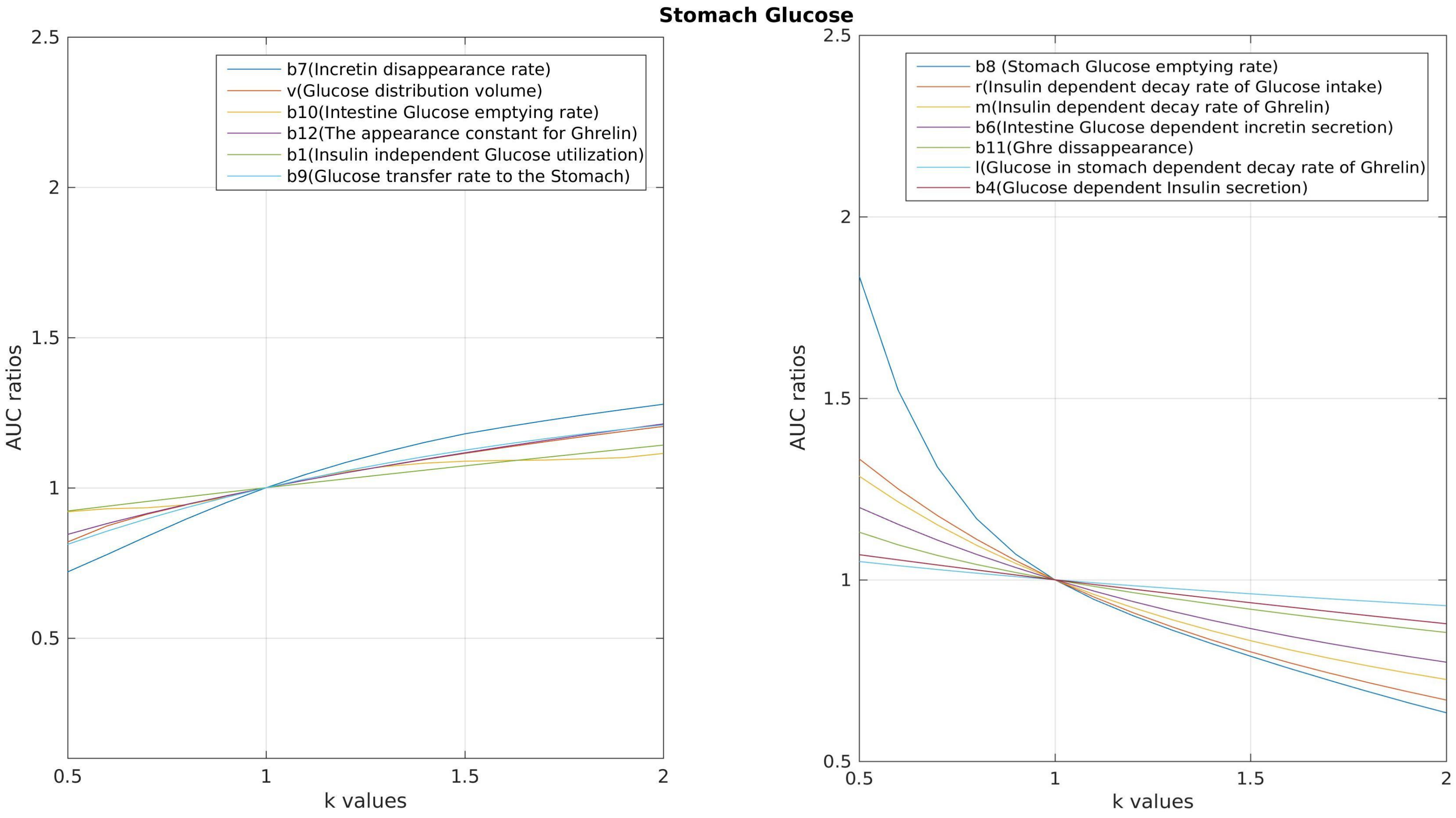
Stomach Glucose
b7(Incretin disappearance rate)
v(Glucose distribution volume)
b10(Intestine Glucose emptying rate)
b12(The appearance constant for Ghrelin)
b1(Insulin independent Glucose utilization)
b9(Glucose transfer rate to the Stomach)
AUC ratios
k values
b8 (Stomach Glucose emptying rate)
r(Insulin dependent decay rate of Glucose intake)
m(Insulin dependent decay rate of Ghrelin)
b6(Intestine Glucose dependent incretin secretion)
b11(Ghre dissappearance)
l(Glucose in stomach dependent decay rate of Ghrelin)
b4(Glucose dependent Insulin secretion)
AUC ratios
k values

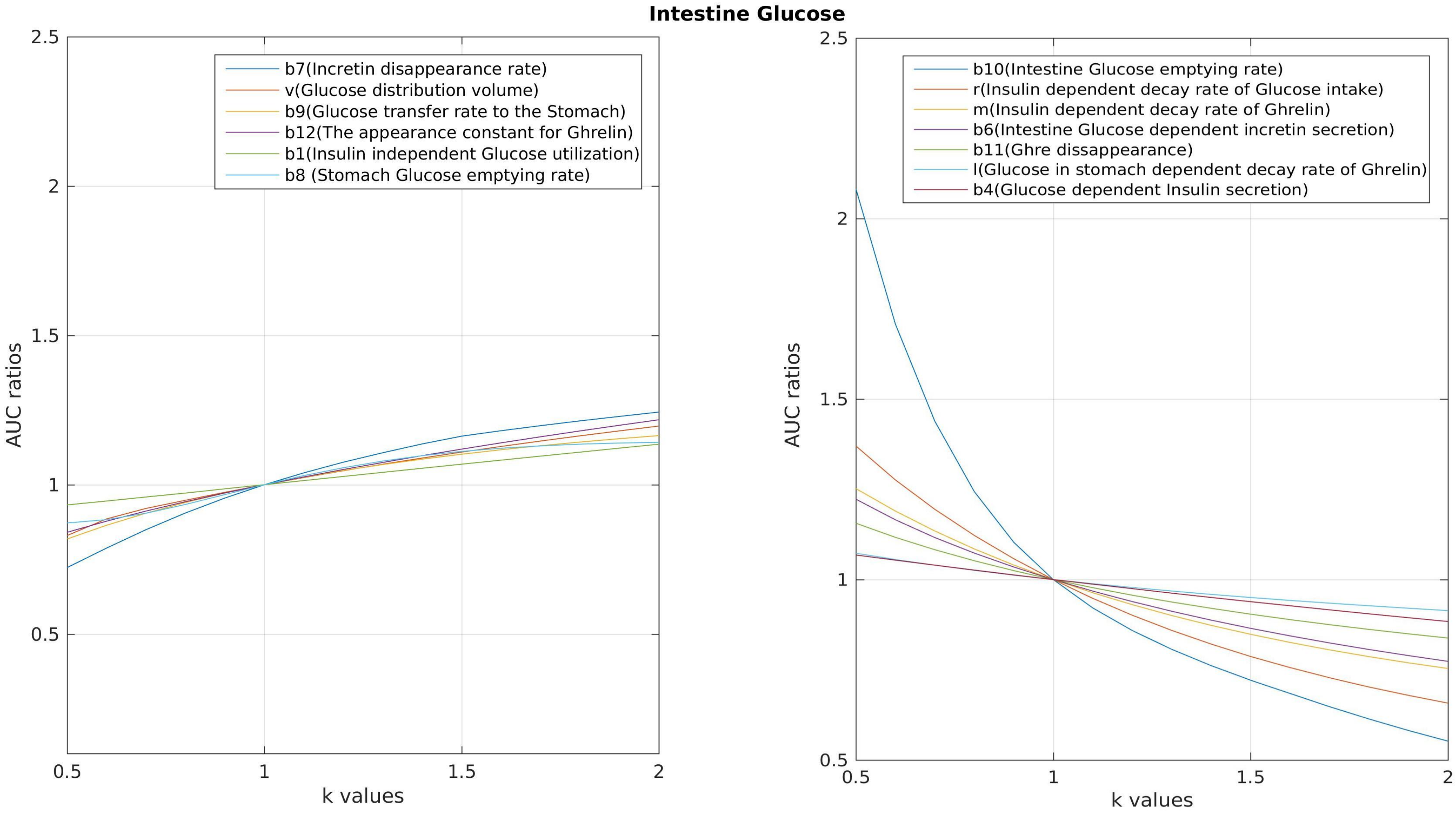

Intestine Glucose
AUC ratios
k values
b7(Incretin disappearance rate)
v(Glucose distribution volume)
b9(Glucose transfer rate to the Stomach)
b12(The appearance constant for Ghrelin)
b1(Insulin independent Glucose utilization)
b8 (Stomach Glucose emptying rate)
b10(Intestine Glucose emptying rate)
r(Insulin dependent decay rate of Glucose intake)
m(Insulin dependent decay rate of Ghrelin)
b6(Intestine Glucose dependent incretin secretion)
b11(Ghre dissappearance)
l(Glucose in stomach dependent decay rate of Ghrelin)
b4(Glucose dependent Insulin secretion)

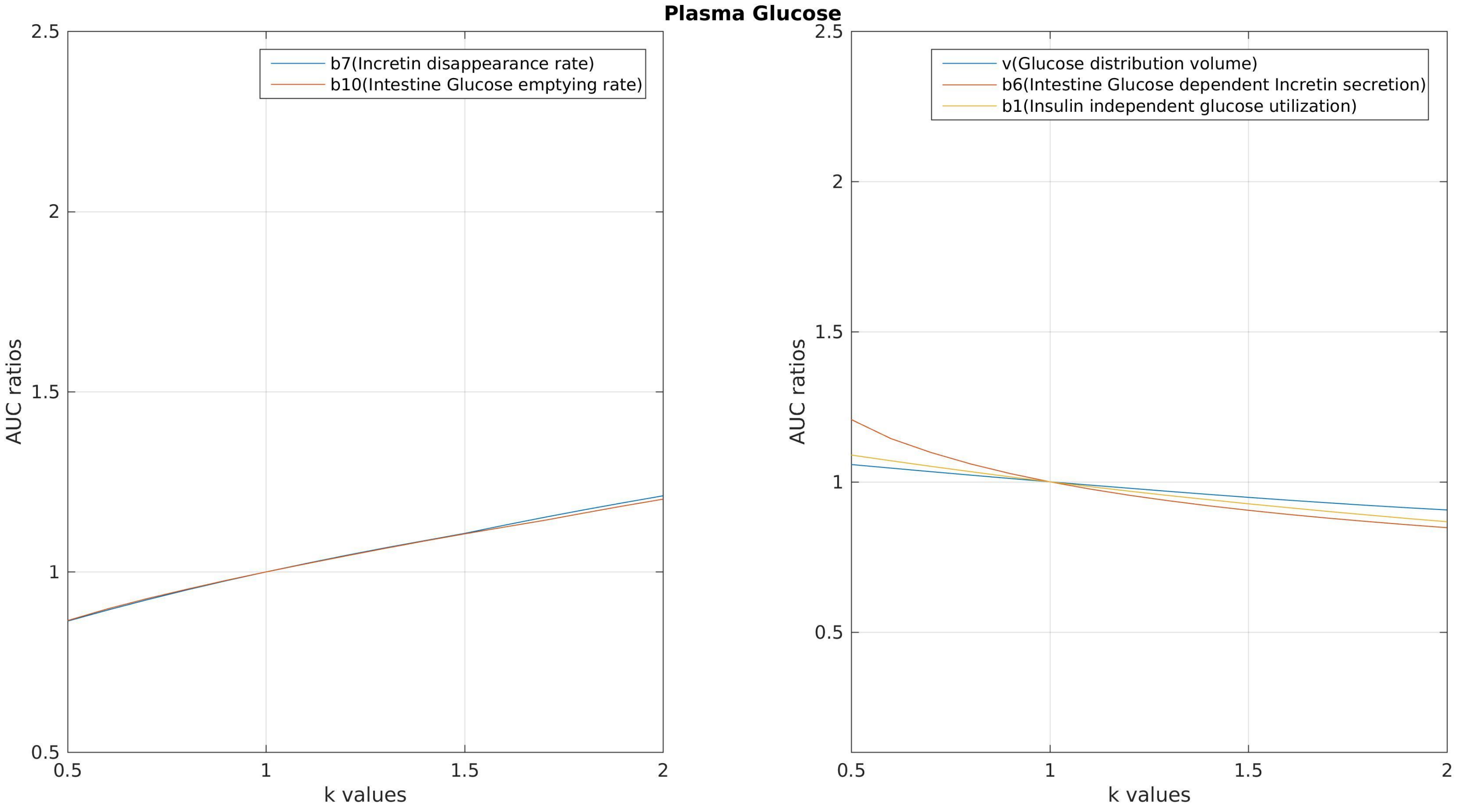
Plasma Glucose
b7(Incretin disappearance rate)
b10(Intestine Glucose emptying rate)
v(Glucose distribution volume)
b6(Intestine Glucose dependent Incretin secretion)
b1(Insulin independent glucose utilization)
AUC ratios
k values
0.5
1
1.5
2
2.5

Plasma Insulin

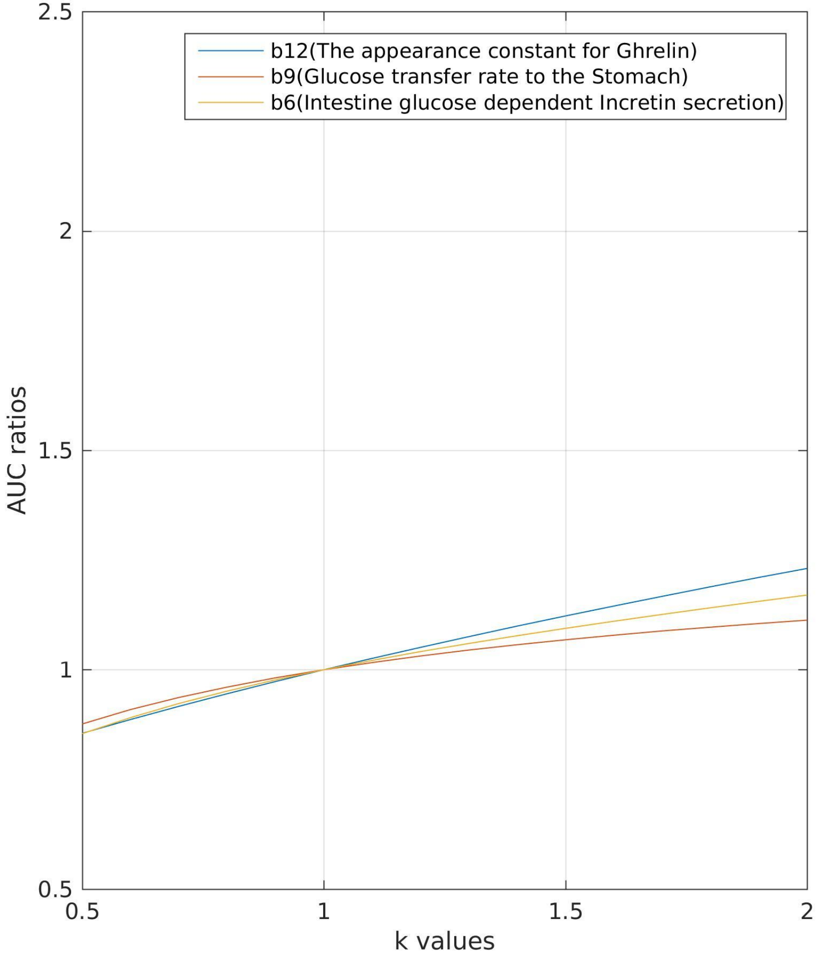

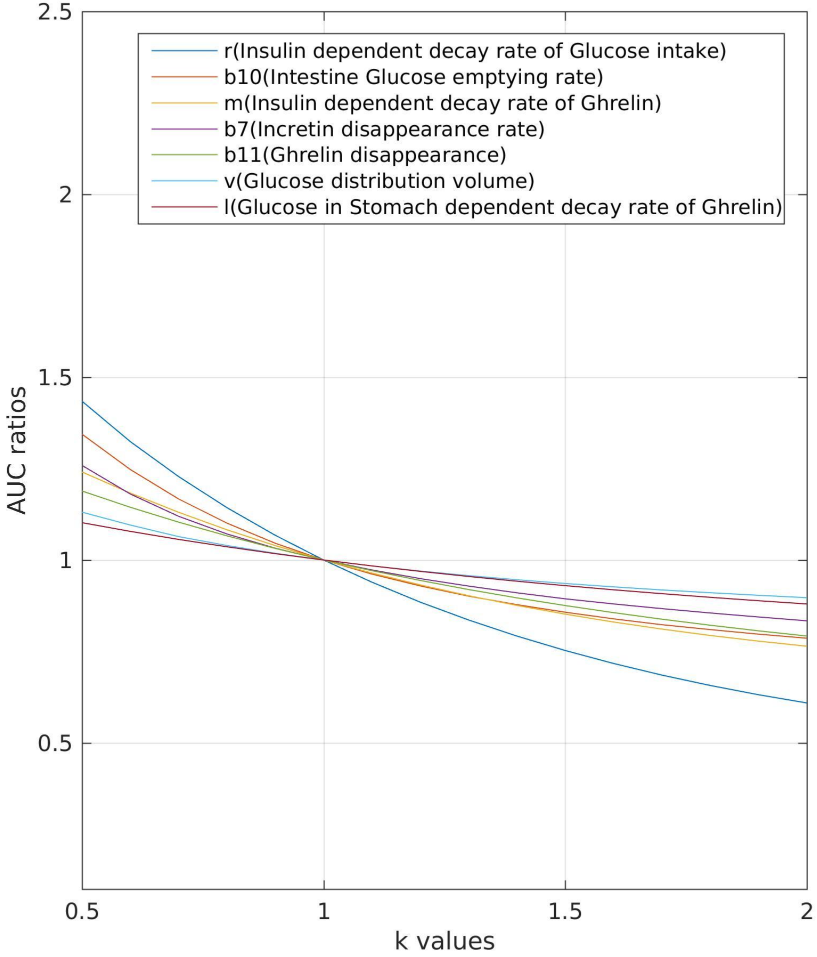

Plasma Incretin

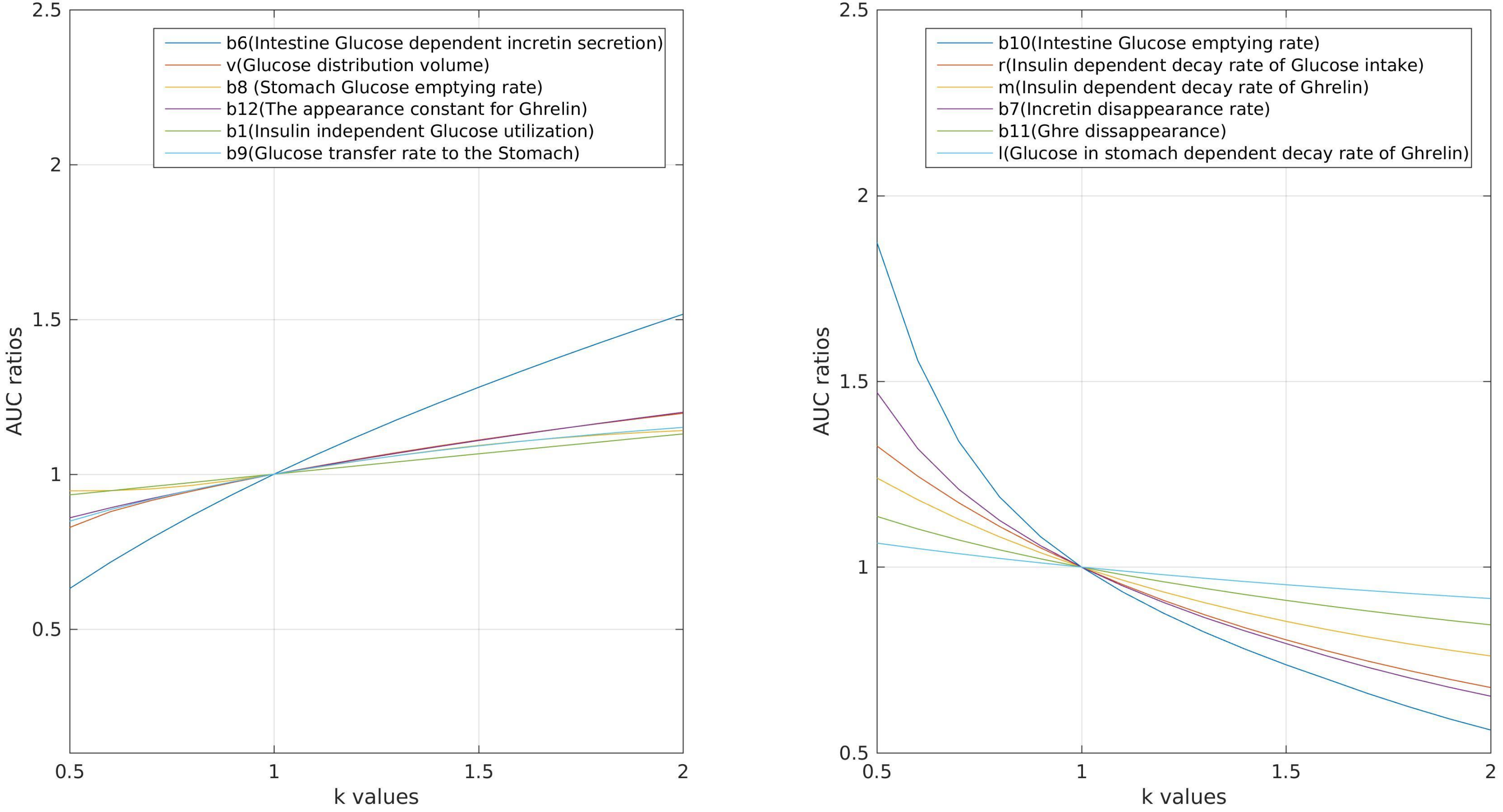

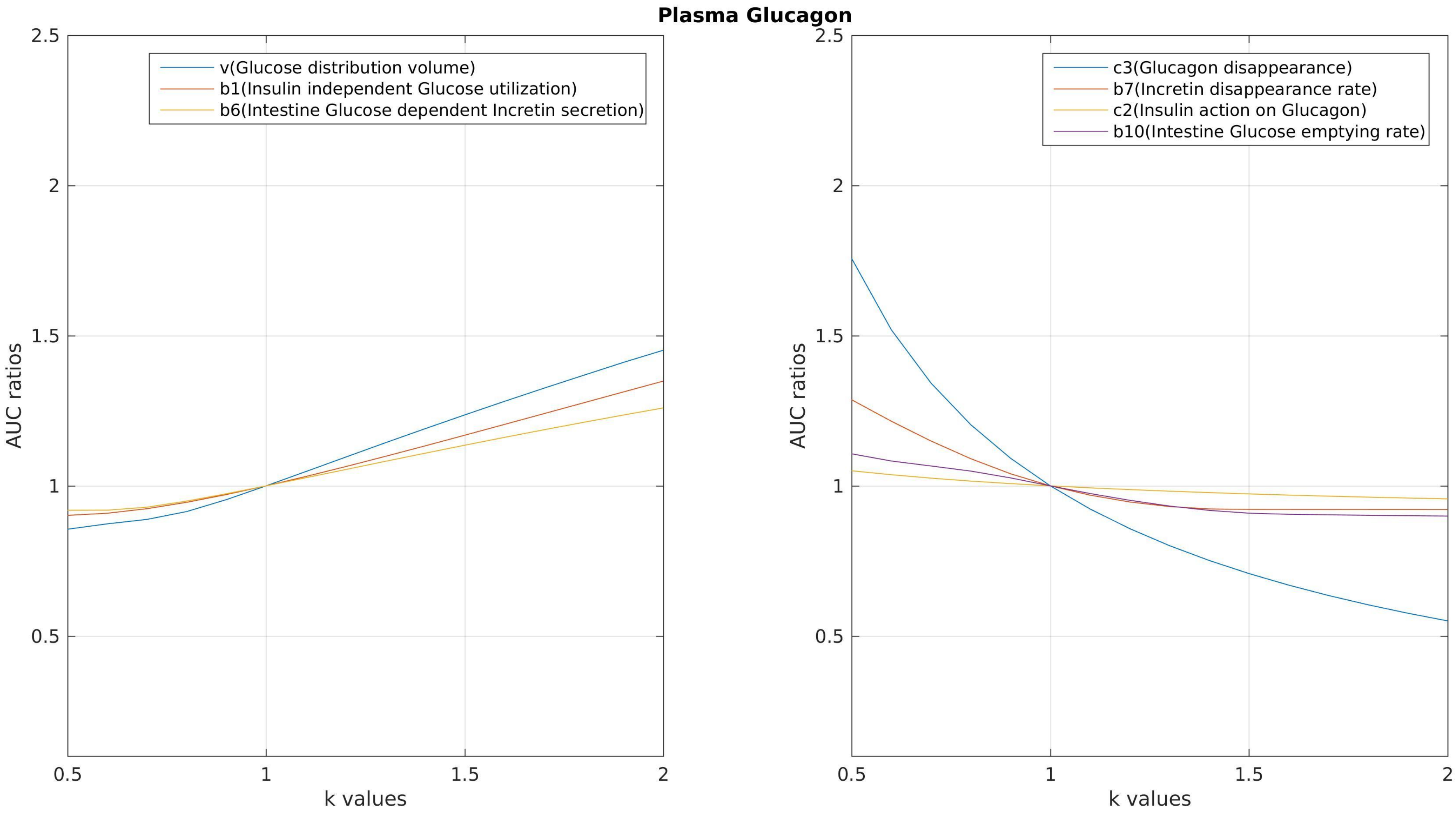
Plasma Glucagon
v(Glucose distribution volume)
b1(Insulin independent Glucose utilization)
b6(Intestine Glucose dependent Incretin secretion)
c3(Glucagon disappearance)
b7(Incretin disappearance rate)
c2(Insulin action on Glucagon)
b10(Intestine Glucose emptying rate)
AUC ratios
k values
2.5
2
1.5
1
0.5
0.5
1
1.5
2

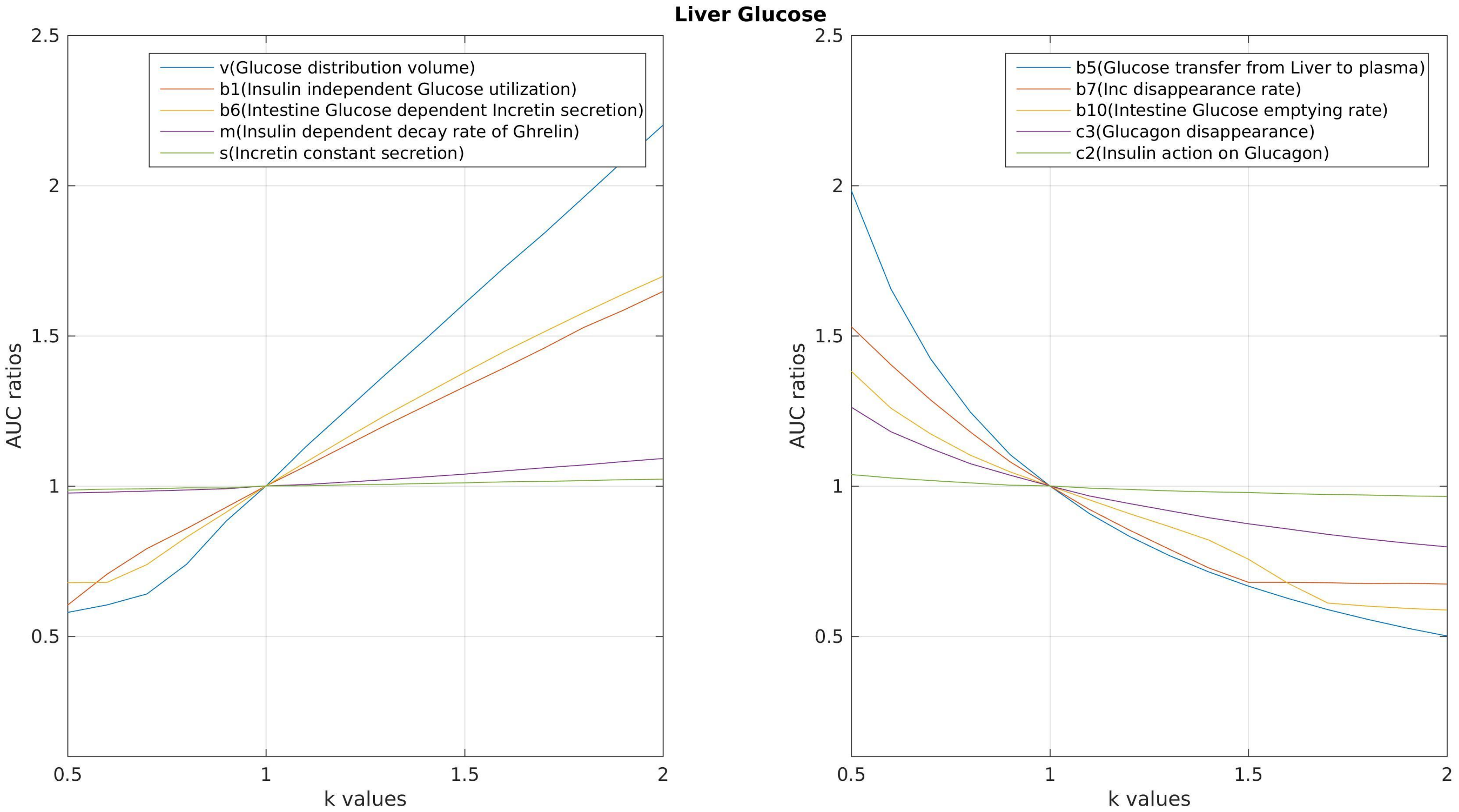
Liver Glucose
AUC ratios
k values
v(Glucose distribution volume)
b1(Insulin independent Glucose utilization)
b6(Intestine Glucose dependent Incretin secretion)
m(Insulin dependent decay rate of Ghrelin)
s(Incretin constant secretion)
b5(Glucose transfer from Liver to plasma)
b7(Inc disappearance rate)
b10(Intestine Glucose emptying rate)
c3(Glucagon disappearance)
c2(Insulin action on Glucagon)

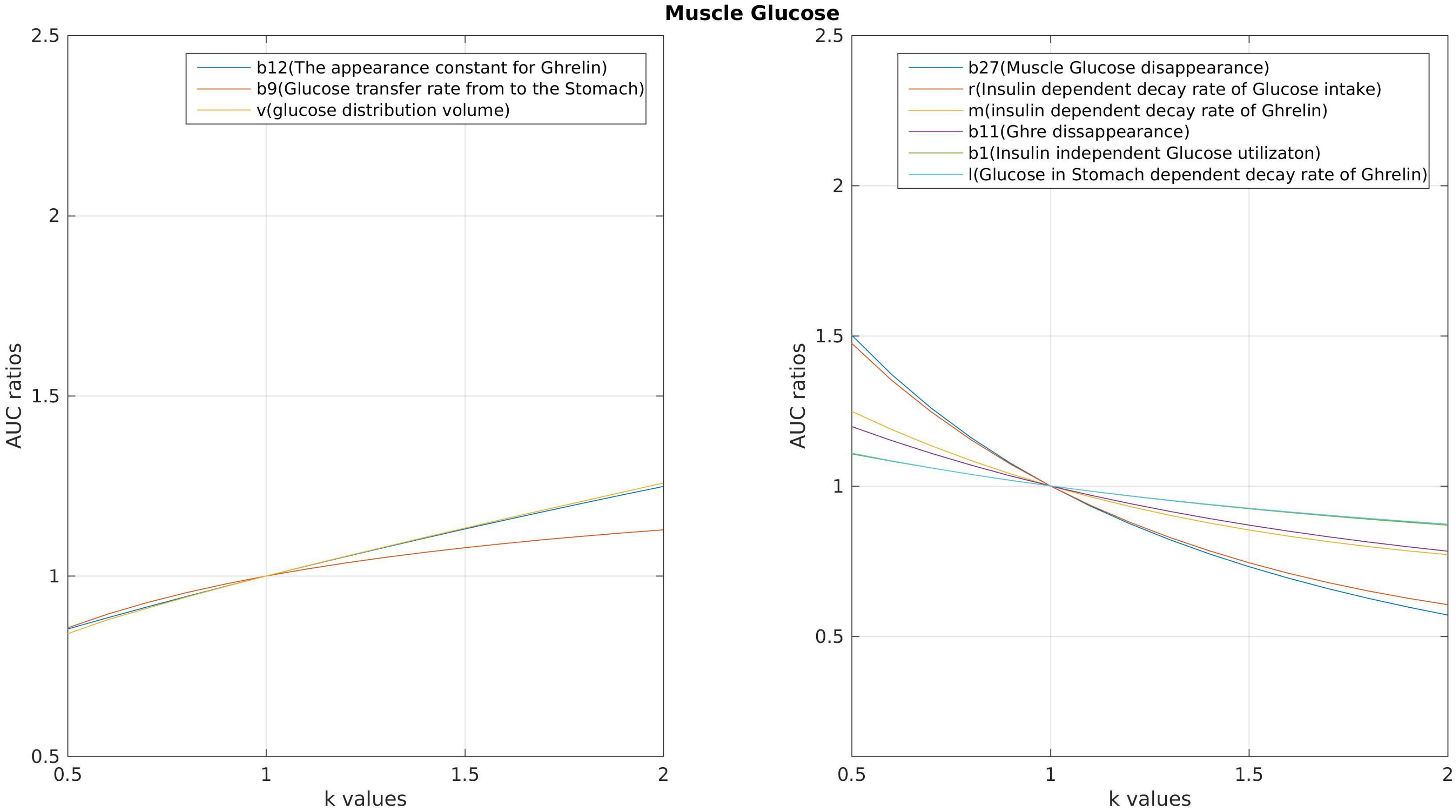

Muscle Glucose
AUC ratios
k values
b12(The appearance constant for Ghrelin)
b9(Glucose transfer rate from to the Stomach)
v(glucose distribution volume)
b27(Muscle Glucose disappearance)
r(Insulin dependent decay rate of Glucose intake)
m(insulin dependent decay rate of Ghrelin)
b11(Ghre dissappearance)
b1(Insulin independent Glucose utilizaton)
l(Glucose in Stomach dependent decay rate of Ghrelin)

Adipose Glucose

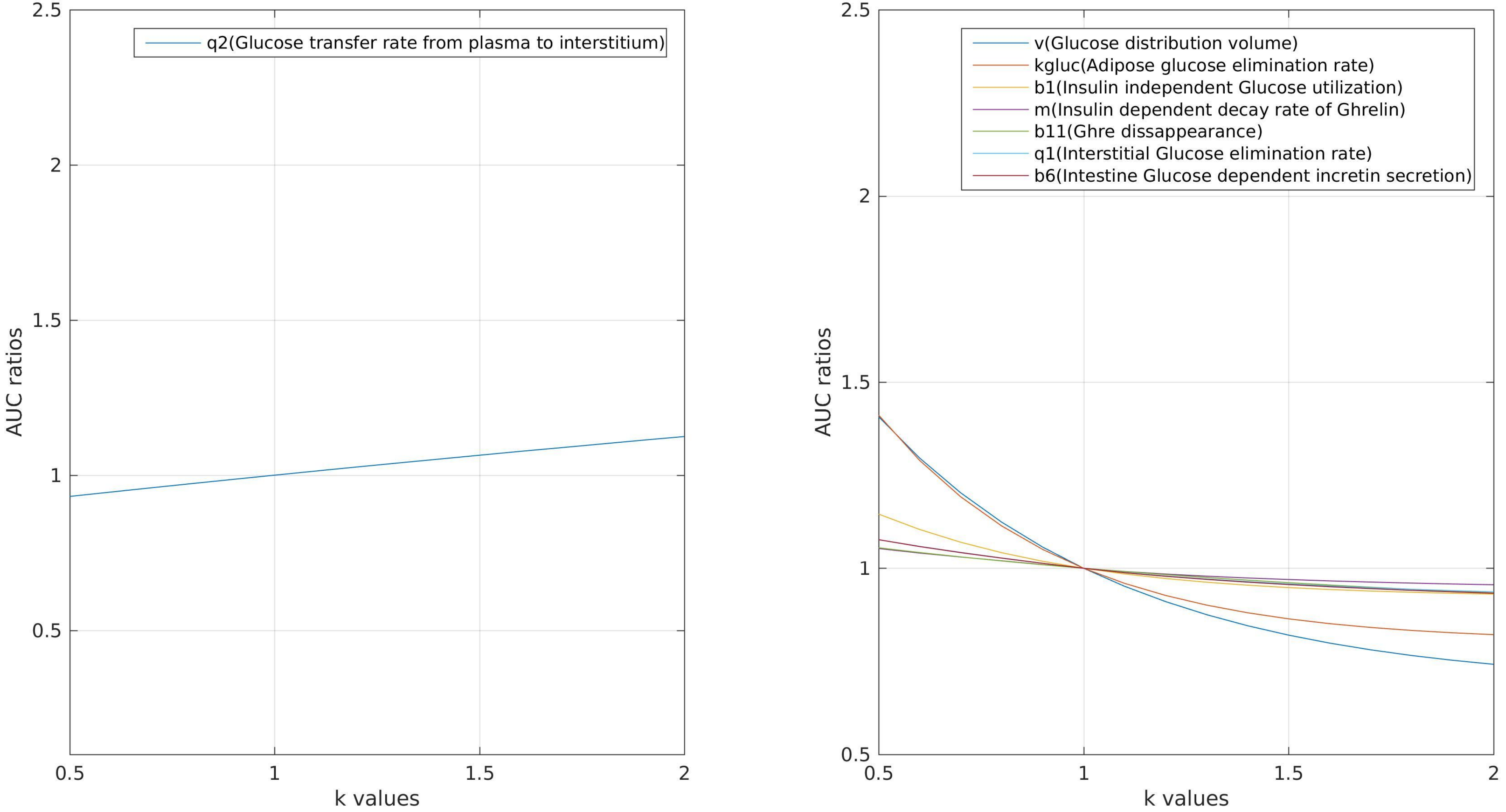

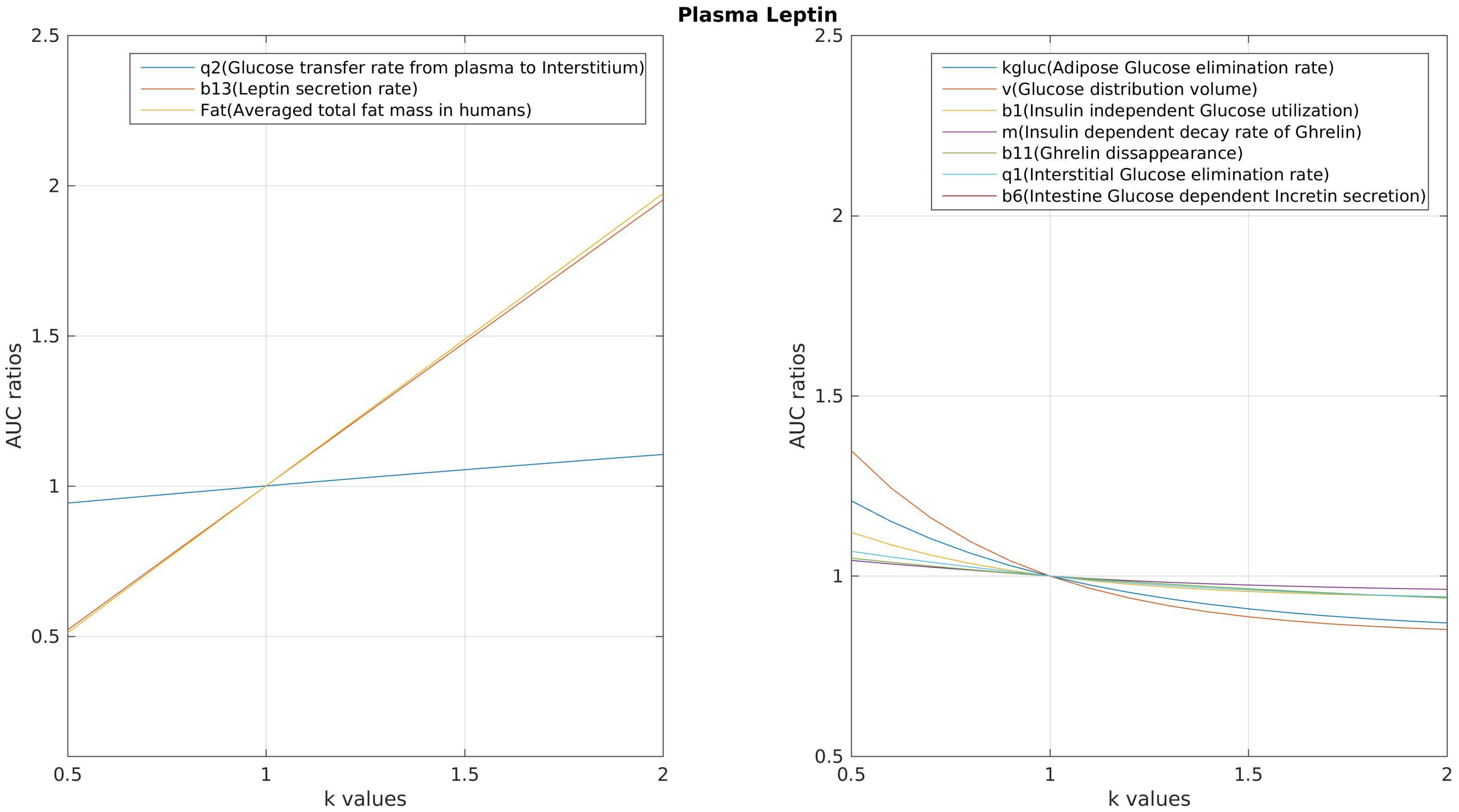
Plasma Leptin
AUC ratios
k values
q2(Glucose transfer rate from plasma to Interstitium)
b13(Leptin secretion rate)
Fat(Averaged total fat mass in humans)
kgluc(Adipose Glucose elimination rate)
v(Glucose distribution volume)
b1(Insulin independent Glucose utilization)
m(Insulin dependent decay rate of Ghrelin)
b11(Ghrelin dissappearance)
q1(Interstitial Glucose elimination rate)
b6(Intestine Glucose dependent Incretin secretion)

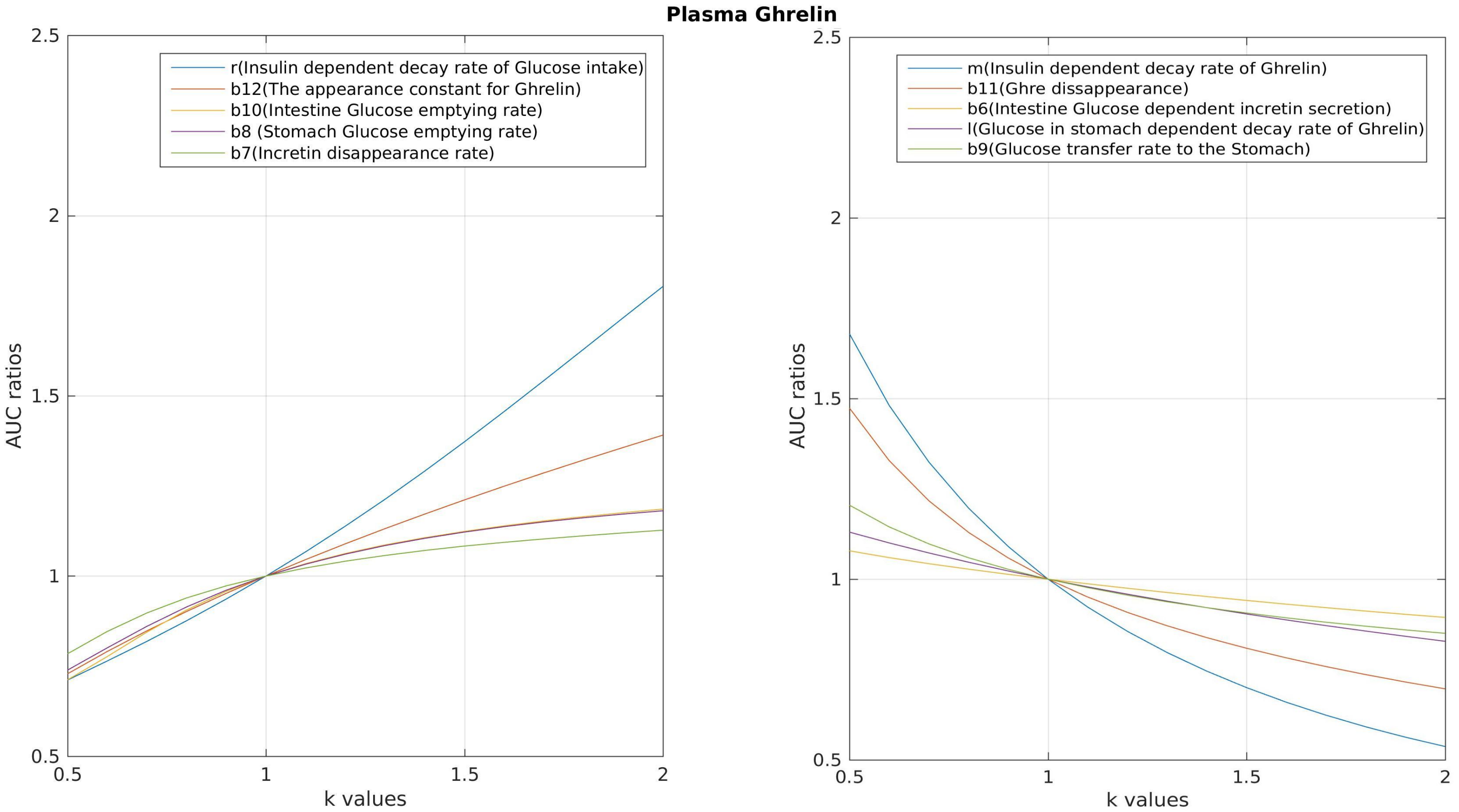

Plasma Ghrelin
AUC ratios
k values
r(Insulin dependent decay rate of Glucose intake)
b12(The appearance constant for Ghrelin)
b10(Intestine Glucose emptying rate)
b8 (Stomach Glucose emptying rate)
b7(Incretin disappearance rate)
m(Insulin dependent decay rate of Ghrelin)
b11(Ghre dissappearance)
b6(Intestine Glucose dependent incretin secretion)
l(Glucose in stomach dependent decay rate of Ghrelin)
b9(Glucose transfer rate to the Stomach)
